# Does the Choice of Stepping Intensity Metric Influence Dose-Response Associations with Mortality? Analysis on UK Population Cohort Study of 65,253 Adults

**DOI:** 10.1249/MSS.0000000000003887

**Published:** 2025-10-27

**Authors:** LE WEI, MATTHEW N. AHMADI, JOANNA M. BLODGETT, ELROY J. AGUIAR, RAAJ KISHORE BISWAS, NICHOLAS A. KOEMEL, BORJA DEL POZO CRUZ, EMMANUEL STAMATAKIS

**Affiliations:** 1 Mackenzie Wearables Research Hub, Charles Perkins Centre, The University of Sydney, Sydney, NSW, AUSTRALIA; 2 School of Health Sciences, Faculty of Medicine and Health, The University of Sydney, Sydney, NSW, AUSTRALIA; 3 Charles Perkins Centre, Faculty of Medicine and Health, The University of Sydney, Sydney, NSW, AUSTRALIA; 4 Institute of Sport, Exercise and Health, Division of Surgery and Interventional Sciences, University College London, London, UNITED KINGDOM; 5 Biomedical Research Centre, University College London Hospitals NIHR, London, UNITED KINGDOM; 6 Department of Kinesiology, The University of Alabama, Tuscaloosa, AL; 7 Department of Sport Sciences, Faculty of Medicine, Health, and Sports, Universidad Europea de Madrid, Madrid, SPAIN

**Keywords:** AVERAGE CADENCE, METRICS, PEAK CADENCE, STEPPING INTENSITY

## Abstract

**Background:**

Evidence on the potential mortality gain of higher free-living stepping intensity is limited and equivocal, potentially due to the inconsistent usage among various estimation metrics. To estimate the difference in the association with mortality risk across different stepping intensity metrics, we aimed to compare different metrics in terms of their multivariable-adjusted associations with all-cause, cardiovascular disease, and cancer mortality.

**Methods:**

This cohort study included UK Biobank participants wearing wrist-worn accelerometers. We included eight peak cadence metrics, defined as the highest averaged steps/min across eight different time windows (1-, 5-, 10-, 15-, 20-, 25-, 30-, and 60-min), and two nonpeak cadence metrics including average daily cadence (steps/accelerometer wearing minutes) and purposeful cadence (averaged steps/min for minutes ≥40 steps). For each metric, we first standardized each individual’s absolute cadence using (individual’s absolute cadence − mean cadence)/standard deviation. We then estimated their dose-response associations using Cox-restricted-cubic-spline models and compared them on overlay plots.

**Results:**

Among 65,253 participants (mean age: 61.5 yr [standard deviation: 7.8]; 57% female) followed for 8.0 (median) yr, all peak cadence metrics and the average daily cadence exhibited similar positive dose-response associations with mortality. For example, the medians of the individual-level standardized cadence and hazard ratios (HRs) across peak 1-, 30-, and 60-min cadence were: all-cause mortality, −0.17 steps/min (HR: 0.71 [95% confidence interval (CI): 0.64–0.80]), −0.15 (0.66 [0.59–0.74]), and −0.15 (0.66 [0.59–0.75]), respectively; cardiovascular disease mortality, −0.17 steps/min (HR: 0.63 [95% CI: 0.51–0.78]), −0.15 (0.57 [0.46–0.71]), and −0.15 (0.57 [0.46–0.71]); cancer mortality, −0.17 steps/min (HR: 0.88 [95% CI: 0.75–1.03]), −0.15 (0.89 [0.75–1.04]), −0.16 (0.93 [0.78–1.09]). Purposeful cadence was not associated with mortality (e.g., all-cause mortality: median of the individual-level standardized cadence, 0.59 steps/min; HR, 0.99 [95% CI: 0.86–1.15]).

**Conclusions:**

This study suggested that peak cadence and average cadence metrics can be used interchangeably to quantify the associations of stepping intensity with long-term health outcomes.

Steps are a fundamental component of daily physical activities (PAs) (1) and have the advantage of being easily captured in a free-living environment using wearables or smartphones (2). Major stepping activities such as walking and running are easily accessible for most adults (3,4). Hence, step-related research has gained particular attention. While the evidence supporting the mortality gain of higher daily stepping amount (steps/day) is relatively consistent (5,6), evidence regarding that of stepping intensity (6) remains limited and inconsistent. Compared to the daily step amount (i.e., steps/day), metrics for estimating stepping intensity (e.g., peak cadence metrics) are inconsistent and complex to understand (5,7). Lack of understanding on the difference between the metrics may create uncertainty for metric selection, a pivotal methodological consideration for researchers aiming to establish robust step-related prospective evidence (8). Among the limited number of studies examining the dose-response association of higher stepping intensity with mortality risks, inconsistency in the metrics used to estimate free-living stepping intensity may have contributed to current equivocal evidence on the magnitude of the associations with mortality (5,9). Although prior studies have also examined the dose-response associations with the incidence of cardiovascular disease (CVD) (10,11), cancer (10,12), diabetes (13), and dementia (14), it remains unclear whether the use of inconsistent metrics influences the magnitude of dose-response association of stepping intensity with prospective health outcomes. This inconsistency may hinder the establishment of evidence on the magnitude of the association of stepping intensity with prospective mortality outcomes.

Different stepping intensity metrics reflect distinct aspects of stepping intensity. Peak cadence metrics—defined as the average steps per minute during the highest, but not necessarily consecutive minutes of the day (15)—can differ in duration (5,7,15,16) and thus reflect stepping intensity differently. For example, short metrics such as peak 10-min cadence focused on the intensity of the fastest 10 min of steps, whereas long metrics such as peak 60-min cadence may capture additional mortality gain due to lower intensity (e.g., light intensity stepping) or brief incidental steps. However, recent device-based studies have highlighted the vital role of higher intensity PA (e.g., MVPA) for premature mortality prevention (17), which is more likely to be reflected in peak cadence metrics of shorter durations. In contrast, light-intensity PA revealed much smaller mortality gain (e.g., the equivalence of light against each minute of moderate intensity and vigorous intensity for all-cause mortality (ACM) was 10:1 and 50:1, respectively (18)). Prior studies have revealed that peak cadence metrics—regardless of short or long duration—displayed significant and consistent link with key health risk factors such as age, body mass index, systolic blood pressure, glucose, insulin, high-density lipoprotein, and triglycerides (15,19,20). Collectively, these findings raise the question: does the choice of metric matter when estimating the mortality gain of higher stepping intensity?

Among the limited evidence for stepping intensity, peak 30-min and 60-min cadence were inversely associated with lower risk of ACM in a large harmonized meta-analysis (5), whereas other studies limited to older women (7) and middle-aged and older adults (9) did not exhibit such an association using peak 1-min and peak 30-min cadence. Another cohort study indicates that average daily cadence, defined as total daily step counts divided by total valid accelerometer wear minutes, was inversely associated with ACM risk in older adults (21). However, different sample characteristics and methodology (e.g., different selection of confounders, reference level setting) in each study precluded researchers from understanding the difference among the various stepping intensity metrics. Therefore, a comparison of a comprehensive selection of stepping intensity metrics under the same conditions (i.e., using the same cohort dataset and methodology) is needed.

Accordingly, we explored and compared the dose-response associations of ten different stepping intensity metrics, including eight peak-cadence metrics and two other cadence-based metrics, with all-cause, CVD, and cancer mortality in a large prospective cohort of UK adults. We hypothesized that the ten stepping intensity metrics would exhibit a similar dose-response association with all-cause, CVD, and cancer mortality, respectively.

## METHODS

### Participants

The UK Biobank is a large prospective cohort with 502,616 UK adults aged 40–69 yr recruited between 2006 and 2010 (22). Ethical approval for this cohort study was provided by the UK National Health Service, National Research Ethics Service (Ref 11/NW/0382). We selected the participants based on prior literature examining the association between stepping intensity and health outcomes (7,10). We excluded participants with prevalent CVD or cancer history (ascertained through hospital admission records), missing covariate data, insufficient valid wear time, body mass index less than 18.5, frailty, and those with a death event within 2 yr after the PA baseline assessment. All participants provided written informed consent.

### Step-Related Metrics Assessment

From 2013 to 2015, 103,684 UK Biobank participants were mailed and wore the Axivity AX3 (Axivity Ltd, Newcastle Upon Tyne, United Kingdom) wrist-worn triaxial accelerometer on their dominant wrist for 7 days continuously. The AX3 was initialized to capture triaxial acceleration data at a sampling frequency of 100 Hz and a dynamic range of ±8g. Nonwear periods were identified based on standard procedure (23,24). A monitoring day was considered valid if the wear time exceeded 16 h (10). Participants needed at least three valid monitoring days, including a minimum of one weekend day to be included (10,25,26). We calculated steps during periods of ambulation using a tuned signal peak detection method (27–29) with step-detection accuracy of 89%, total steps mean absolute percent error of 10% (28), and a mean bias of 9% (30). This method based on wrist-worn accelerometers has shown step-detection accuracy of 89% and total steps mean absolute percent error of 10% when compared to video-recorded data (27). A recent UK biobank study applying the same method (29) has demonstrated high accuracy (e.g., intraclass correlation of 0.86 [0.77–0.92], mean absolute percent error of 10.6%, mean bias of 103 [±152] steps) in 60 participants when compared to ground-truth data using video direct observation or a thigh-worn monitor that has a 99% accuracy with directly observed steps data. Furthermore, we evaluated accuracy under different walking conditions; with mean absolute percent error of roughly 10% during free-living walking, demonstrating the feasibility of applying this step-detection method in free-living settings. This step-detection method has also been used in prior step-related UK biobank studies (10,14).

### Calculation of Stepping Intensity Metrics

#### Peak-cadence metrics: peak 1-, 5-, 10-, 15-, 20-, 25-, 30-, and 60-min cadence

First, minute-by-minute step data were rank-ordered from the highest to lowest (15) for each valid accelerometer wear day. Then, we selected the highest steps/min for 1, 5, 10, 15, 20, 25, 30, and 60 min, and calculated the average steps/min over the corresponding time interval for each valid wear day. Finally, we averaged them over the total number of valid wear days.

#### Average daily cadence and purposeful cadence

 To calculate the average daily cadence (steps/min), we divided the total step counts by the total valid accelerometer wear minutes for each valid wear day, and then calculated the mean over the total valid wear days (21). To calculate the average cadence of purposeful steps, we used the >40 steps per minute threshold as per previous literature (5,7,10) and summed the total step counts from these minutes and divided by the total wear minutes for each day, then averaged these values across all valid wear days.

### Outcome Ascertainment

Participants were followed up to November 30, 2022, with deaths obtained through linkage with the National Health Service (NHS) Digital of England and Wales or the National Records of Scotland. Based on ICD-10 codes from both primary and contributory death causes, we defined CVD mortality as death from diseases of the circulatory system (ICD-10 codes: I0, I11, I13, I20–I51, and I60–I69), excluding hypertension and diseases of arteries and lymph (31). We defined cancer mortality as death attributed to any cancer excluding in situ, benign, uncertain, nonmelanoma skin cancers, or nonwell-defined cancers (ICD-10 codes: C0-C6, C70-C75, C7A, C8, C9) (32).

### Covariates

Based on similar peer-reviewed literature examining the association between stepping intensity and mortality (7,9,10), all analyses were adjusted for age, sex, ethnicity, valid accelerometer wear days, smoking status, alcohol consumption, sleep duration, screen-time, fruit and vegetable consumption, education level, economic status, family history of CVD and cancer, medication use on cholesterol, blood pressure and diabetes, and daily step count using the residual method (5,33).

### Statistical Analysis

We excluded data below 1st and above 99th percentile of the distribution across cadence-based metrics to minimize the influence of sparse data (10). We transformed the cadence values using the standardization method, calculated as (absolute – mean)/standard deviation (SD) (32,34–36) across cadence-based metrics to rescale them for cross comparison. Each metric was centered around a mean of 0 with an SD of 1. We assessed time-to-event dose-response associations of standardized stepping intensity with ACM using Cox-restricted-cubic-spline models. For cause-specific outcomes, we used the Fine and Grey model to account for competing risks (10,37). We set the reference level at the 5^th^ percentile (29) and placed knots at 10th, 50th, and 90th percentiles of the exposures’ distributions (38,39). We assessed proportional hazard assumptions through Schoenfeld residuals and observed no violation. We presented overlay dose-response plots on a scale of standardized cadence for visual comparison between the dose-response associations across stepping intensity metrics on the same scale. For better comparisons, we assessed differences in effect size at the standardized cadence of −0.5 at the lower stratum of the standardized cadence scale, and at 3.0 at the upper stratum. We presented the distribution of each metric to demonstrate the appropriateness of using standardization scaling. We repeated the above analysis using absolute cadence to assess the dose-response association with mortality outcomes for easier interpretation of findings. We computed cadence doses (i.e., absolute peak cadence) at the median cadence, minimum cadence (i.e., 50% of the optimal risk reduction), and the optimal cadence (i.e., optimal risk reduction) (39).

We assessed the robustness of our findings with four sensitivity analyses: First, we presented overlay dose-response plots on the scale using normalized cadence, an alternative to the standardized cadence calculated as (cadence - minimum cadence)/ (maximum cadence - minimum cadence) (36). Second, we performed analysis using a weighted sample to improve representativeness of the sample (40). Third, a sex-specific analysis was conducted. Fourth, we conducted an analysis based on step-defined PA level strata (i.e., 0–5000 steps/day as physically inactive; 5000–7500 as low active; 7500–10,000 as somewhat active; above 10,000 steps/day as physically active (41)).

We performed all analyses using *R* statistical software (version 4.3.1) with the cph function in rms package (6.8.0).

## RESULTS

### Description of the Study Sample

Our sample for peak 30-min cadence included 65,253 participants (mean [SD] age, 61.5 [7.8] yr; female, 37,316 [57%]) followed up for a median of 8.0 yr (SD: 0.9) and 520,782 person-years. Of these, a total of 1736 participants died (CVD: 466; cancer: 1038) (Supplemental Table 1, Supplemental Digital Content, http://links.lww.com/MSS/D319). The distributions of standardized stepping intensity metrics were similar (Supplemental Figs. 2 and 3, Supplemental Digital Content, http://links.lww.com/MSS/D319).

### Association of Stepping Intensity with All-Cause Mortality

**FIGURE 1:**
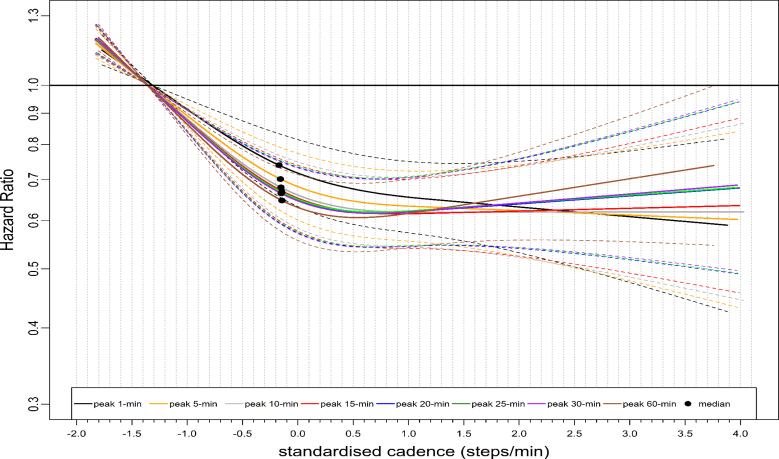
Dose-response association of standardized stepping intensity estimated across peak cadence metrics with all-cause mortality. Total sample size is 65,253. The events for peak 1-min cadence are 1739; peak 5-min cadence, 1734; peak 10-min cadence, 1735; peak 15-min cadence, 1735; peak 20-min cadence, 1736; peak 25-min cadence, 1734; peak 30-min cadence, 1736; peak 60-min cadence, 1734. The standardized cadence was calculated as ([peak cadence − mean]/standard deviation). The circle indicates the median standardized cadence. We analyzed the dose-response associations using cox-regression model and adjusted for age, sex, accelerometer wearing duration, average daily steps, smoking status, alcohol consumption, sleep duration, Townsend deprivation score, sedentary time, education levels, self-reported parental history of CVD and cancer, and self-reported medication use (cholesterol, blood pressure, and diabetes). The reference level was set as the 5th percentile of each peak cadence metric.

**FIGURE 2:**
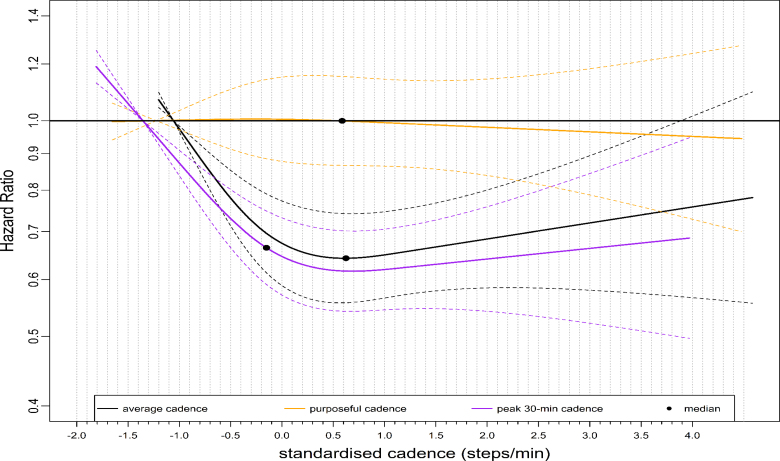
Dose-response association of standardized stepping intensity estimated across nonpeak cadence metrics (average daily cadence, purposeful cadence) and peak 30-min cadence with all-cause mortality. We compared two nonpeak cadence metrics and a representative peak cadence metric (peak 30-min cadence) in this figure. Total sample size is 65,253. Events for the average daily cadence is 1736; purposeful cadence, 1772; peak 30-min cadence, 1736; The circle indicates the median standardized cadence. We analyzed the dose-response associations using cox-regression model and adjusted for age, sex, accelerometer wearing duration, average daily steps, smoking status, alcohol consumption, sleep duration, Townsend deprivation score, sedentary time, education levels, self-reported parental history of CVD and cancer, and self-reported medication use (cholesterol, blood pressure, and diabetes). The reference level was set as the 5th percentile of each stepping intensity metric.

We observed similar L-shaped dose-response associations with ACM across stepping intensity metrics, with 95% confidence intervals (CIs) largely overlapping (Fig. 1). For example, the median standardized cadence and the corresponding hazard ratio (HR) for peak 1- and 30-min cadence was −0.18 steps/min, 0.74 (95% CI: 0.66–0.83) and −0.15 steps/min, 0.66 (0.59–0.75), respectively (Table 1). At the lower end of the standardized cadence scale, for example, at −0.5 steps/min, the HR for peak 1-min sand peak 30-min cadence were 0.80 (95% CI: 0.72–0.87) and 0.73 (95% CI: 0.67–0.80), respectively (Table 1). HR corresponds to peak 1- and 30-min cadence at 3.0 standardized steps/min at the upper stratum was: 0.61 (95% CI: 0.48–0.78) and 0.66 (0.52–0.84), respectively (Table 1). The dose-response association of the average daily cadence with ACM revealed a steep L-shaped dose-response association (Fig. 2) similar to that of peak 30-min cadence. For example, the median point and the corresponding HR for the average cadence was 0.62 steps/min, 0.64 (95% CI: 0.56–0.74) (Table 1). However, purposeful cadence did not reveal a dose-response association with ACM (Fig. 2). For example, the standardized median and the corresponding HR for purposeful cadence were 0.59 steps/min, 0.99 (95% CI: 0.86–1.15) (Table 1).

**TABLE 1.** Hazard ratio of all-cause mortality at −0.5, 3.0, and median standardized steps per minute, estimated by peak cadence and nonpeak cadence metrics.

Peak Cadence Metrics	−0.5 Standardized Steps/Min	3.0 Standardized Steps/Min	Standardized Median Cadence	
	HR (95% CI)	HR (95% CI)	Steps/Min	HR (95% CI)
Peak 1-min cadence	0.80 (0.72, 0.87)	0.61 (0.48, 0.78)	−0.18	0.74 (0.66, 0.83)
Peak 5-min cadence	0.76 (0.69, 0.84)	0.61 (0.48, 0.78)	−0.16	0.70 (0.62, 0.79)
Peak 10-min cadence	0.74 (0.68, 0.82)	0.62 (0.48, 0.79)	−0.15	0.68 (0.60, 0.77)
Peak 15-min cadence	0.74 (0.67, 0.81)	0.63 (0.49, 0.80)	−0.15	0.67 (0.60, 0.76)
Peak 20-min cadence	0.74 (0.67, 0.80)	0.66 (0.52, 0.84)	−0.14	0.67 (0.59, 0.75)
Peak 25-min cadence	0.73 (0.67, 0.80)	0.66 (0.52, 0.84)	−0.15	0.67 (0.59, 0.75)
Peak 30-min cadence	0.73 (0.67, 0.80)	0.66 (0.52, 0.84)	−0.15	0.66 (0.59, 0.75)
Peak 60-min cadence	0.72 (0.65, 0.79)	0.74 (0.59, 0.93)	−0.15	0.65 (0.57, 0.73)
Purposeful cadence	1.00 (0.91, 1.11)	0.96 (0.79, 1.18)	0.59	0.99 (0.86, 1.15)
Average daily cadence	0.78 (0.71, 0.85)	0.72 (0.58, 0.89)	0.62	0.64 (0.56, 0.74)

For better comparison, we standardized the cadence across stepping intensity metrics to rescale them into comparable range. The standardized cadence was calculated as ([exposure − mean]/standard deviation). Each metric was centered around a mean of 0 with a standard deviation of 1.

### Association of Stepping Intensity with CVD Mortality

**FIGURE 3:**
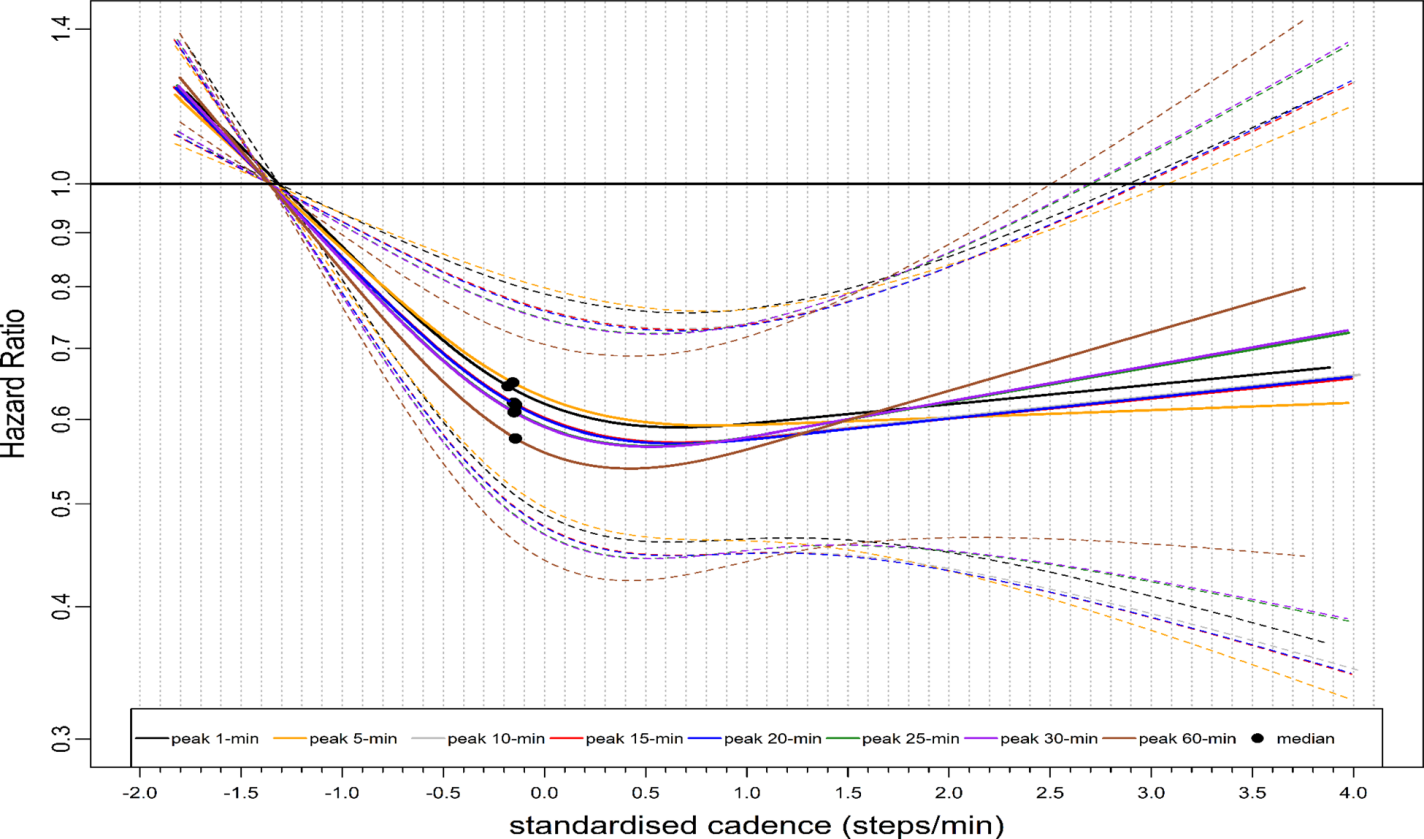
Dose-response association of standardized stepping intensity estimated across peak cadence metrics with CVD mortality. Total sample size is 65,253. The events for peak 1-min cadence are 468; peak 5-min cadence, 463; peak 10-min cadence, 465; peak 15-min cadence, 465; peak 20-min cadence, 465; peak 25-min cadence, 466; peak 30-min cadence, 466; peak 60-min cadence, 469; The standardized cadence was calculated as ([exposure − mean]/standard deviation). The circle indicates the standardized median cadence. We used Fine and Grey model to analyze the dose-response association and adjusted for age, sex, accelerometer wearing duration, average daily steps, smoking status, alcohol consumption, sleep duration, Townsend deprivation score, sedentary time, education levels, self-reported parental history of CVD and cancer, and self-reported medication use (cholesterol, blood pressure, and diabetes). The reference level is 5th percentile of each standardized peak cadence metric.

**FIGURE 4:**
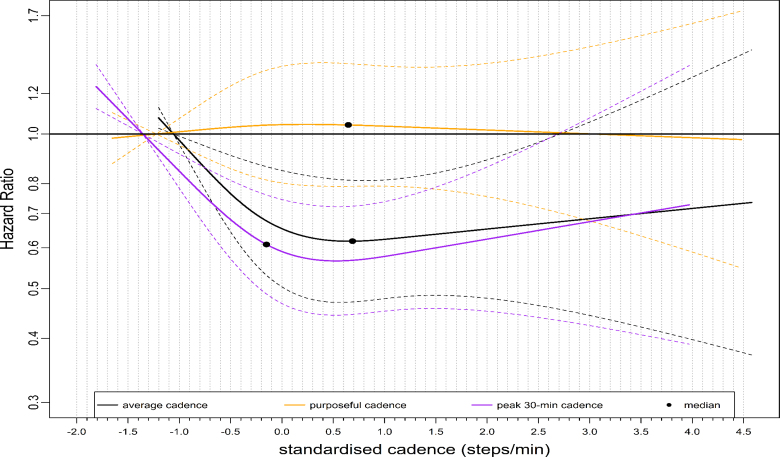
Dose-response association of standardized stepping intensity estimated by the average daily cadence, purposeful cadence, and peak 30-min cadence with CVD mortality. We compared two nonpeak cadence metrics and a representative peak cadence metric (peak 30-min cadence) in this figure. Total sample size is 65,253, the events for peak 30-min cadence are 466; average daily cadence, 463; average purposeful cadence, 484. The standardized cadence was calculated as ([exposure − mean]/standard deviation). The circle indicates the median standardized cadence. We used Fine and Grey model to analyze the dose-response association and adjusted for age, sex, accelerometer wearing duration, average daily steps, smoking status, alcohol consumption, sleep duration, Townsend deprivation score, sedentary time, education levels, self-reported parental history of CVD and cancer, and self-reported medication use (cholesterol, blood pressure, and diabetes). The reference level is 5th percentile of each standardized peak cadence metric.

The dose-response pattern for CVD mortality was similar to that of ACM, albeit with a more pronounced magnitude of risk reduction (Fig. 3). For example, the median standardized cadence and the corresponding HR for peak 1- and 30-min cadence was −0.18 steps/min, 0.64 (95% CI: 0.52–0.81) and −0.15 steps/min, 0.61 (0.49–0.76), respectively (Table 2). At the lower end of the standardized cadence scale, for example, at −0.5 standardized cadence, the HRs for peak 1-min and peak 30-min cadence were 0.71 (95% CI: 0.59–0.85) and 0.68 (95% CI: 0.57–0.81), respectively (Table 2). The HR for peak 1- and 30-min cadence at 3.0 standardized steps/min at the upper stratum was: 0.65 (95% CI: 0.41–1.02) and 0.67 (0.42–1.07), respectively (Table 2). The dose-response association of average daily cadence with CVD mortality revealed a steep L-shaped dose-response association (Fig. 4) similar to that of peak 30-min cadence. For example, the median point and the corresponding HR for average cadence were: 0.69 steps/min, 0.62 (95% CI: 0.47–0.81) (Table 2). However, purposeful cadence did not reveal a dose-response association with CVD mortality (Fig. 4). For example, the standardized median and the corresponding HR for purposeful cadence were 0.65 steps/min, 1.04 (95% CI: 0.79–1.37) (Table 2).

**TABLE 2.** Hazard ratio of CVD mortality at −0.5, 3.0, median standardized steps per minute, estimated by peak cadence and nonpeak cadence metrics.

Peak Cadence Metrics	−0.5 Standardized Steps/Min	3.0 Standardized Steps/Min	Standardized Median Cadence	
	HR (95% CI)	HR (95% CI)	steps/min	HR (95% CI)
Peak 1-min cadence	0.71 (0.59, 0.85)	0.65 (0.41, 1.02)	−0.18	0.64 (0.52, 0.81)
Peak 5-min cadence	0.71(0.59, 0.85)	0.61 (0.38, 0.99)	−0.16	0.65 (0.52, 0.81)
Peak 10-min cadence	0.69 (0.58, 0.83)	0.63 (0.39, 1.00)	−0.15	0.62 (0.50, 0.78)
Peak 15-min cadence	0.69(0.58, 0.83)	0.63(0.39, 1.00)	−0.16	0.62(0.50, 0.78)
Peak 20-min cadence	0.69 (0.58, 0.83)	0.63 (0.39, 1.01)	−0.16	0.62 (0.50, 0.78)
Peak 25-min cadence	0.68 (0.57, 0.81)	0.67 (0.42, 1.07)	−0.15	0.61 (0.49, 0.76)
Peak 30-min cadence	0.68 (0.57, 0.81)	0.67 (0.42, 1.07)	−0.15	0.61 (0.49, 0.76)
Peak 60-min cadence	0.65 (0.55, 0.78)	0.72(0.46, 1.15)	−0.14	0.58 (0.46, 0.72)
Purposeful cadence	1.03 (0.86, 1.24)	1.00 (0.68, 1.48)	0.65	1.04 (0.79, 1.37)
Average daily cadence	0.77 (0.65, 0.91)	0.68 (0.44, 1.05)	0.69	0.62 (0.47, 0.81)

For better comparison, we standardized the cadence across stepping intensity metrics to rescale them into comparable range. The standardized cadence was calculated as ([exposure − mean]/standard deviation). Each metric was centered around a mean of 0 with a standard deviation of 1.

### Association of Stepping Intensity with Cancer Mortality

**FIGURE 5:**
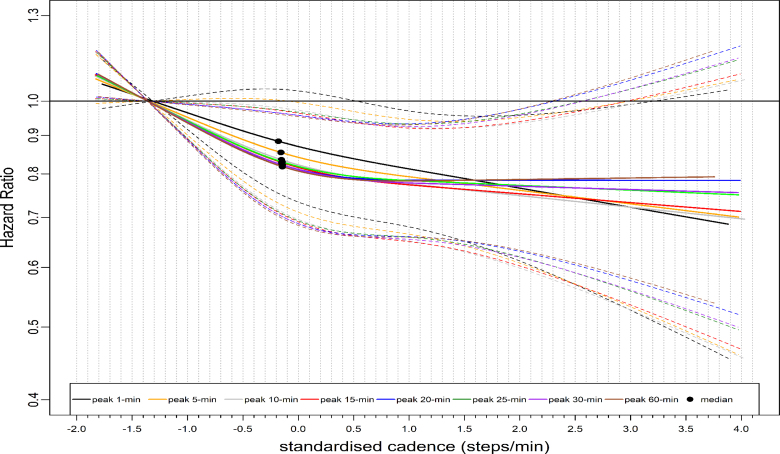
Dose-response association of standardized stepping intensity estimated across peak cadence metrics with cancer mortality. Total sample size is 65,253. the events for peak 1-min cadence are 1038; peak 5-min cadence, 1037; peak 10-min cadence, 1038; peak 15-min cadence,1038; peak 20-min cadence, 1039; peak 25-min cadence, 1037; peak 30-min cadence, 1038; peak 60-min cadence, 1042. The standardized cadence was calculated as ([exposure − mean]/standard deviation). The circle indicates the median steps per minute. We used Fine and Grey model to analyze the dose-response association and adjusted for age, sex, accelerometer wearing duration, average daily steps, smoking status, alcohol consumption, sleep duration, Townsend deprivation score, sedentary time, education levels, self-reported parental history of CVD and cancer, and self-reported medication use (cholesterol, blood pressure, and diabetes). The reference level is 5th percentile of each standardized peak cadence metric.

**FIGURE 6:**
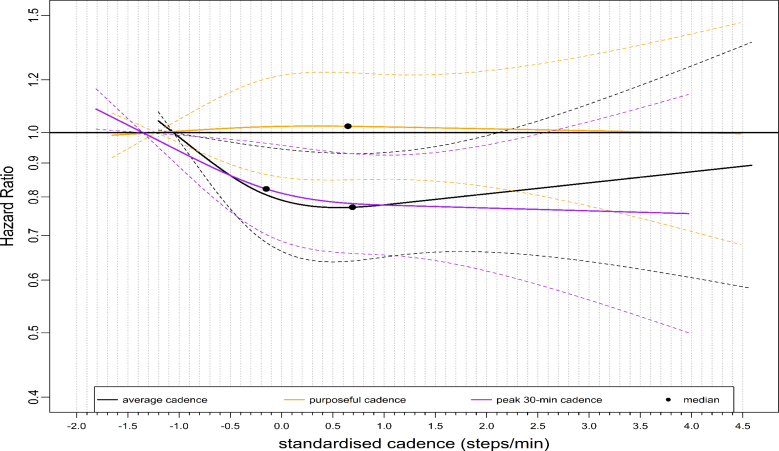
Dose-response association of standardized stepping intensity estimated by the average daily cadence, purposeful cadence, and peak 30-min cadence with cancer mortality. We compared two nonpeak cadence metrics and a representative peak cadence metric (peak 30-min cadence) in this figure. Total sample size is 65,253. The events for peak 30-min cadence are 1040; average daily cadence, 1048; purposeful cadence, 1214. The standardized cadence was calculated as ([exposure − mean]/standard deviation). The circle indicates the median standardized cadence. We used Fine and Grey model to analyze the dose-response association and adjusted for age, sex, accelerometer wearing duration, average daily steps, smoking status, alcohol consumption, sleep duration, Townsend deprivation score, sedentary time, education levels, self-reported parental history of CVD and cancer, and self-reported medication use (cholesterol, blood pressure, and diabetes). The reference level is 5th percentile of each standardized peak cadence metric.

We observed similar inverse linear dose-response associations for stepping intensity metrics and cancer mortality (Fig. 5). For example, the median point and the corresponding HR for peak 1- and 30-min cadence was −0.17 steps/min, 0.88 (95% CI: 0.75–1.03) and −0.15 steps/min, 0.82 (0.70–0.96), respectively (Supplemental Table 2, Supplemental Digital Content, http://links.lww.com/MSS/D319). At −0.5 standardized cadence at the lower stratum, the HR for peak 1- and 30-min cadence was 0.91 (95% CI: 0.80–1.03) and 0.86 (0.76–0.98), respectively. At 3.0 standardized steps/min at the upper stratum, the HR for peak 1- and 30-min cadence was 0.76 (95% CI: 0.57–1.01) and 0.76 (0.56–1.04), respectively (Supplemental Table 2, Supplemental Digital Content, http://links.lww.com/MSS/D319). The dose-response association for average daily cadence with cancer mortality revealed an inverse linear dose-response association (Fig. 6), which was similar to that of peak 30-min cadence. For example, the standardized median and the corresponding HR for average daily cadence was 0.69 steps/min, 0.77 (95% CI: 0.64–0.93), which is similar to that of peak 30-min cadence (Supplemental Table 2, Supplemental Digital Content, http://links.lww.com/MSS/D319). However, purposeful cadence did not reveal a dose-response association with cancer mortality (Fig. 6). For example, the standardized median and the corresponding HR for purposeful cadence were 0.65 steps/min, 1.02 (95% CI: 0.85–1.23) (Supplemental Table 2, Supplemental Digital Content, http://links.lww.com/MSS/D319).

### Association of Absolute Stepping Intensity with Mortality Outcomes

Supplemental Figures 4–9, Supplemental Digital Content, http://links.lww.com/MSS/D319, demonstrated the dose-response associations of stepping intensity estimated using cadence in absolute terms with mortality outcomes. All dose-response associations were similar to those using standardized cadence in terms of effect size. However, notable differences were observed in the absolute cadence values, with longer peak cadence intervals shifting the dose-response curves toward the lower end of the absolute cadence scale. For example, the median and HR for peak 60- and peak 1-min cadence were 55.1 steps/min, 0.65 (95% CI: 0.58–0.73) and 146.9 steps/min, 0.72 (0.63–0.81) for ACM (Supplemental Table 3, Supplemental Digital Content, http://links.lww.com/MSS/D319).

### Sensitivity Analysis

Using the normalized cadence scale (Supplemental Figs. 10–12, Supplemental Digital Content, http://links.lww.com/MSS/D319), weighted sample (Supplemental Figs. 13–15, Supplemental Digital Content, http://links.lww.com/MSS/D319), sex-specific subgroups (Supplemental Figs. 16–18, Supplemental Digital Content, http://links.lww.com/MSS/D319), and PA level-specific subgroups (Supplemental Figs. 19–21, Supplemental Digital Content, http://links.lww.com/MSS/D319) did not materially change the differences between stepping intensity metrics in their dose-response associations with mortality outcomes compared to the main analysis.

## DISCUSSION

Despite the vital role of intensity in PA guidelines, few studies have examined the dose-response association between stepping intensity and all-cause, CVD, and cancer mortality in free-living environments. Furthermore, inconsistent findings are reported among the few studies that have examined these relationships. A potential reason might be the inconsistency of the metrics estimating stepping intensity. To our knowledge, this study is the first to compare the dose-response associations of various stepping intensity estimation metrics with long-term health outcomes. All peak cadence metrics, along with average daily cadence, revealed similar beneficial dose-response associations with all-cause, CVD, and cancer mortality, respectively. Longer peak cadence metrics, particularly peak 60-min cadence, only revealed marginally lower mortality risk compared to shorter ones. This suggests that these stepping intensity estimation metrics may be used interchangeably. Additionally, this finding suggests that researchers may apply the metrics accordingly for different research questions. In contrast, we did not observe a dose-response association for purposeful cadence with mortality outcomes, indicating that this particular metric is not sensitive in detecting changes. When expressed in absolute terms, the selection of stepping intensity metric showed similar mortality gain across different metrics, aligning with the findings from the standardized analysis.

Our findings reveal that the choice of stepping intensity metric does not have an appreciable impact on dose-response associations with the health outcomes examined herein in real-world settings, strengthening researchers’ confidence in utilizing a wide range of stepping intensity metrics to describe ambulatory PA and associations with health outcomes. Future review studies can directly synthesize evidence from studies employing different peak or average cadence metrics. Collectively, our study facilitates the establishment and summarization of evidence on stepping intensity that can advise the public, policy makers and inform the step-based public health guidelines.

We observed a relatively similar dose-response association with each mortality outcome between long and short duration peak cadence metrics. Peak 1-min cadence was found to be linked with cardiorespiratory fitness (42)—a strong and consistent predictor for mortality and morbidity in a meta-analysis involving 20.9 million adults (43), suggesting that the peak cadence of even the shortest minute may reflect stepping intensity estimation and convey useful information on health improvement. Prior research has shown that peak 1-min cadence and peak 30-min cadence was all positively associated with key anthropometric and cardiometabolic health markers related to chronic diseases, such as age, body mass index, waist circumference, blood pressure, glucose, insulin, high-density lipoprotein cholesterol, triglycerides, and glycohemoglobin (15,44,45), showing the plausibility of peak cadence metrics, regardless of short or long metrics, linking with similar mortality risk. Taken together, it might explain the observed similarity among the short and long duration peak cadence metrics in terms of the association with mortality risk. Longer peak cadence metrics revealed marginally lower mortality risk compared to shorter ones. Longer peak cadence metrics (e.g., 30- or 60-min peaks) require sustained high-intensity stepping across the day, whereas achieving a single minute of high stepping intensity is relatively easy for most participants. This leads to lower variability in the magnitude of stepping intensity and in turn reduced discriminatory power for mortality risk.

The similar dose-response associations observed between peak and average cadence metrics provide researchers with the flexibility to select the appropriate metric for the specific research questions. For example, among the population groups with limited stepping time (46), metrics such as peak 60-min cadence may capture substantial number of minutes with near-zero or zero step counts, leading to peak 60-min cadence being unrepresentative of the actual stepping intensity. In such scenarios, shorter peak cadence metrics may provide a more accurate reflection of the actual stepping intensity. On the contrary, for adults who accumulate substantial stepping time per day, such as athletes, peak 60-min cadence or the average cadence might be more suitable for estimating their free-living stepping intensity.

Although a greater number of purposeful steps (i.e., ≥40 steps/min) per day was positively associated with various health outcomes for middle-aged and older adults (10,47), we did not observe dose-response association between purposeful cadence and mortality outcomes. The two components used to calculate purposeful cadence—purposeful stepping time and total number of purposeful steps—tend to vary together due to their high correlation. As a result, their ratio (i.e., purposeful cadence) exhibits limited variability compared to other stepping intensity metrics. This reduced variation may limit its ability to capture the additional mortality gain of higher stepping intensity at a finer level, as achieved by metrics such as peak 30-min cadence. Consequently, this might result in a lack of dose-response association with mortality risk.

Our study has several strengths. We compared a comprehensive set of stepping intensity estimation metrics based on a large prospective cohort with an average of roughly 8 yr of follow-up. In addition, we conducted four different sensitivity analyses to examine the robustness of the findings; one using an alternative scaling method (i.e., normalized scaling) to assess the robustness of the comparison method (i.e., standardized scaling) used in the main analysis; another improving representativeness by using sample weights; and two other analyses assessing the consistency of the findings within subgroups of the sample. This study also has some limitations. Wrist-worn accelerometer algorithms may be prone to step-detection error, registering steps from nonstep-related wrist movement (48), while also failing to register steps during ambulatory behavior when wrist movement is not pronounced (49). In addition, our examination of minute-level cadence-based metrics may not adequately reflect brief high-intensity stepping bursts (e.g., 30-s of running), which may display strong health benefits (39).

## CONCLUSIONS

In conclusion, peak cadence and average cadence metrics can be used interchangeably for estimating stepping intensity and relationships with health outcomes. Our finding also suggests that researchers can apply the metrics preferentially depending on their research question. Purposeful cadence may not be a suitable metric for estimating stepping intensity. The findings might address the issue of metric selection for stepping intensity in free-living environment, advancing the establishment of evidence base for health effects of stepping intensity.

This research was conducted using the UK Biobank Resource under Application Number 25813. The authors thank all the participants and professionals who contributed to the UK Biobank. This study was funded by the National Health and Medical Research Council (NHMRC) through a Leadership Level 2 Fellowship to Emmanuel Stamatakis (APP1194510). The funder had no specific role in any of the following study aspects: the design and conduct of the study; collection, management, analysis, and interpretation of the data; preparation, review, or approval of the manuscript; and the decision to submit the manuscript for publication. E. S. is a paid consultant and holds equity in Complement One, a US-based startup whose services relate to physical activity. All other authors disclose no conflict of interest for this work. The other authors declare that they have no conflicts of interests. E. S. and M. N. A. contributed to the concept. E. S., M. N. A., and L. W. contributed to the study design. M. N. A. contributed to data acquisition. L. W. performed data analyses and drafted the manuscript. All authors contributed to the data interpretation. All authors revised it and gave final approval and agreed to be accountable for all aspects of the work, ensuring integrity and accuracy. Our study utilized data from the UK Biobank, which has received ethical approval from the National Research Ethics Service (Ref 11/NW/0382). Informed consent was obtained from all individual participants included in the study. The data that support the findings of this study are available from the UK Biobank, but restrictions apply to the availability of these data, which were used under license for the current study, and so are not publicly available. Data are, however, available from the authors upon reasonable request and with the permission of the UK Biobank. Code will be available upon reasonable request to the corresponding author. The results of the study are presented clearly, honestly, and without fabrication, falsification, or inappropriate data manipulation. The results of the present study do not constitute endorsement by the American College of Sports Medicine.

## Supplementary Material
