# Supplementary material for: Does the Choice of Stepping Intensity Metric Influence Dose-Response Associations with Mortality? Analysis on UK Population Cohort Study of 65,253 Adults
Source: Med Sci Sports Exerc. 2025 Oct 27;58(3):506–16. doi: 10.1249/MSS.0000000000003887 (PMC12863595; doi:10.1249/MSS.0000000000003887)
Supplement: Supplementary file 1 [file msse-58-506-s001.pdf]

**Does the choice of stepping intensity metric influence dose-response associations with mortality? A UK population cohort study of 65,253 adults**

**Supplemental Document**


| Page | Item |
|---|---|
| 1 | **Supplementary Figure 1**: Flow diagram of participants in the study |
| 2 | **Supplementary Figure 2:** Distribution of the Standardised Peak Cadence Metrics |
| 3 | **Supplementary Figure 3:** Distribution of Standardised Non-peak Cadence Metrics |
| 4-15 | **Supplementary Figure 4-9**: Dose-Response Association of the Absolute Stepping Intensity Estimated across Metrics with All-Cause/CVD/Cancer Mortality |
| 16-21 | **Supplementary Figure 10-12:** Dose-Response Association of Normalised Stepping Intensity Estimated by Metrics with All-cause/CVD/Cancer Mortality |
| 22-27 | **Supplementary Figure 13-15:** Dose-Response Association of Stepping Intensity Estimated by Peak Cadence Metrics with All-cause/CVD/Cancer Mortality Using Weighted Sample |
| 28-36 | **Supplementary Figure 16-18**: Sex-specific Dose-response Association of Standardised Stepping Intensity Metrics with All-cause/CVD/cancer Mortality |
| 37-54 | **Supplementary Figure 19-21**: PA-level Specific Dose-response Association of Standardised Stepping Intensity Metrics with All-cause/CVD/Cancer Mortality |
| 55-59 | **Supplementary Table 1**: Baseline Characteristics of Study Participants by Quartiles of Peak 30-min Cadence Per Day |
| 60-61 | **Supplementary Table 2:** Hazard Ratio of Cancer Mortality at -0.5, 3.0 and Median Standardised Steps per Min Estimated by Peak Cadence and Non-Peak Cadence Metrics |
| 62-63 | **Supplementary Table 3** Hazard Ratio of All-Cause Mortality Associated with the Minimum, Median and Maximum Stepping Intensities (in absolute cadence value), Estimated by Peak Cadence and Non-Peak Cadence Metrics |
| 64-65 | **Supplementary Table 4** Hazard Ratio of CVD Mortality Associated with the Minimum, Median and Maximum Stepping Intensities (in absolute cadence value), Estimated by Peak Cadence and Non-Peak Cadence Metrics |
| 66-67 | **Supplementary Table 5** Hazard Ratio of Cancer Mortality Associated with the Minimum, Median and Maximum Stepping Intensities (in absolute cadence value), Estimated by Peak Cadence and Non-Peak Cadence Metrics |

| | |
|---|---|
| | |

**Supplemental Figure 1** Flow diagram of participants

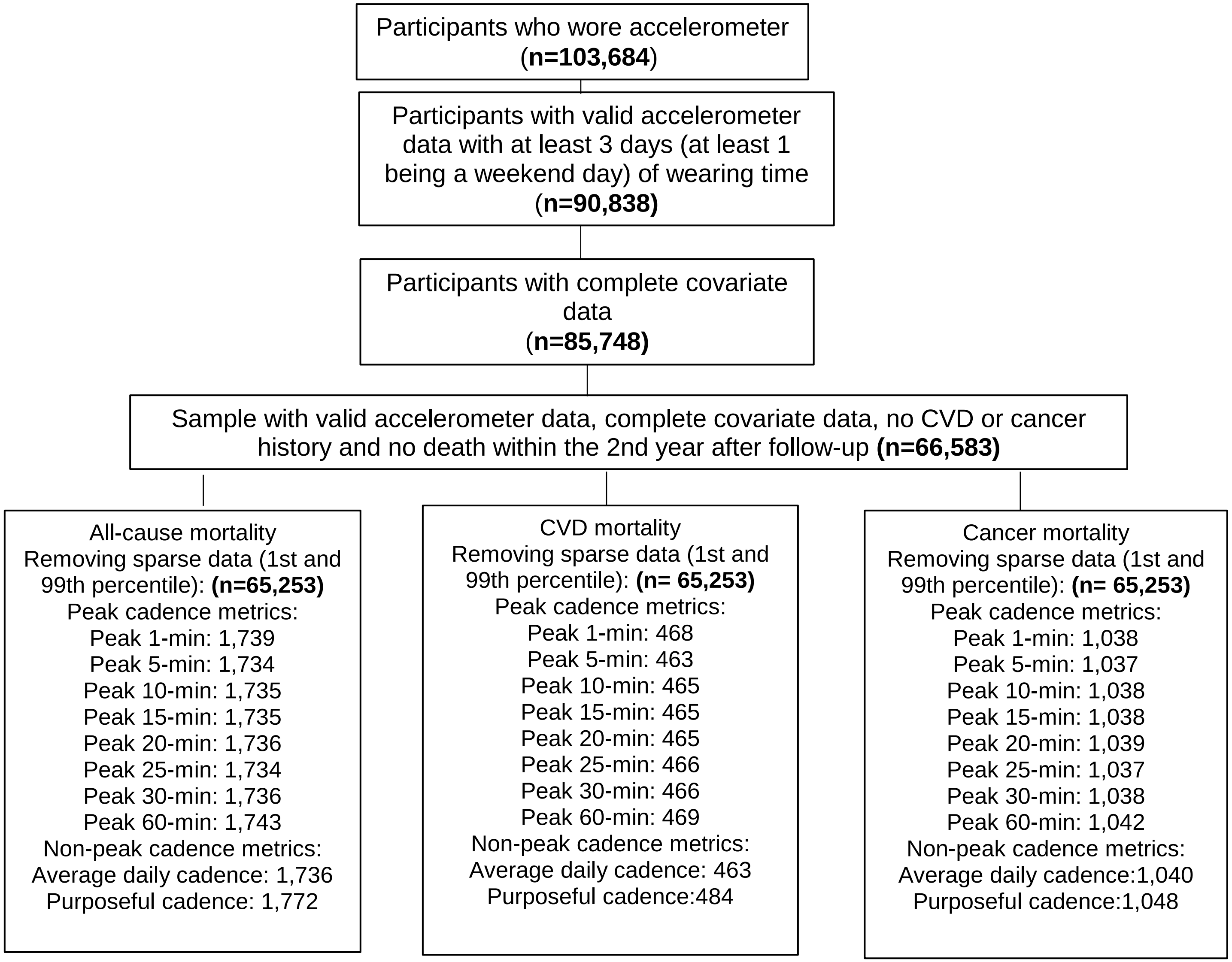

**Supplemental Figure 2** The Distribution of Standardised Peak Cadence Metrics

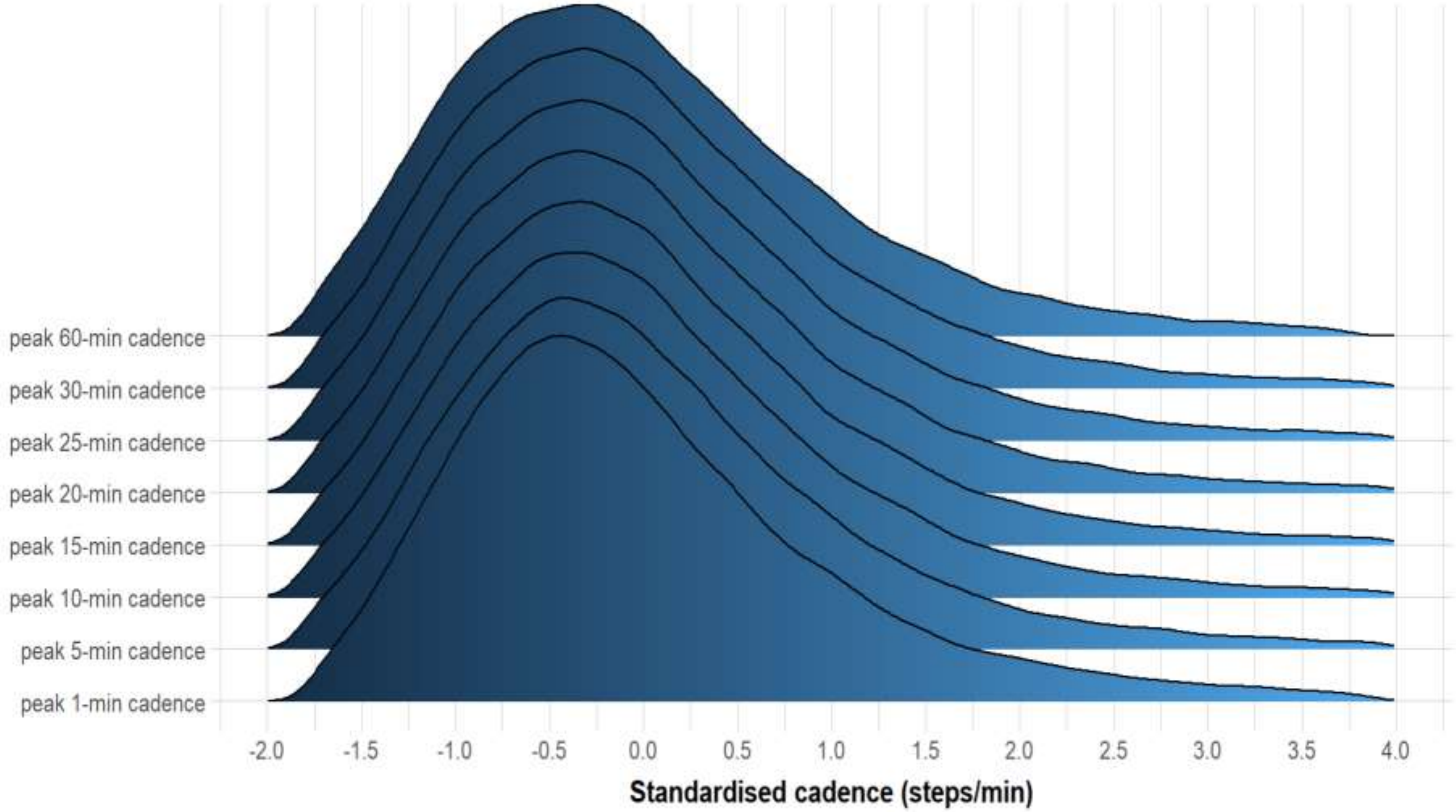

**Supplemental Figure 3** The Distribution of Standardised Non-peak Cadence Metrics and Peak 30-min Cadence

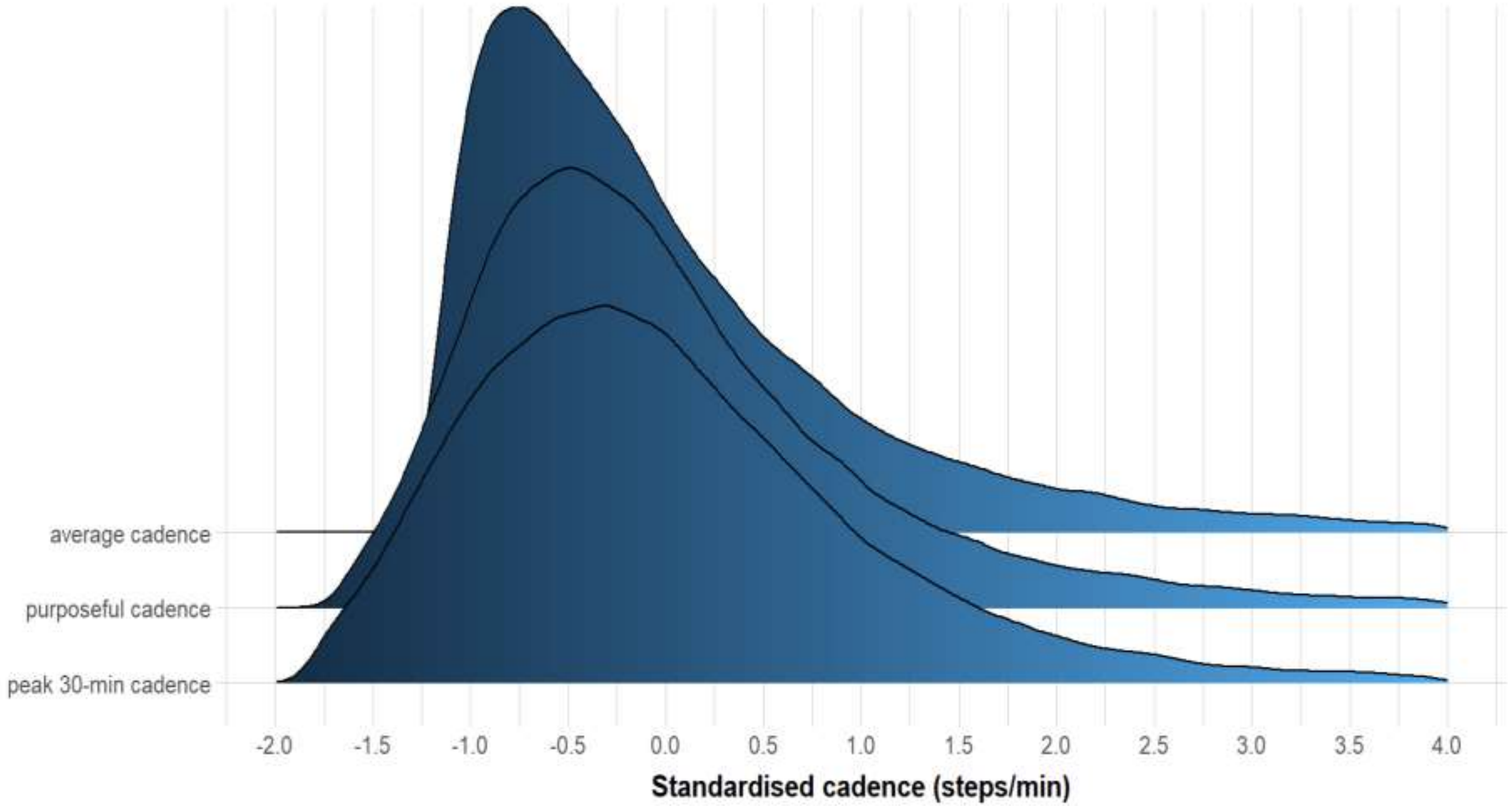

**Supplemental Figure 4** Dose-Response Association of Stepping Intensity Estimated across Peak Cadence Metrics with All-Cause Mortality

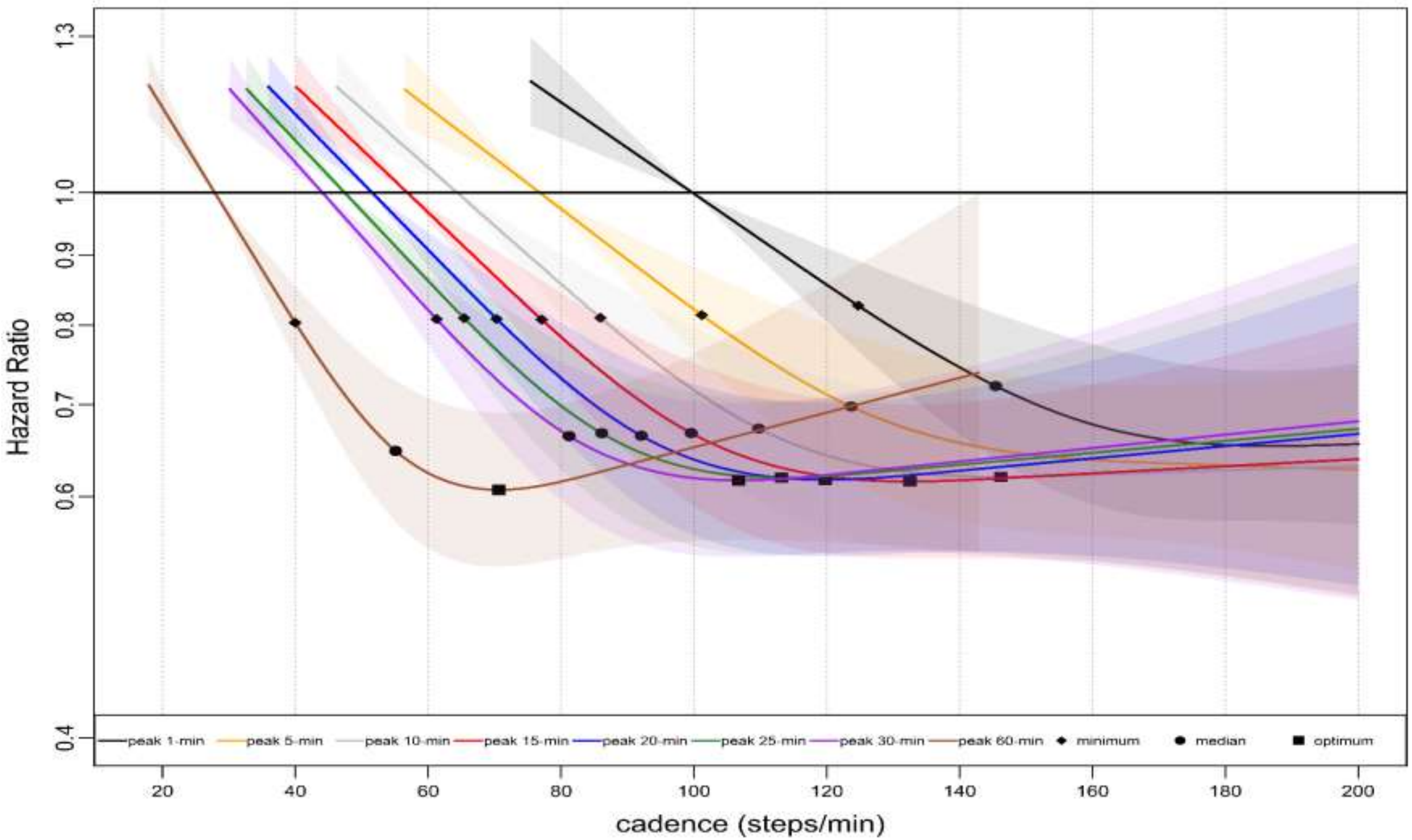

Total sample size is 65,253. The events for peak 1-min cadence is 1,739; peak 5-min cadence, 1,734; peak 10-min cadence, 1,735; peak 15-min cadence, 1,735; peak 20-min cadence, 1,736; peak 25-min cadence, 1,734; peak 30-min cadence, 1,736; peak 60-min cadence, 1,743. The diamond indicates the ED50 value i.e., minimum, the cadence associated with 50% of the optimal risk reduction; The circle indicates the median cadence. The square indicates the cadence that associated with optimal mortality risk reduction (Note: the square was not annotated if there was no nadir point). We truncated the data at 200 steps/min to reduce the risk of error data. The dose-response association was analysed using cox-regression model and adjusted for age, sex, accelerometer wearing duration, average daily steps, smoking status, alcohol consumption, sleep duration, townsend deprivation score, sedentary time, education levels, self-reported parental history of CVD and cancer, and self-reported medication use (cholesterol, blood pressure, and diabetes). The reference level is 5$^{th}$ percentile of each peak cadence metric.

**Supplemental Figure 5** Dose-Response Association of Stepping Intensity Estimated by the Average Daily Cadence, Purposeful Cadence and Peak 30-min Cadence with All-Cause Mortality

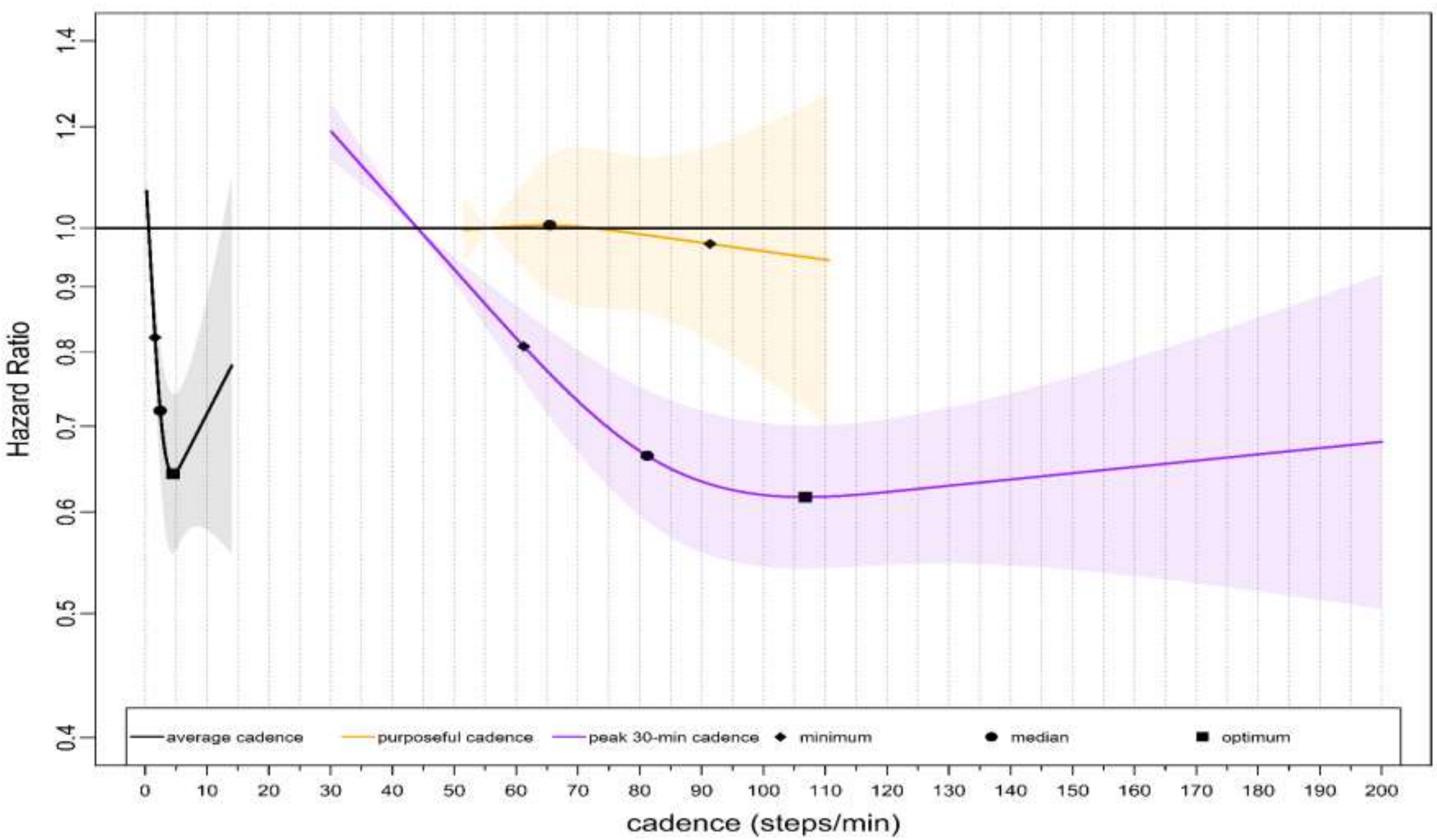

We compared two non-peak cadence metrics and a representative peak cadence metric (peak 30-min cadence) in this figure. Total sample size is 65,253. The events for peak 30-min cadence is 1,736; average daily cadence, 1,743; purposeful cadence, 1,772. The circle indicates the ED50 value i.e., minimum, the minimal steps per min associated with 50% of the optimal risk reduction; The triangle indicates the median cadence. **T**he diamond indicates the ED50 value i.e., minimum, the cadence associated with 50% of the optimal risk reduction; The circle indicates the median cadence. The square indicates the cadence that associated with optimal mortality risk reduction (Note: the square was not annotated if there was no nadir point). The dose-response association was analysed using cox-regression model and adjusted for age, sex, accelerometer wearing duration, average daily steps, smoking status, alcohol consumption, sleep duration, townsend deprivation score, sedentary time, education levels, self-reported parental history of CVD and cancer, and self-reported medication use (cholesterol, blood pressure, and diabetes). The reference level is 5$^{th}$ percentile of each metric.

**Supplemental Figure 6** Dose-Response Association of Stepping Intensity Estimated across Peak Cadence Metrics with CVD Mortality

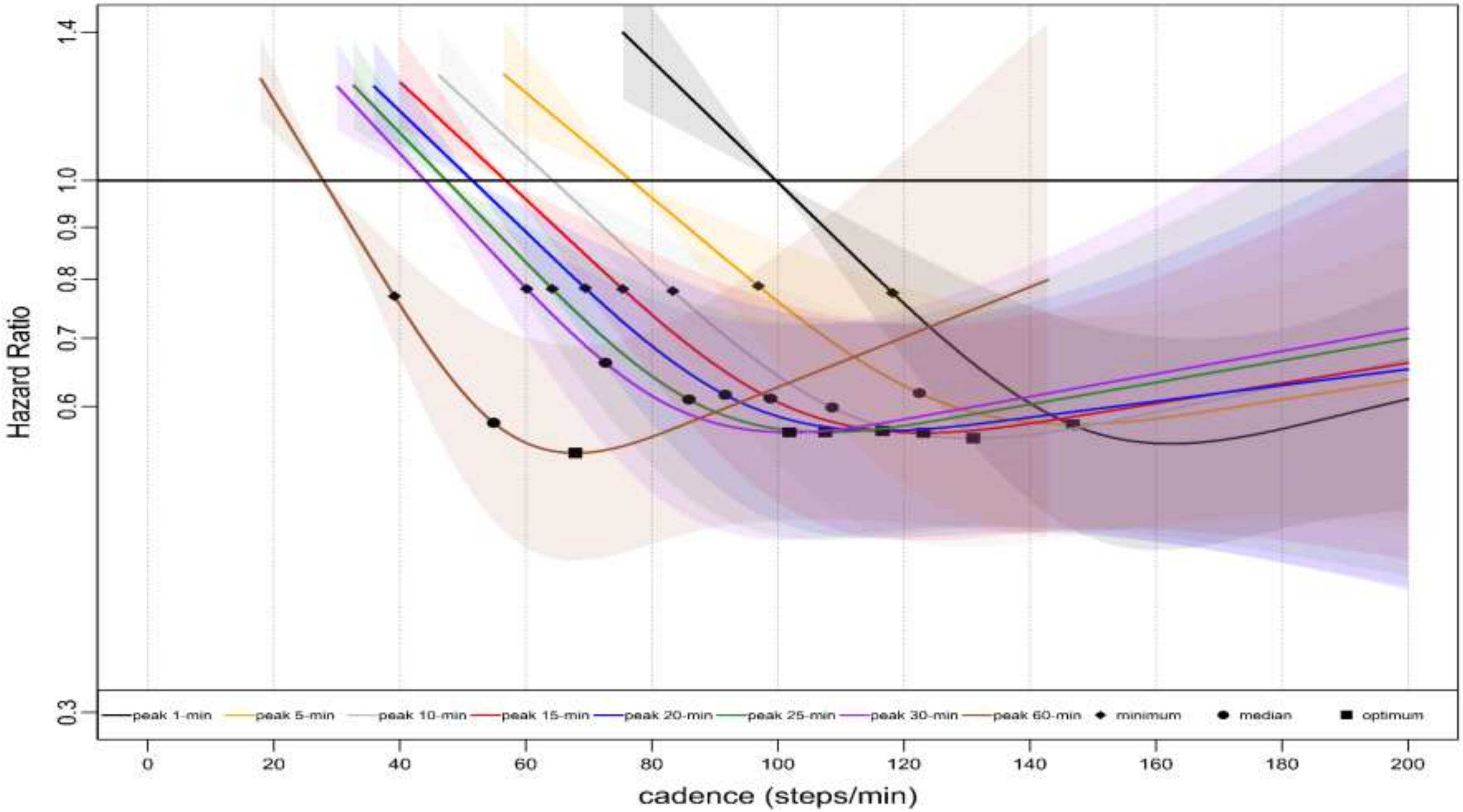

Total sample size is 65,253. The events for peak 1-min cadence is 468; peak 5-min cadence, 463; peak 10-min cadence, 465; peak 15-min cadence, 465; peak 20-min cadence, 465; peak 25-min cadence, 466; peak 30-min cadence, 466; peak 60-min cadence, 469. The diamond indicates the ED50 value i.e., minimum, the cadence associated with 50% of the optimal risk reduction; The circle indicates the median cadence. The square indicates the cadence that associated with optimal mortality risk reduction (Note: the square was not annotated if there was no nadir point). We truncated the data at 200 steps/min to reduce the risk of error data. We analysed the dose-response association using Fine and Grey model and adjusted for age, sex, accelerometer wearing duration, average daily steps, smoking status, alcohol consumption, sleep duration, Townsend deprivation score, sedentary time, education levels, self-reported parental history of CVD and cancer, and self-reported medication use (cholesterol, blood pressure, and diabetes). The reference level is 5$^{th}$ percentile of the distribution of each exposure.

**Supplemental Figure 7** Dose-Response Association of Stepping Intensity Estimated by Average Daily Cadence, Purposeful Cadence and Peak 30-min Cadence with CVD mortality

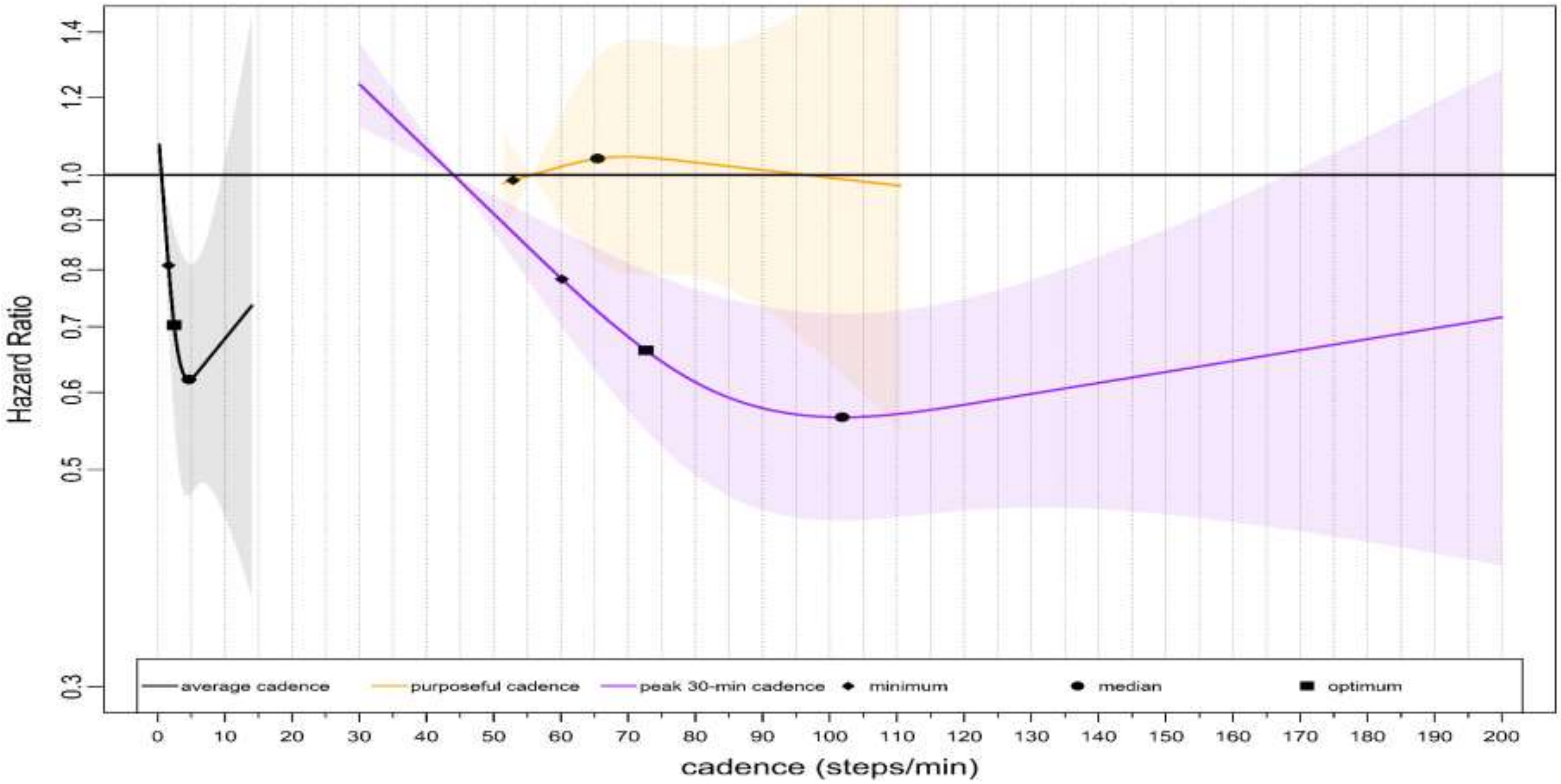

We compared two non-peak cadence metrics and a representative peak cadence metric (peak 30-min cadence) in this figure. Total sample size is 65,253, the events for peak 30-min cadence is 466; average daily cadence, 463; purposeful cadence, 484. The circle indicates the ED50 value i.e., minimum, the minimal steps per min associated with 50% of the optimal risk reduction; The triangle indicates the median cadence. The diamond indicates the ED50 value i.e., minimum, the cadence associated with 50% of the optimal risk reduction; The circle indicates the median cadence. The square indicates the cadence that associated with optimal mortality risk reduction (Note: the square was not annotated if there was no nadir point). We analysed the dose-response association using Fine and Grey model and adjusted for age, sex, accelerometer wearing duration, average daily steps, smoking status, alcohol consumption, sleep duration, Townsend deprivation score, sedentary time, education levels, self-reported parental history of CVD and cancer, and self-reported medication use (cholesterol, blood pressure, and diabetes). The reference level is 5$^{th}$ percentile of the distribution of each exposure.

**Supplemental Figure 8** Dose-Response Association of Standardised Stepping Intensity Estimated across Peak Cadence Metrics with Cancer Mortality

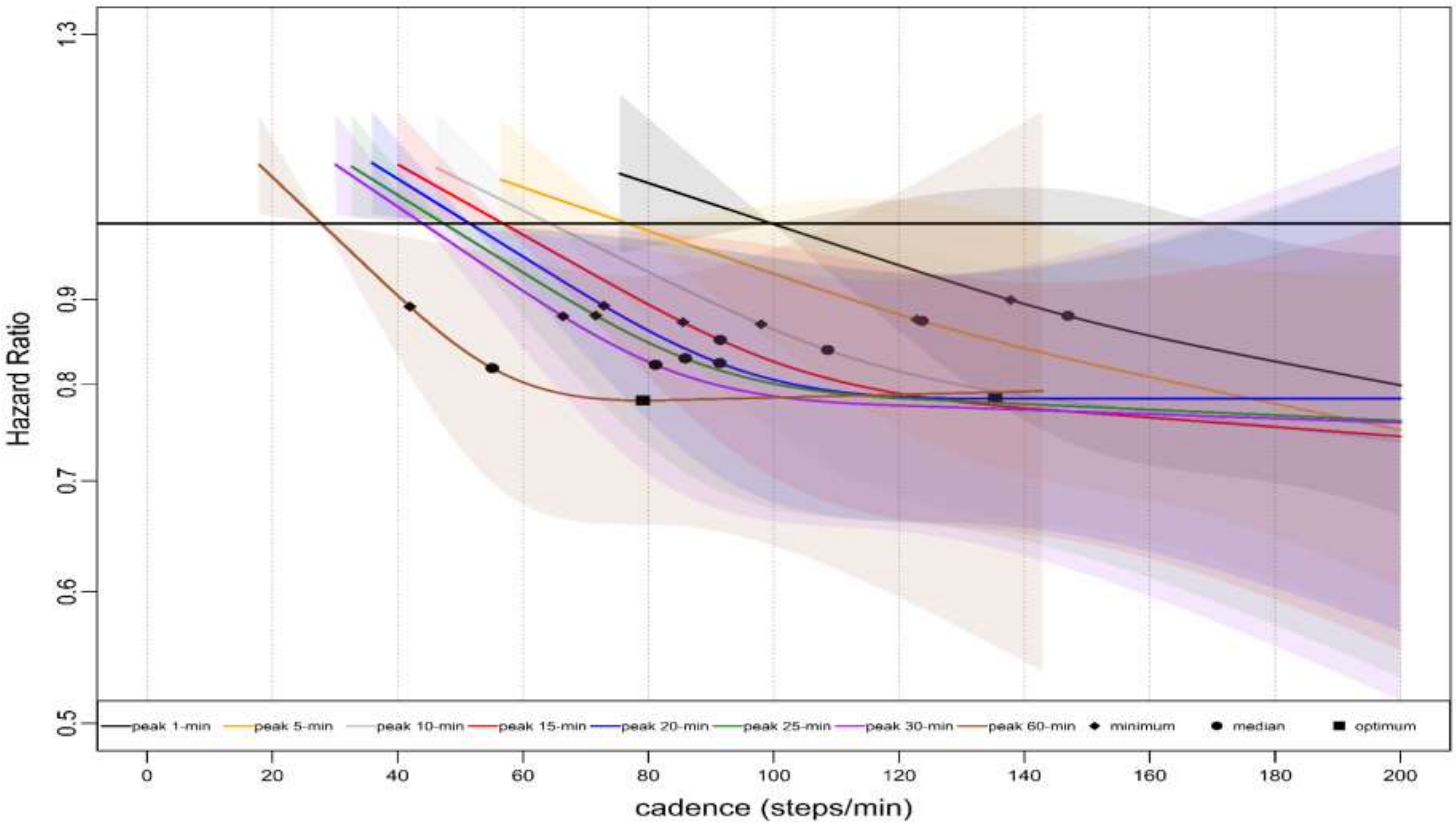

Total sample size is 65,253. the events for peak 1-min cadence is 1,038; peak 5-min cadence, 1,037; peak 10-min cadence, 1,038; peak 15-min cadence,1,038; peak 20-min cadence, 1,039; peak 25-min cadence, 1,037; peak 30-min cadence, 1,038; peak 60-min cadence, 1,042. The circle indicates the ED50 value i.e., minimum, the minimal steps per min associated with 50% of the optimal risk reduction; The triangle indicates the median steps per min. The diamond indicates the ED50 value i.e., minimum, the cadence associated with 50% of the optimal risk reduction; The circle indicates the median cadence. The square indicates the cadence that associated with optimal mortality risk reduction (Note: the square was not annotated if there was no nadir point). We truncated the data at 200 steps/min to reduce the risk of error data. We analysed the dose-response association using Fine and Grey model and adjusted for age, sex, accelerometer wearing duration, average daily steps, smoking status, alcohol consumption, sleep duration, Townsend deprivation score, sedentary time, education levels, self-reported parental history of CVD and cancer, and self-reported medication use (cholesterol, blood pressure, and diabetes). The reference level is 5$^{th}$ percentile of the distribution of each exposure.

**Supplemental Figure 9** Dose-Response Association of Stepping Intensity Estimated by Average Cadence, Purposeful Cadence and Peak 30-

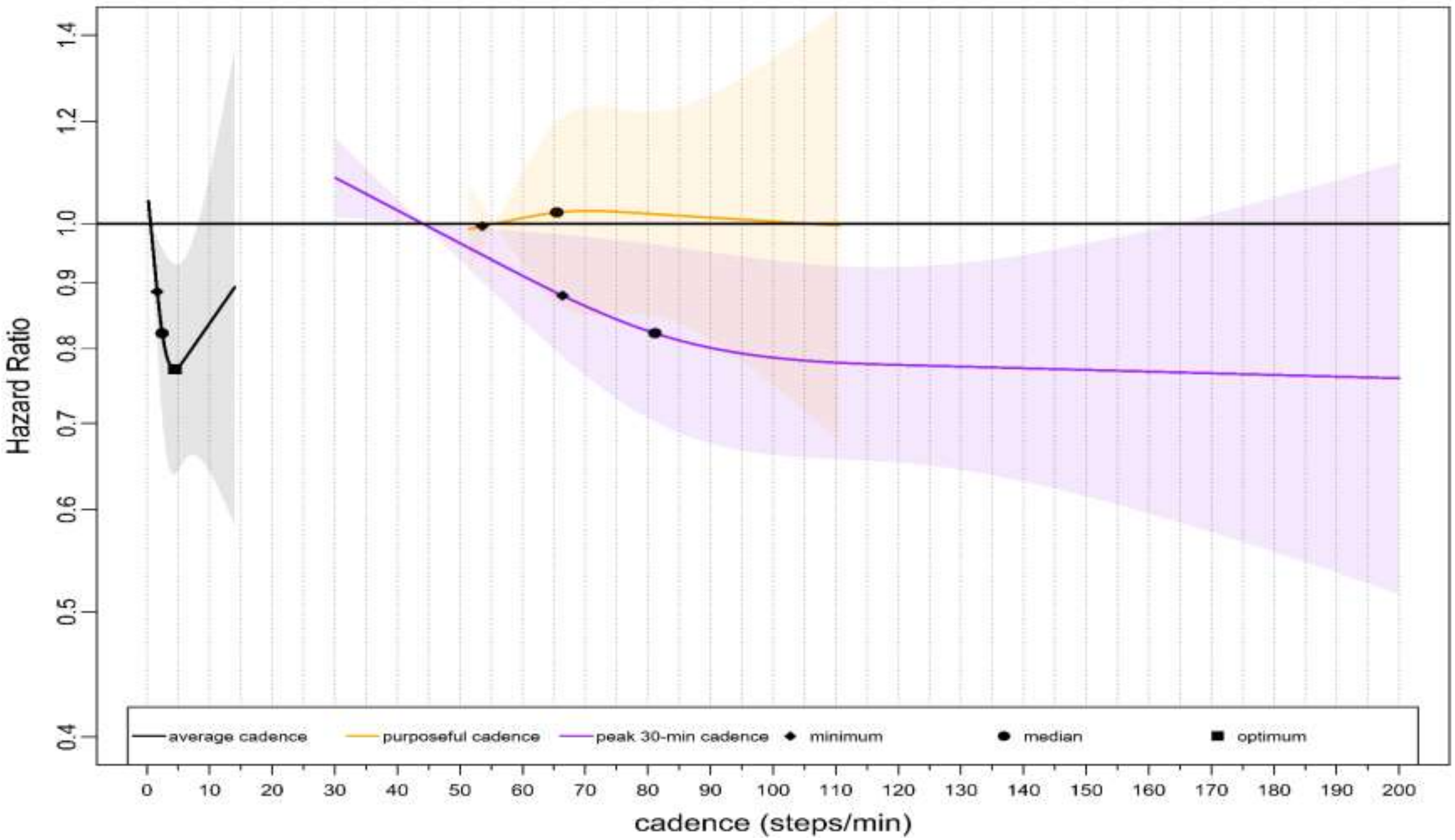

We compared two non-peak cadence metrics and a representative peak cadence metric (peak 30-min cadence) in this figure. Total sample size is 65,253. The events for peak 30-min cadence is 1,040; average daily cadence, 1,048; purposeful cadence, 1,214. The circle indicates the ED50 value i.e., minimum, the minimal cadence associated with 50% of the optimal risk reduction; The triangle indicates the median cadence. The diamond indicates the ED50 value i.e., minimum, the cadence associated with 50% of the optimal risk reduction; The circle indicates the median cadence. The square indicates the cadence that associated with optimal mortality risk reduction (Note: the square was not annotated if there was no nadir point). We analysed the dose-response association using Fine and Grey model and adjusted for age, sex, accelerometer wearing duration, average daily steps, smoking status, alcohol consumption, sleep duration, Townsend deprivation score, sedentary time, education levels, self-reported parental history of CVD and cancer, and self-reported medication use (cholesterol, blood pressure, and diabetes). The reference level is $5^{th}$ percentile of the distribution of each exposure.

**Supplemental Figure 10** Dose-Response Association of Normalised Stepping Intensity Estimated by Peak Cadence and Non-peak cadence Metrics with All-cause Mortality

**a) Peak cadence metrics**

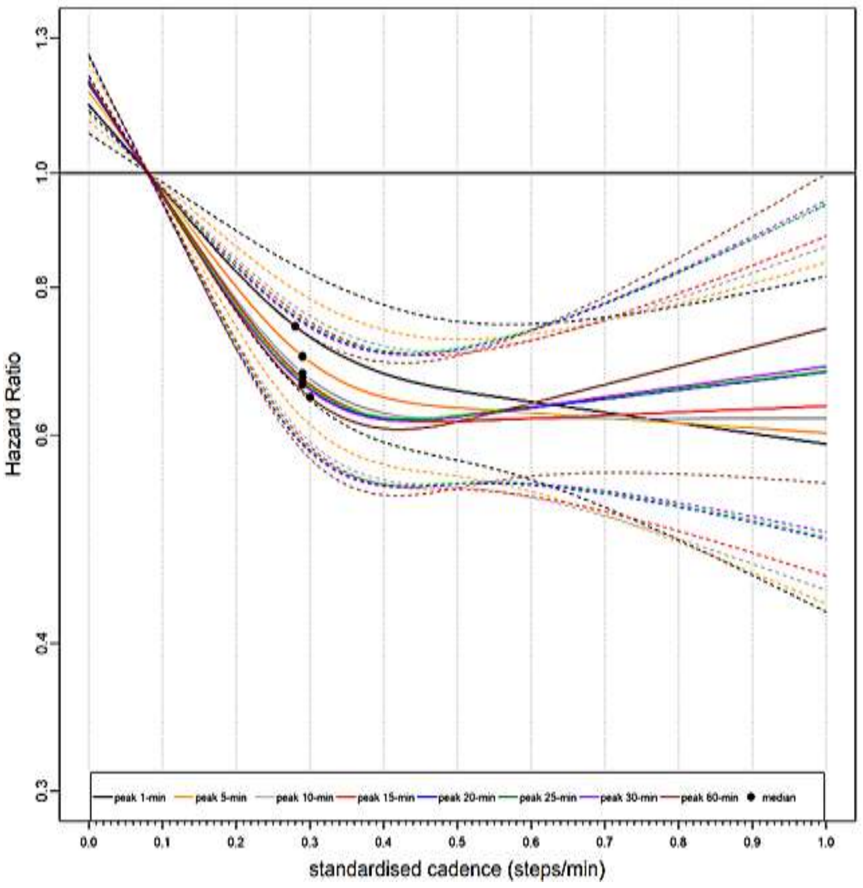


**b) non-peak cadence metrics**

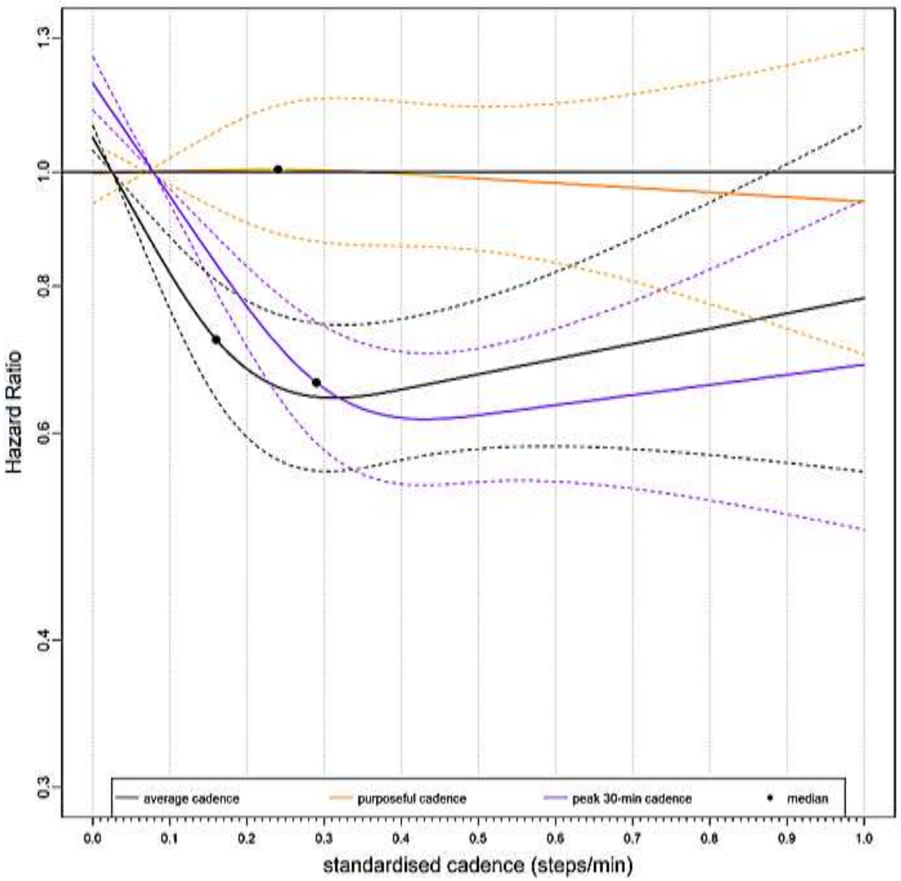

The circle indicates the median cadence. The dose-response association was analysed using cox-regression model and adjusted for age, sex, accelerometer wearing duration, average daily steps, smoking status, alcohol consumption, sleep duration, townsend deprivation score, sedentary time, education levels, self-reported parental history of CVD and cancer, and self-reported medication use (cholesterol, blood pressure, and diabetes). The reference level is 5th percentile of each peak cadence metric.

**Supplemental Figure 11** Dose-Response Association of Normalised Stepping Intensity Estimated by Peak Cadence Metrics with CVD Mortality

**a) Peak cadence metrics** **b) non-peak cadence metrics**

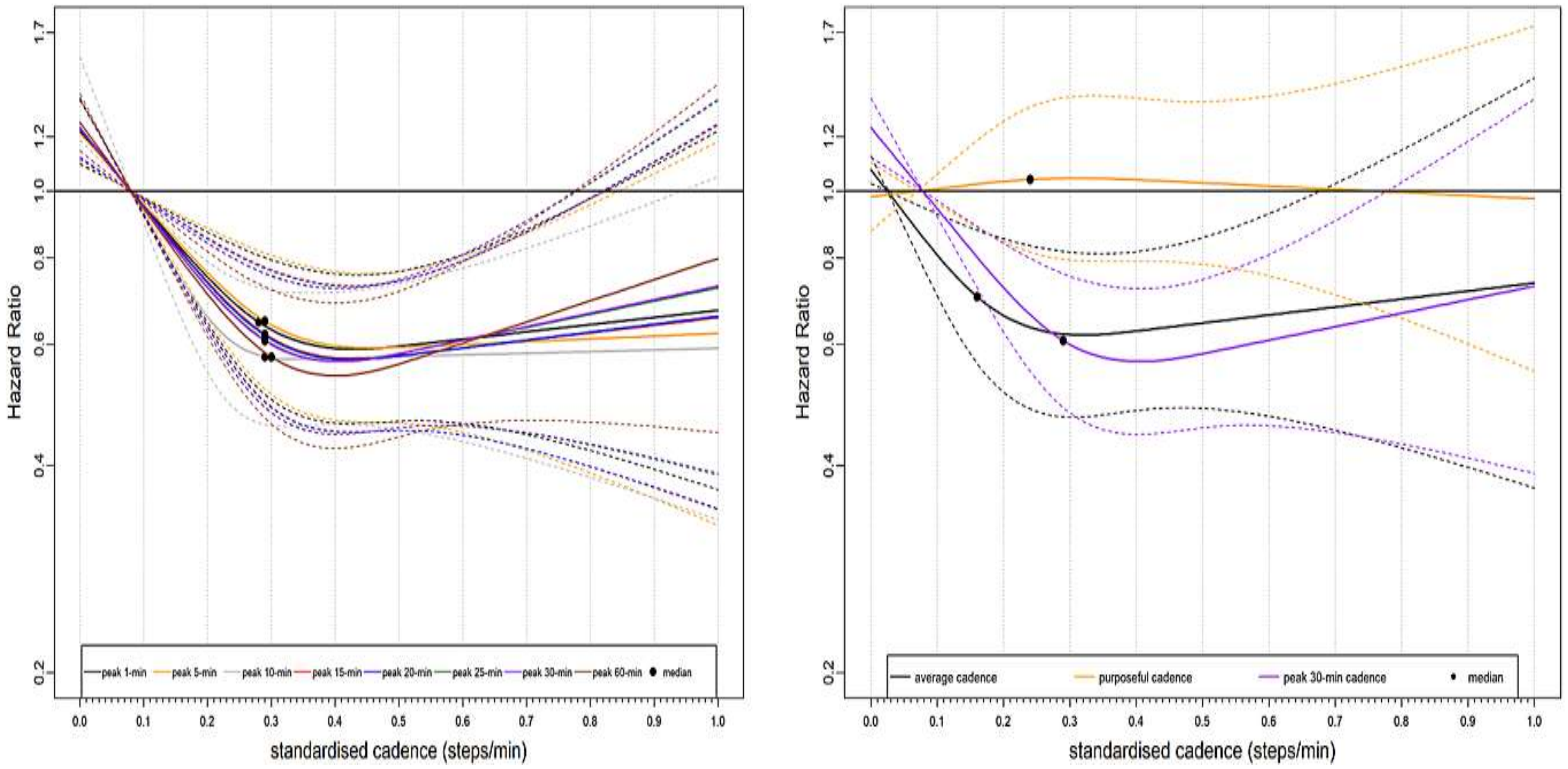

The circle indicates the median cadence. We analysed the dose-response association using Fine and Grey model and adjusted for age, sex, accelerometer wearing duration, average daily steps, smoking status, alcohol consumption, sleep duration, Townsend deprivation score, sedentary time, education levels, self-reported parental history of CVD and cancer, and self-reported medication use (cholesterol, blood pressure, and diabetes). The reference level is 5th percentile of the distribution of each exposure.

**Supplemental Figure 12** Dose-Response Association of Normalised Stepping Intensity Estimated by Peak Cadence Metrics with Cancer Mortality

**a) Peak cadence metrics**

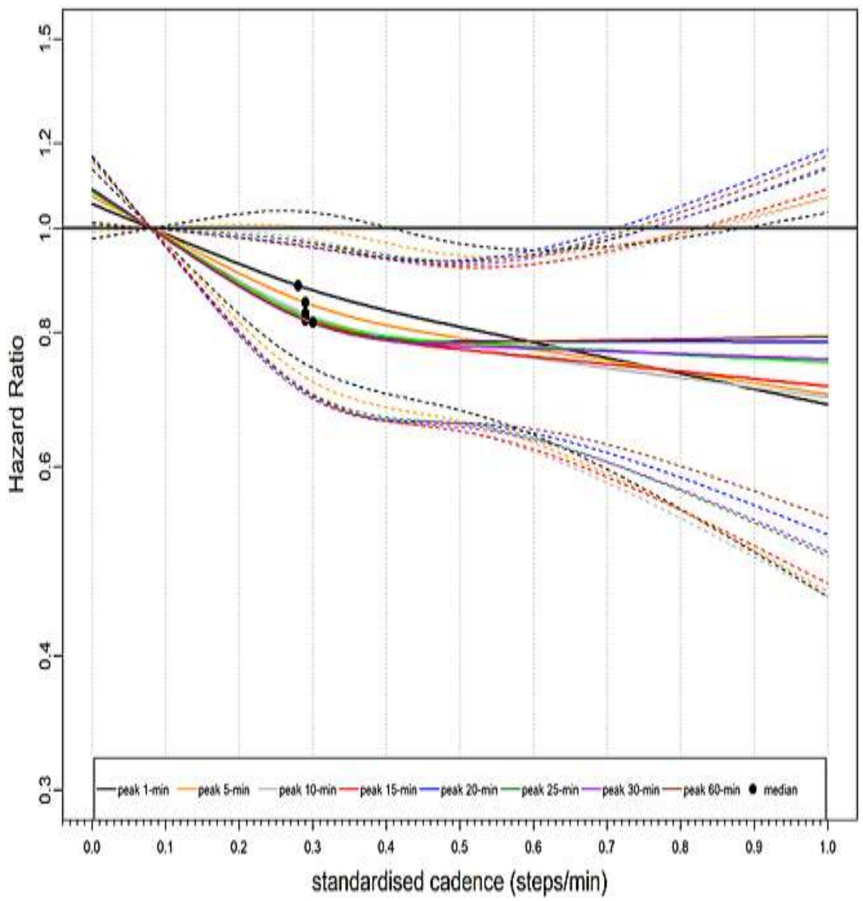


**b) non-peak cadence metrics**

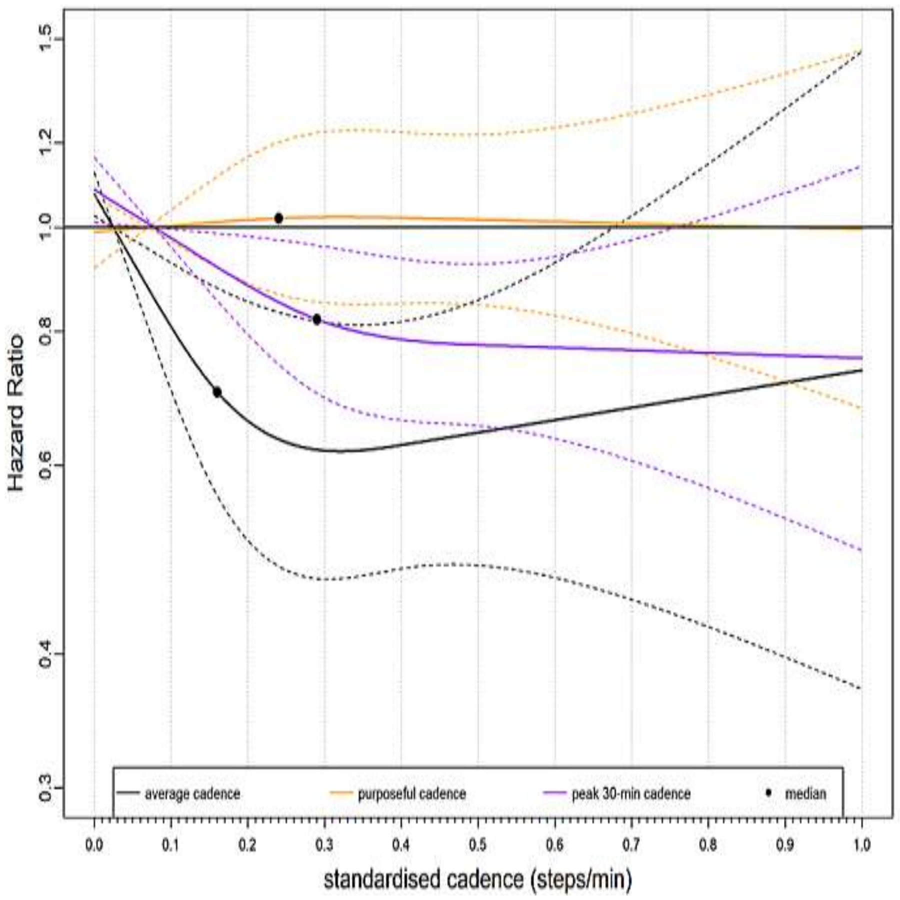

The circle indicates the median cadence. We analysed the dose-response association using Fine and Grey model and adjusted for age, sex, accelerometer wearing duration, average daily steps, smoking status, alcohol consumption, sleep duration, Townsend deprivation score, sedentary time, education levels, self-reported parental history of CVD and cancer, and self-reported medication use (cholesterol, blood pressure, and diabetes). The reference level is 5th percentile of the distribution of each exposure.

**Supplemental Figure 13** Dose-Response Association of Stepping Intensity Estimated by Peak and Non-peak Cadence Metrics with ACM Mortality Using Weighted Sample

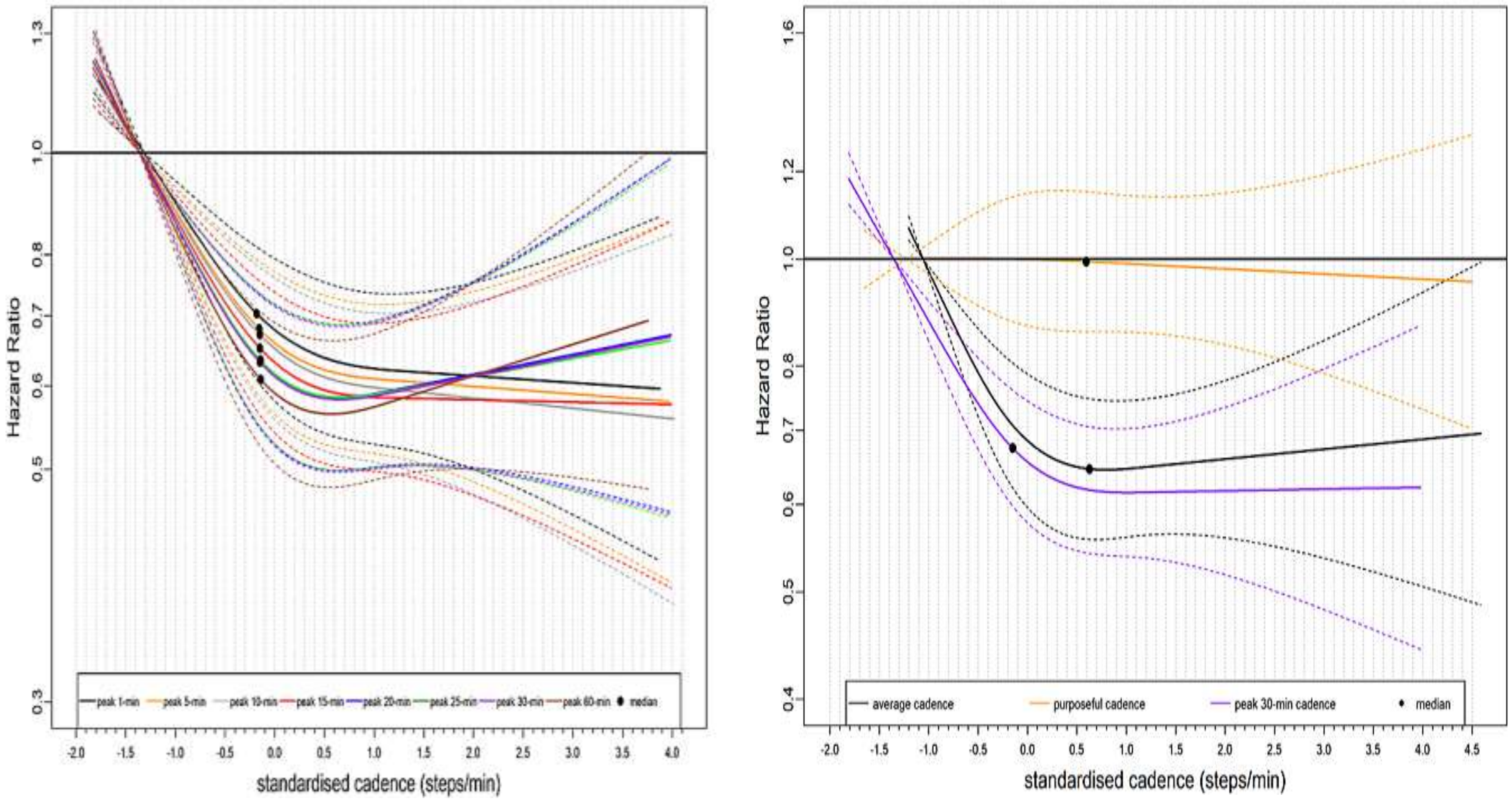

The circle indicates the median cadence. The dose-response association was analysed using cox-regression model and adjusted for age, sex, accelerometer wearing duration, average daily steps, smoking status, alcohol consumption, sleep duration, townsend deprivation score, sedentary time, education levels, self-reported parental history of CVD and cancer, and self-reported medication use (cholesterol, blood pressure, and diabetes). The reference level is 5th percentile of each peak cadence metric.

**Supplemental Figure 14** Dose-Response Association of Stepping Intensity Estimated by Peak Cadence Metrics with CVD Mortality Using Weighted Sample

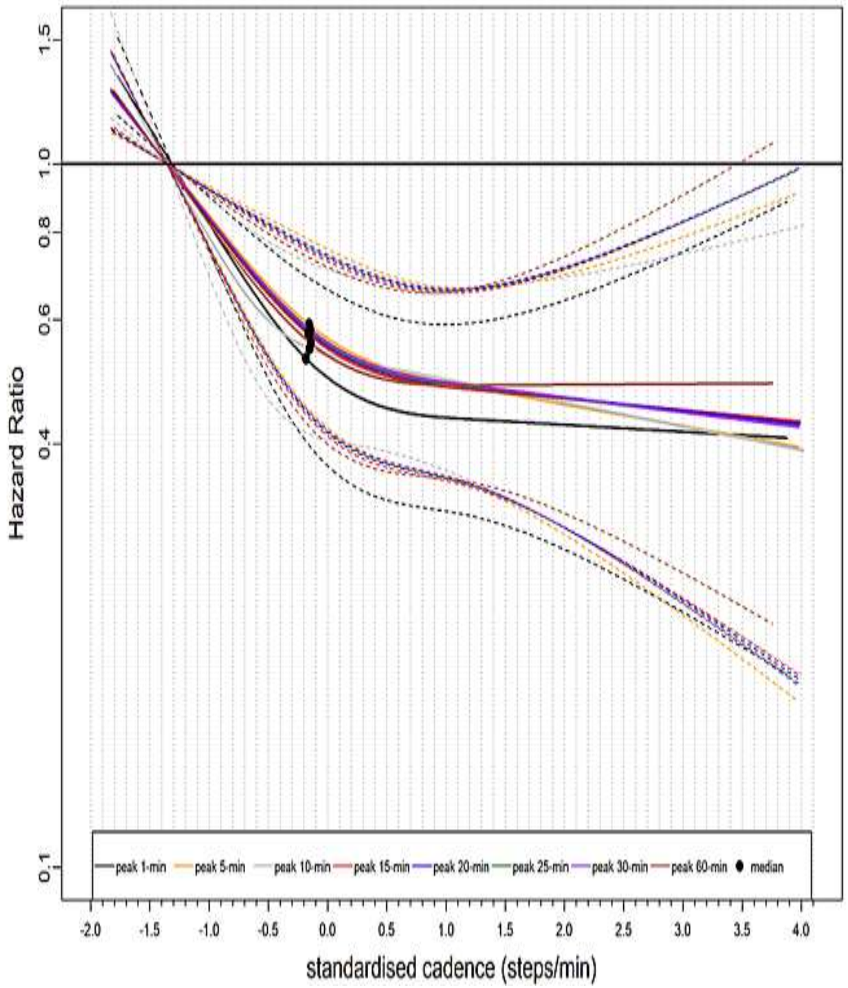


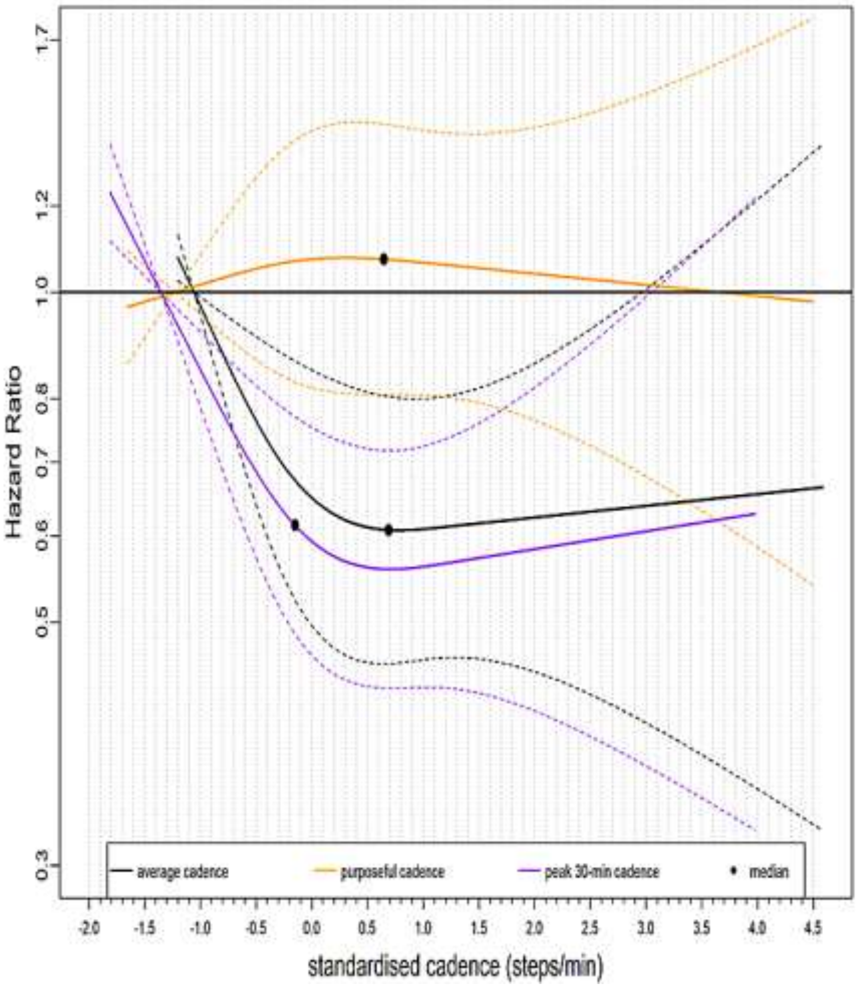

The circle indicates the median cadence. We analysed the dose-response association using Fine and Grey model and adjusted for age, sex, accelerometer wearing duration, average daily steps, smoking status, alcohol consumption, sleep duration, Townsend deprivation score, sedentary time, education levels, self-reported parental history of CVD and cancer, and self-reported medication use (cholesterol, blood pressure, and diabetes). The reference level is 5$^{th}$ percentile of the distribution of each exposure.

**Supplemental Figure 15** Dose-Response Association of Stepping Intensity Estimated by Peak and Non-peak Cadence Metrics with Cancer Mortality Using Weighted Sample

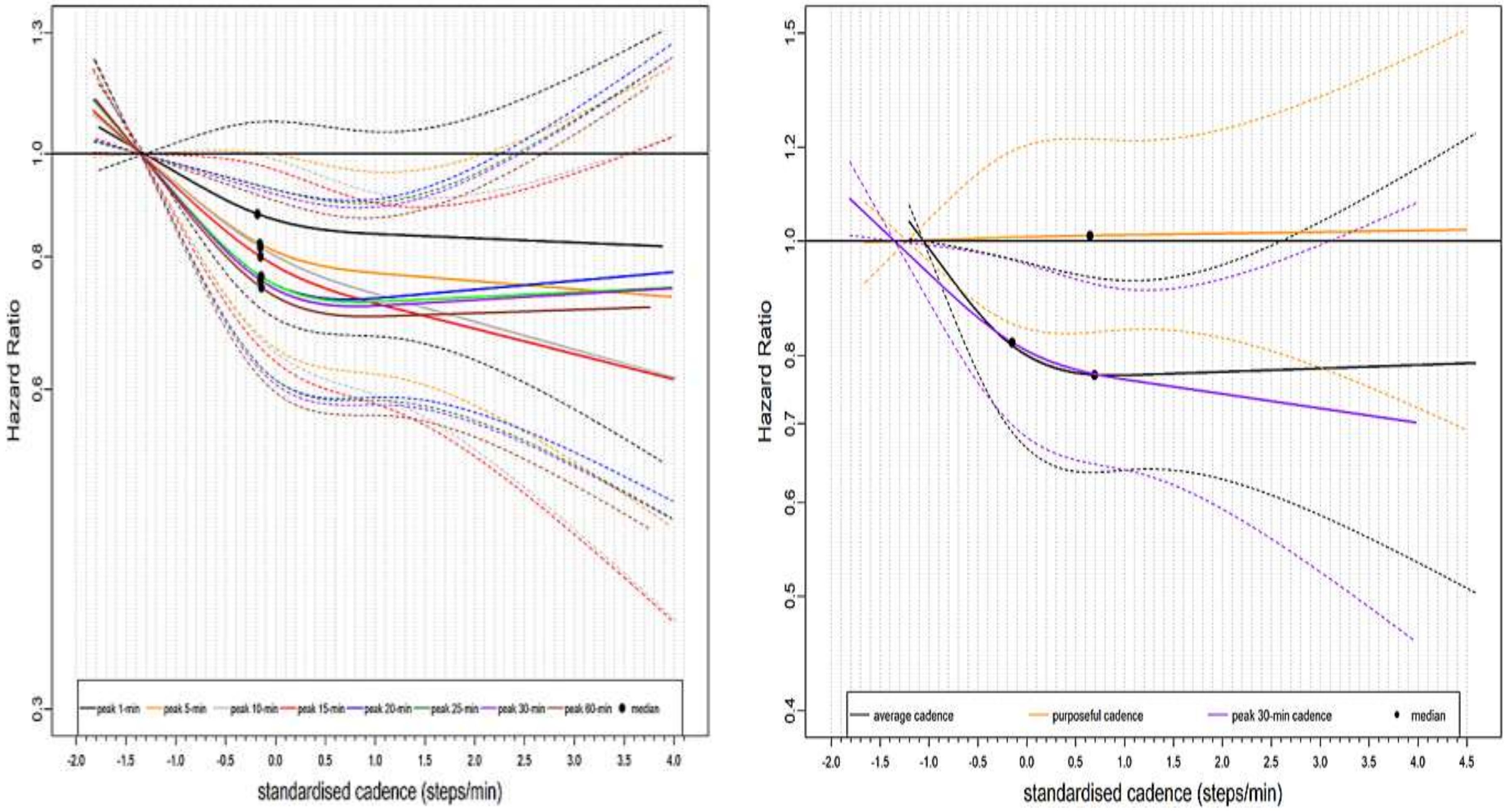

The circle indicates the median cadence. We analysed the dose-response association using Fine and Grey model and adjusted for age, sex, accelerometer wearing duration, average daily steps, smoking status, alcohol consumption, sleep duration, Townsend deprivation score, sedentary time, education levels, self-reported parental history of CVD and cancer, and self-reported medication use (cholesterol, blood pressure, and diabetes). The reference level is 5th percentile of the distribution of each exposure.

**Supplemental Figure 16** Sex-specific Dose-response Association of Standardised Stepping Intensity Estimated by Peak and Non-peak Cadence Metrics with All-cause Mortality

**a) Female**

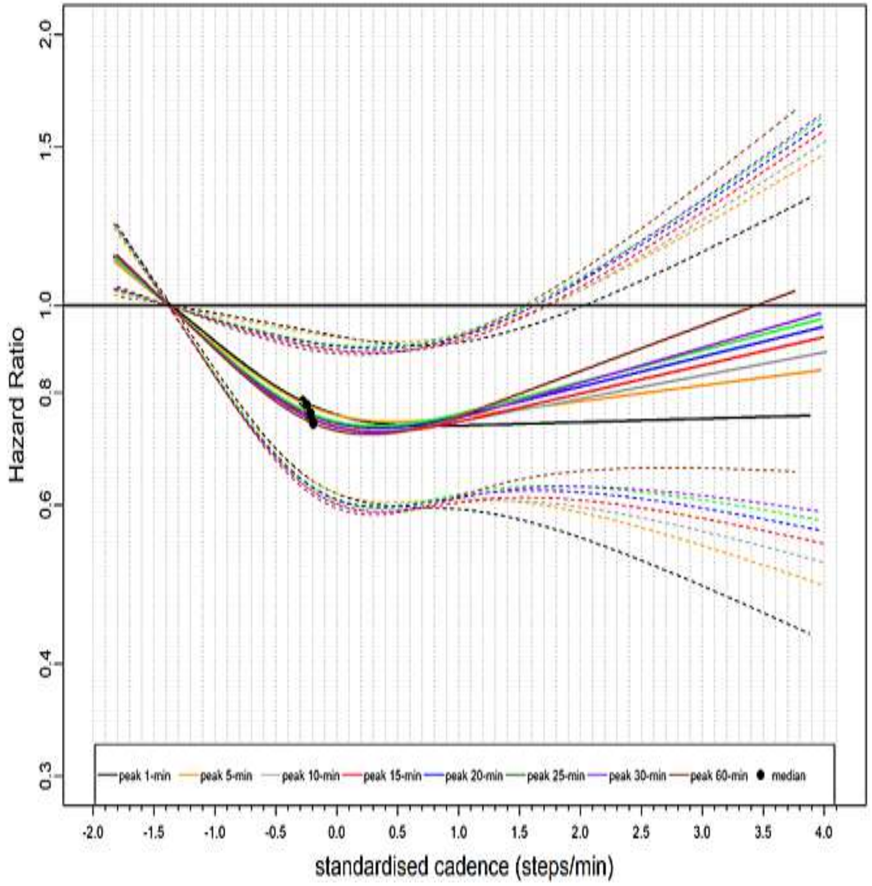


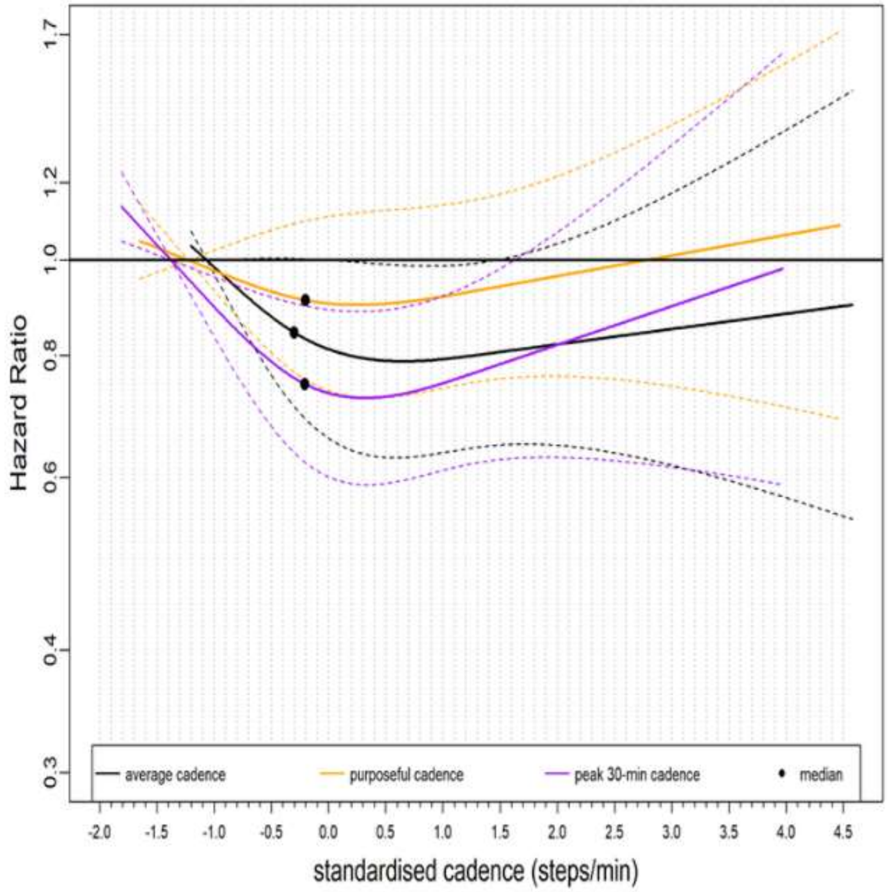

**b) Male**

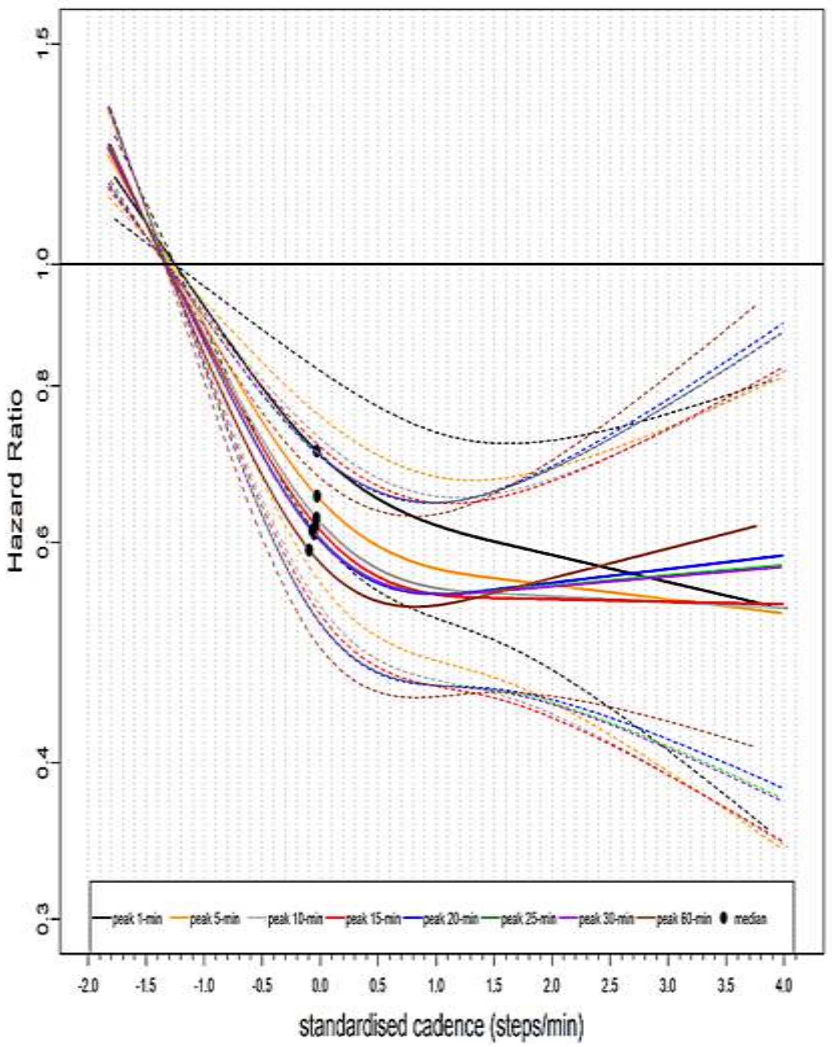
Hazard Ratio
1.5
1.0
0.8
0.6
0.4
0.3
peak 1-min
peak 5-min
peak 10-min
peak 15-min
peak 20-min
peak 25-min
peak 30-min
peak 60-min
median
-2.0
-1.5
-1.0
-0.5
0.0
0.5
1.0
1.5
2.0
2.5
3.0
3.5
4.0
standardised cadence (steps/min)

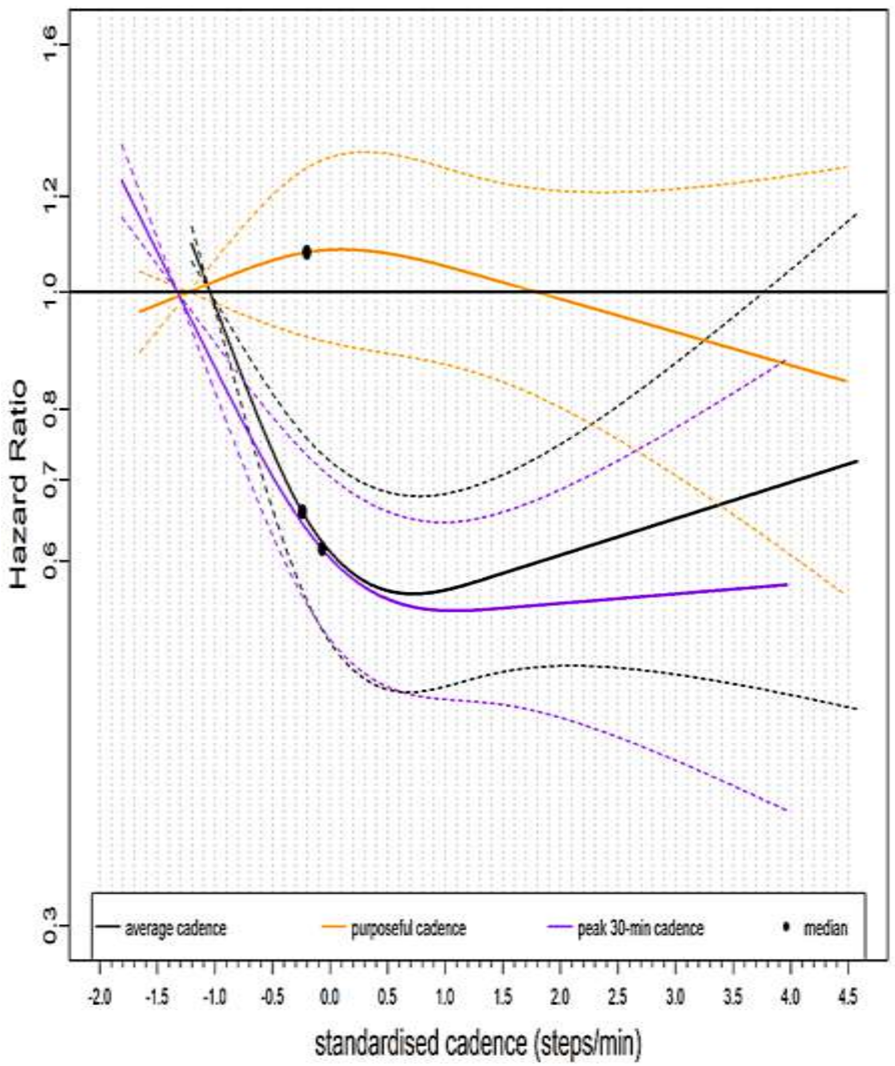
Hazard Ratio
1.6
1.2
1.0
0.8
0.7
0.6
0.3
average cadence
purposeful cadence
peak 30-min cadence
median
-2.0
-1.5
-1.0
-0.5
0.0
0.5
1.0
1.5
2.0
2.5
3.0
3.5
4.0
4.5
standardised cadence (steps/min)

The circle indicates the median cadence. The dose-response association was analysed using cox-regression model and adjusted for age, sex, accelerometer wearing duration, average daily steps, smoking status, alcohol consumption, sleep duration, townsend deprivation score, sedentary time, education levels, self-reported parental history of CVD and cancer, and self-reported medication use (cholesterol, blood pressure, and diabetes). The reference level is 5th percentile of each peak cadence metric.

**Supplemental Figure 17** Sex-specific Dose-response Association of Standardised Stepping Intensity Estimated by Peak Cadence Metrics with CVD Mortality

a) **Female**

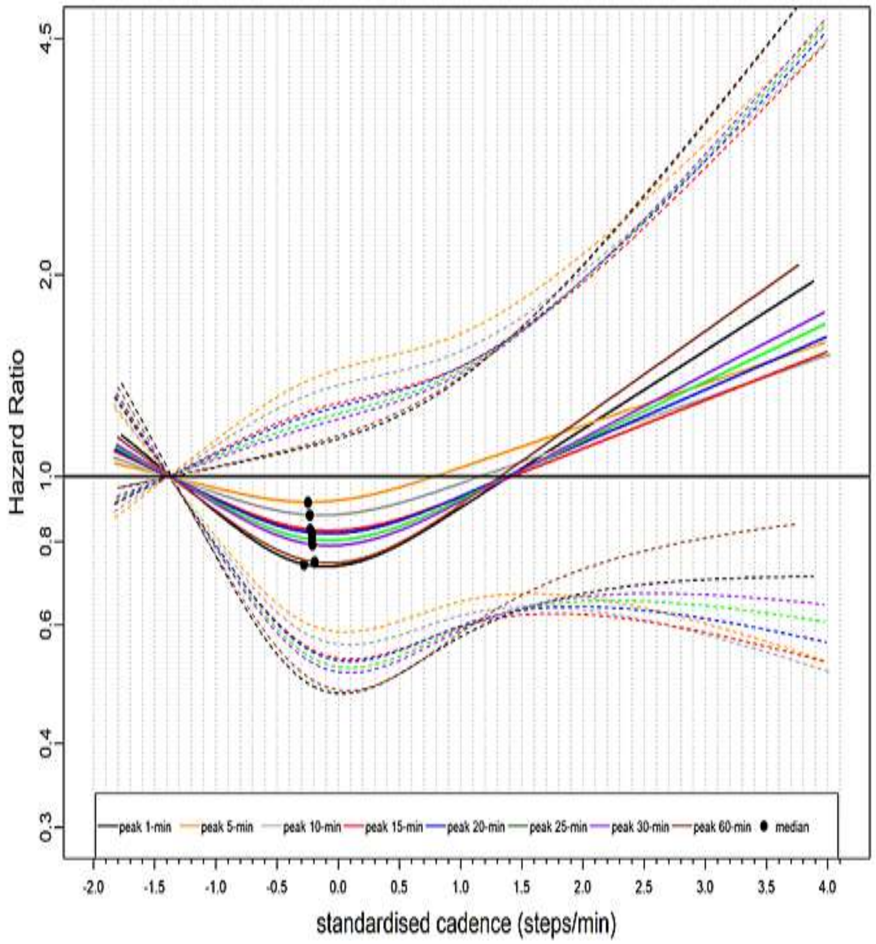


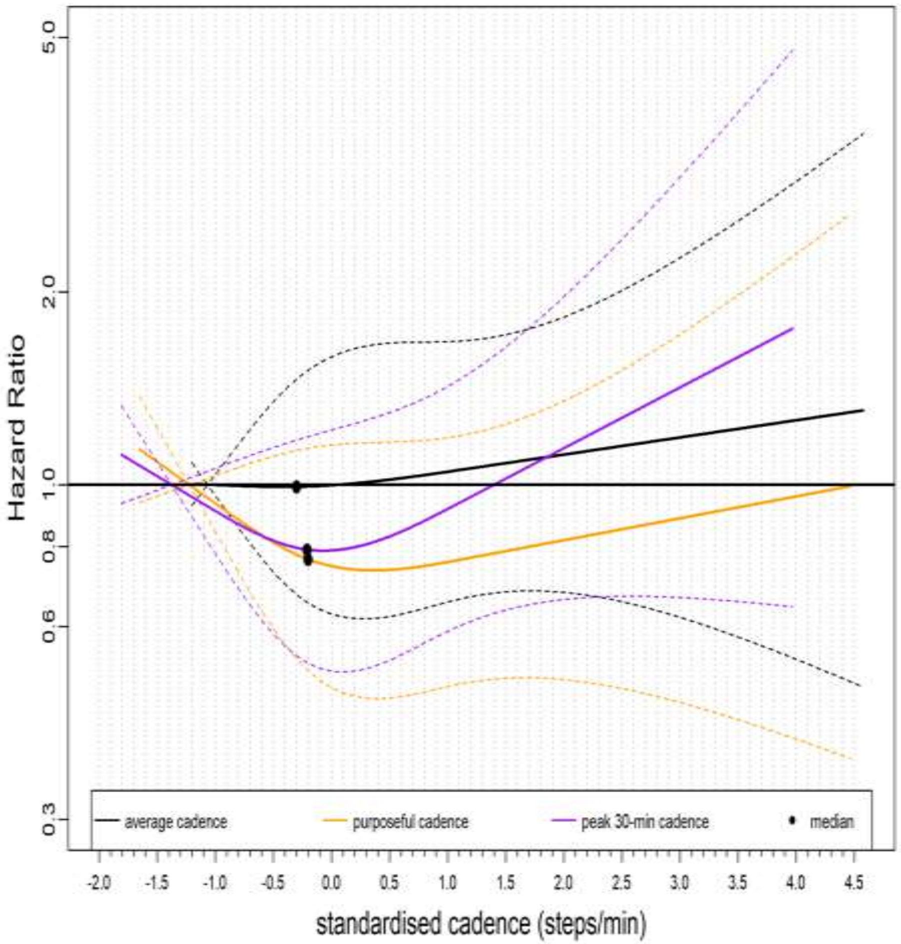

**b) Male**

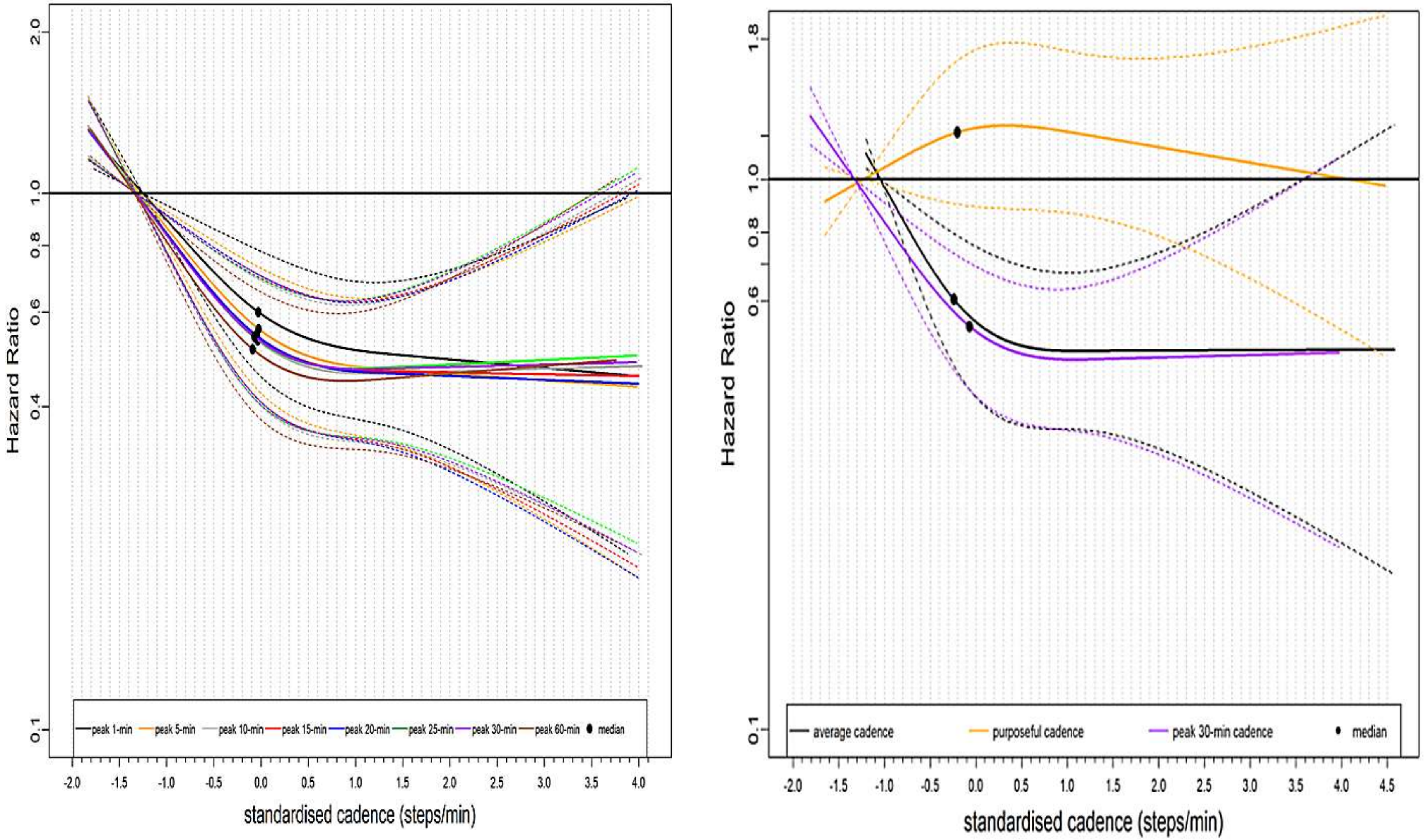
Hazard Ratio
2.0
1.0
0.8
0.6
0.4
0.1
peak 1-min
peak 5-min
peak 10-min
peak 15-min
peak 20-min
peak 25-min
peak 30-min
peak 60-min
median
-2.0
-1.5
-1.0
-0.5
0.0
0.5
1.0
1.5
2.0
2.5
3.0
3.5
4.0
standardised cadence (steps/min)
Hazard Ratio
1.8
1.0
0.8
0.6
0.1
average cadence
purposeful cadence
peak 30-min cadence
median
-2.0
-1.5
-1.0
-0.5
0.0
0.5
1.0
1.5
2.0
2.5
3.0
3.5
4.0
4.5
standardised cadence (steps/min)

The circle indicates the median cadence. We analysed the dose-response association using Fine and Grey model and adjusted for age, sex, accelerometer wearing duration, average daily steps, smoking status, alcohol consumption, sleep duration, Townsend deprivation score, sedentary time, education levels, self-reported parental history of CVD and cancer, and self-reported medication use (cholesterol, blood pressure, and diabetes). The reference level is 5th percentile of the distribution of each exposure.

**Supplemental Figure 18** Sex-specific Dose-response Association of Standardised Stepping Intensity Estimated by Peak Cadence Metrics with Cancer Mortality

**a) Female**

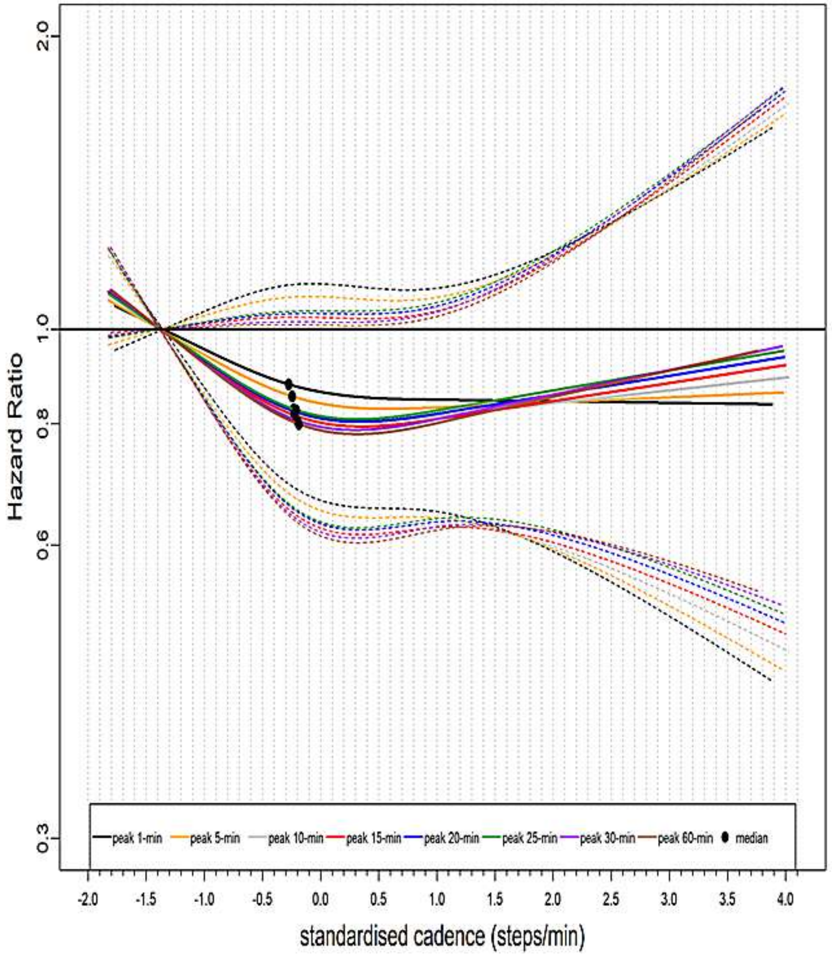


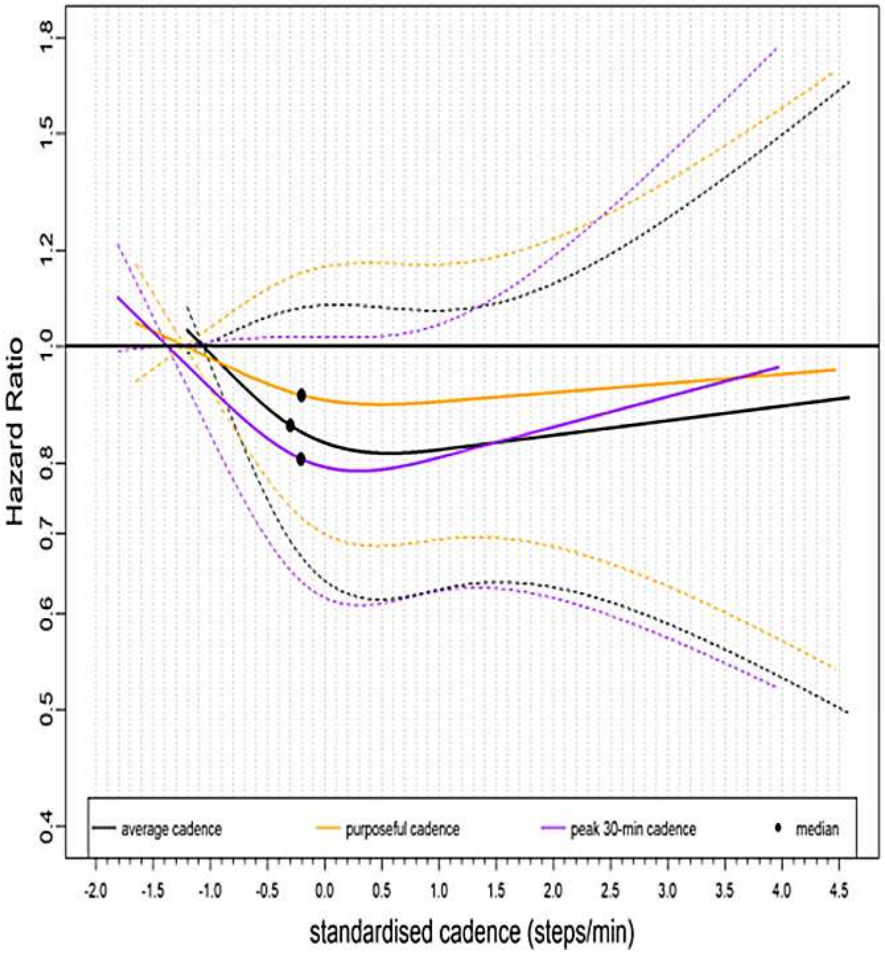

**b) Male**

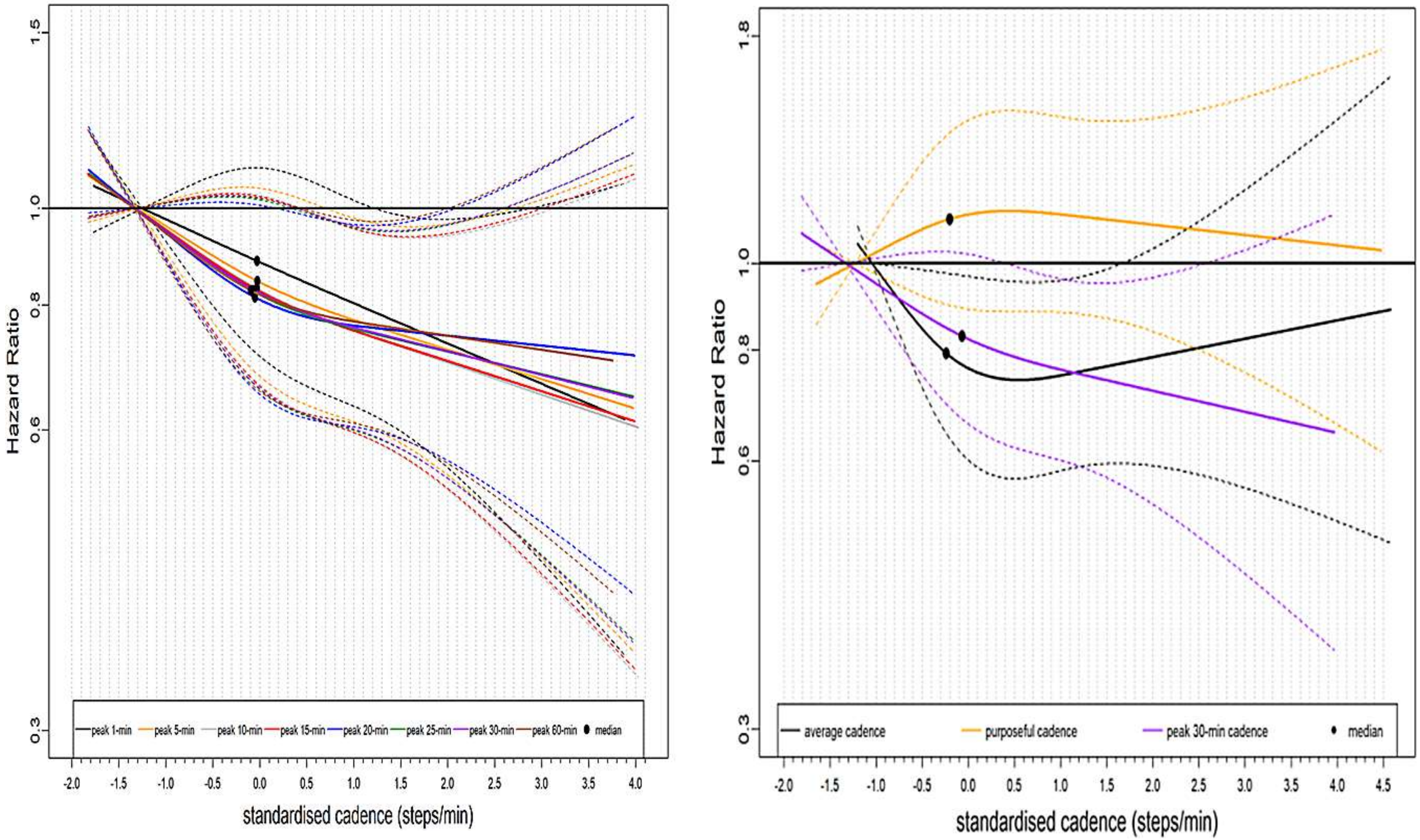

Hazard Ratio
1.5
1.0
0.8
0.6
0.3
peak 1-min
peak 5-min
peak 10-min
peak 15-min
peak 20-min
peak 25-min
peak 30-min
peak 60-min
median
-2.0
-1.5
-1.0
-0.5
0.0
0.5
1.0
1.5
2.0
2.5
3.0
3.5
4.0
standardised cadence (steps/min)
Hazard Ratio
1.8
1.0
0.8
0.6
0.3
average cadence
purposeful cadence
peak 30-min cadence
median
-2.0
-1.5
-1.0
-0.5
0.0
0.5
1.0
1.5
2.0
2.5
3.0
3.5
4.0
4.5
standardised cadence (steps/min)

The circle indicates the median cadence. We analysed the dose-response association using Fine and Grey model and adjusted for age, sex, accelerometer wearing duration, average daily steps, smoking status, alcohol consumption, sleep duration, Townsend deprivation score, sedentary time, education levels, self-reported parental history of CVD and cancer, and self-reported medication use (cholesterol, blood pressure, and diabetes). The reference level is 5$^{th}$ percentile of the distribution of each exposure.

**Supplemental Figure 19** PA-level Specific Dose-response Association of Standardised Stepping Intensity Estimated by Peak and Non-peak Cadence Metrics with All-cause Mortality

**a) 0-5,000 steps/day**

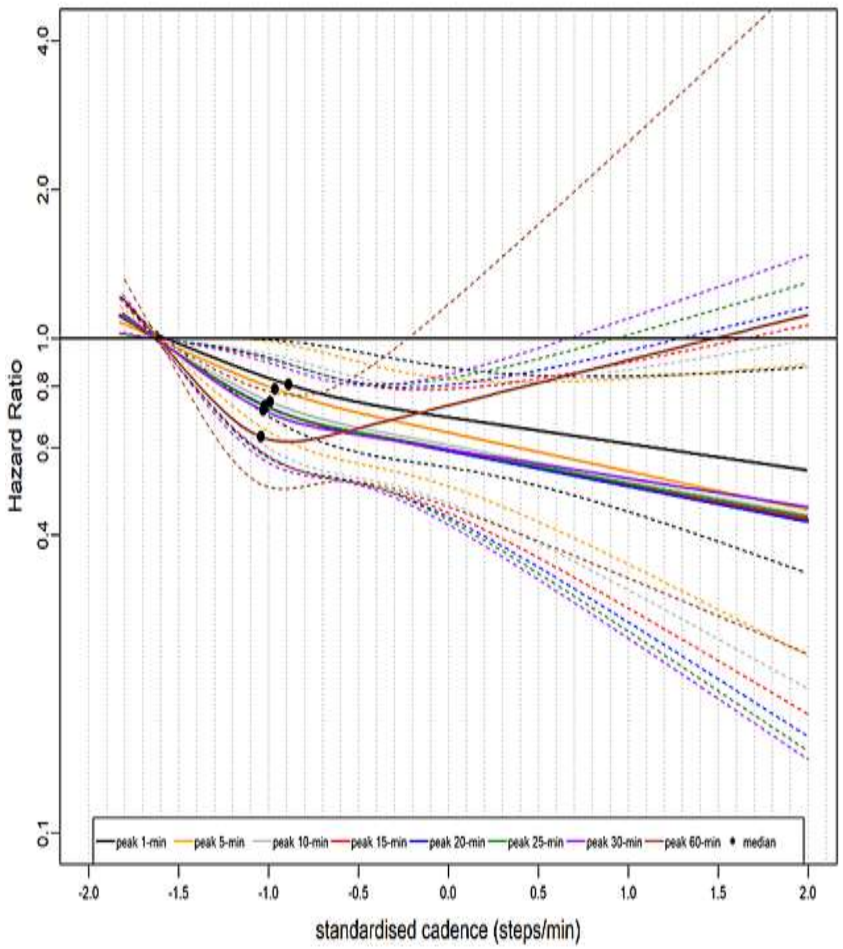


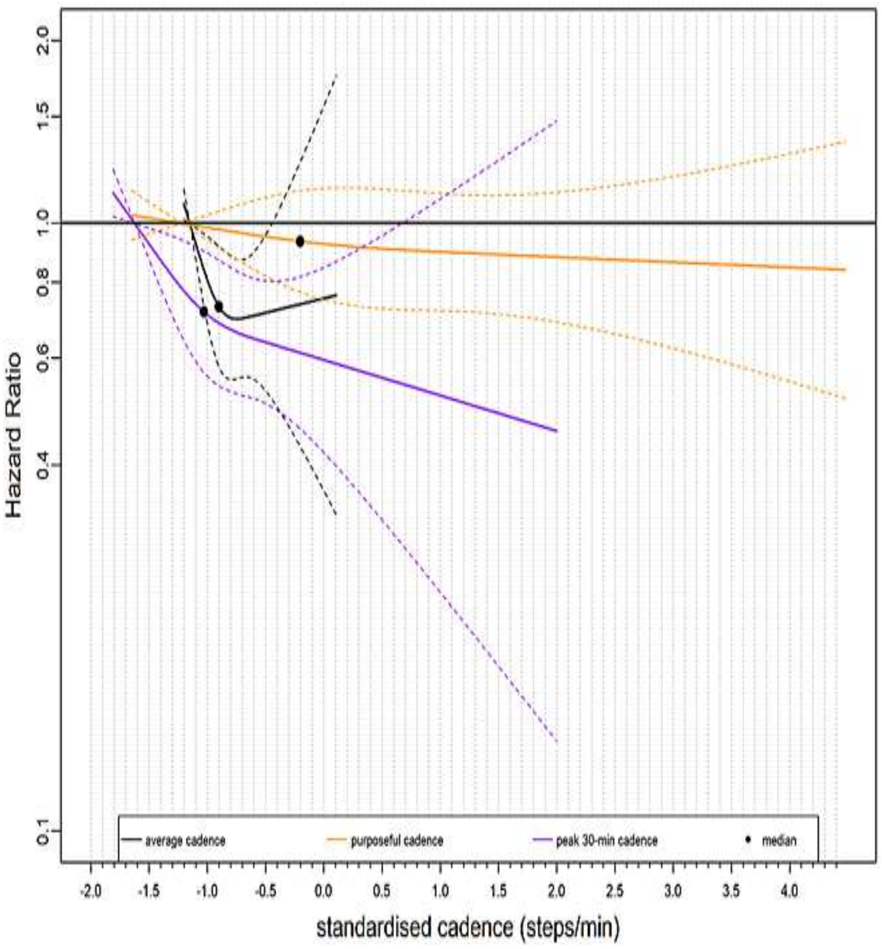

**Note:** the dose-response association of standardised cadence (steps/min) with all-cause mortality across peak cadence metrics (left figure) was capped at 2.0 steps/min due to wide confidence interval

**b) 5,000-7,500 steps/day**

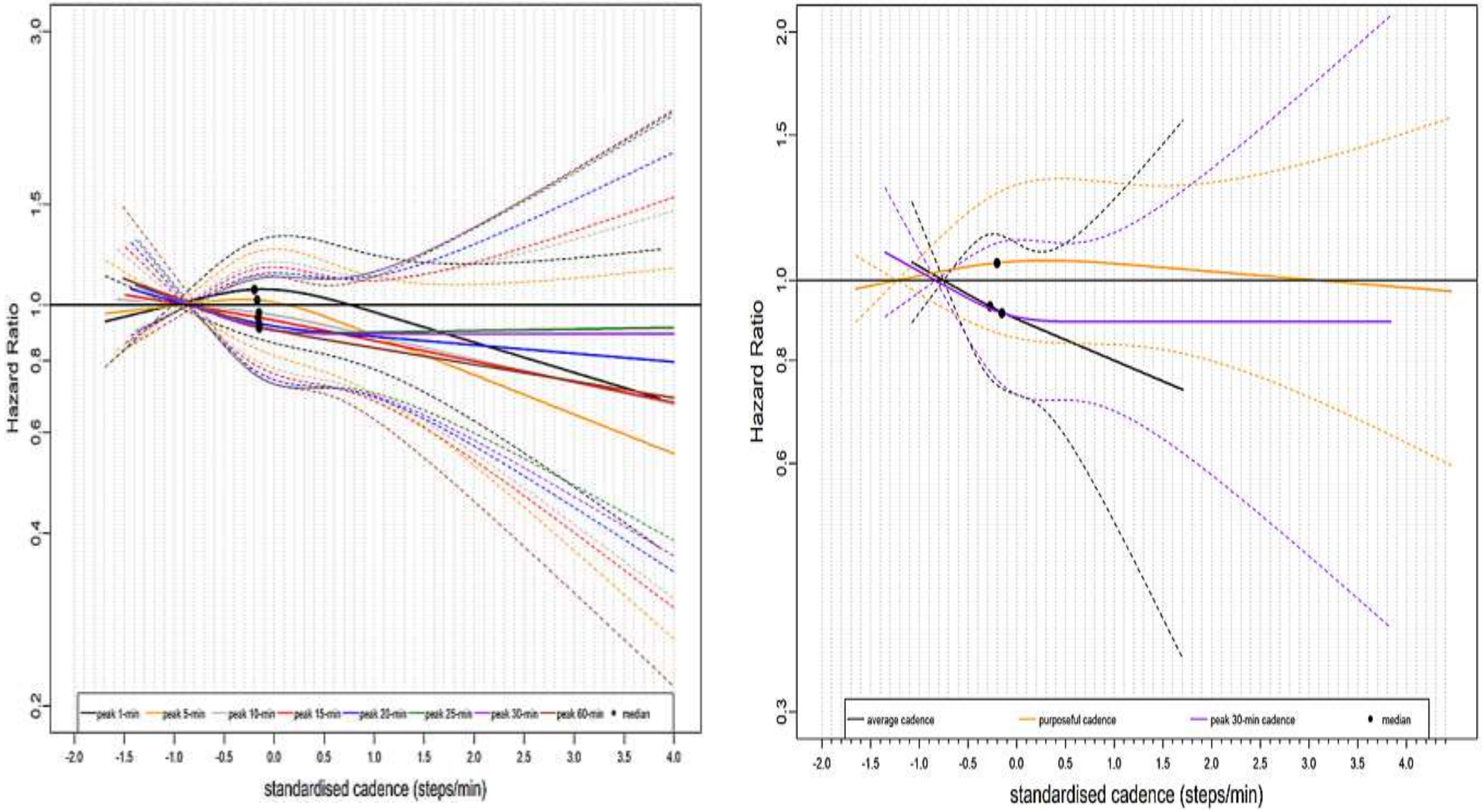

c) **7,500-10,000 steps/day**

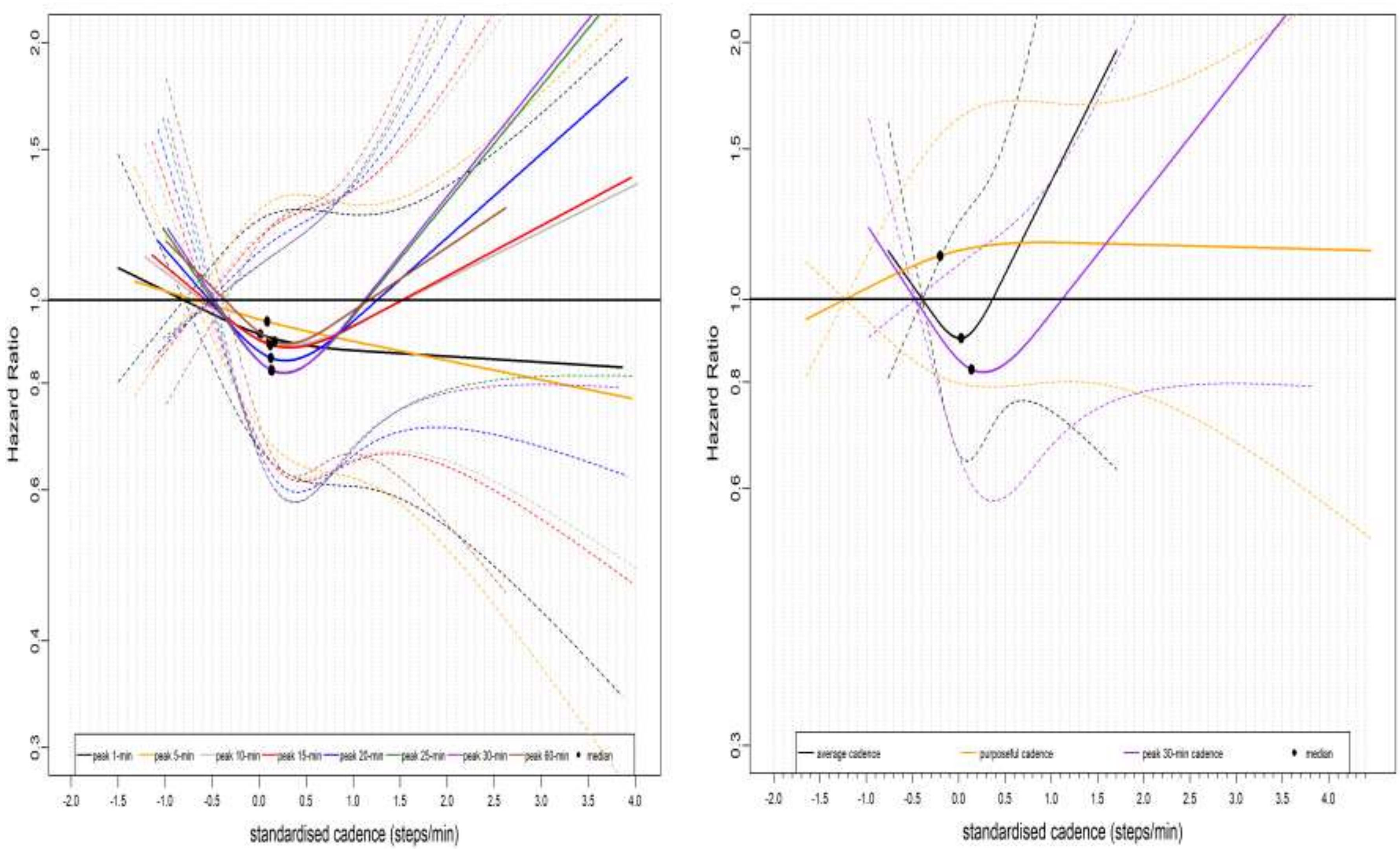

**d) Above 10,000 steps/day**

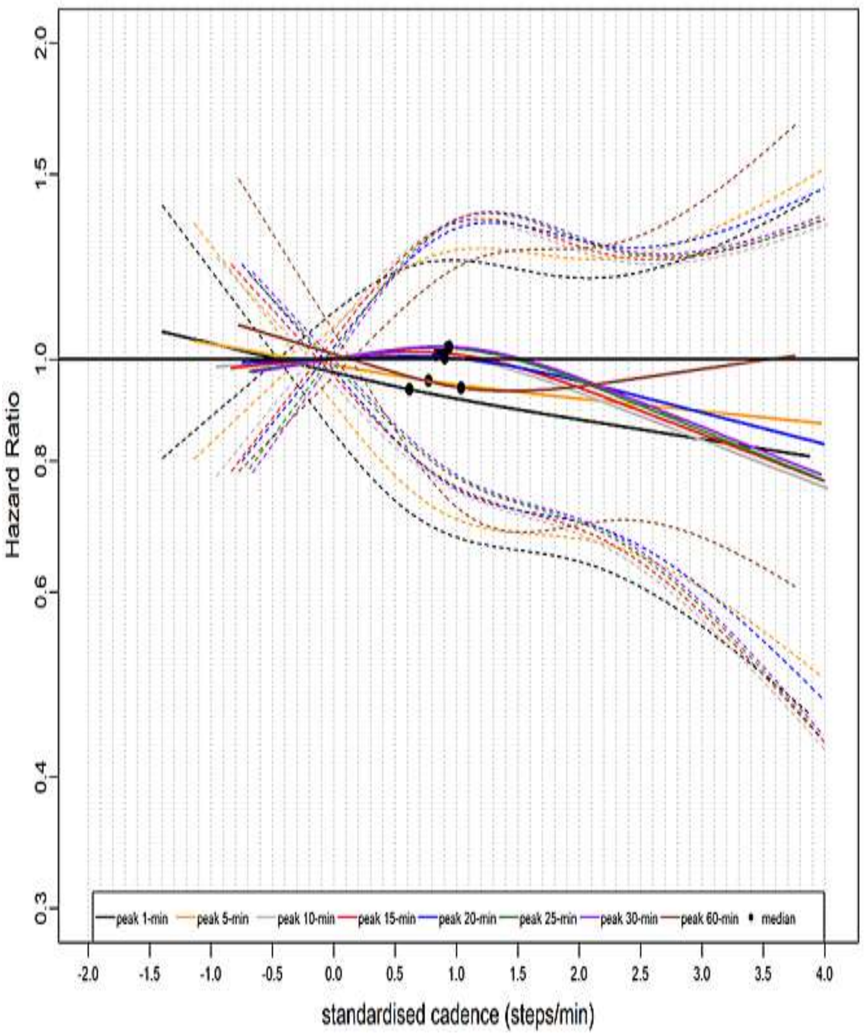


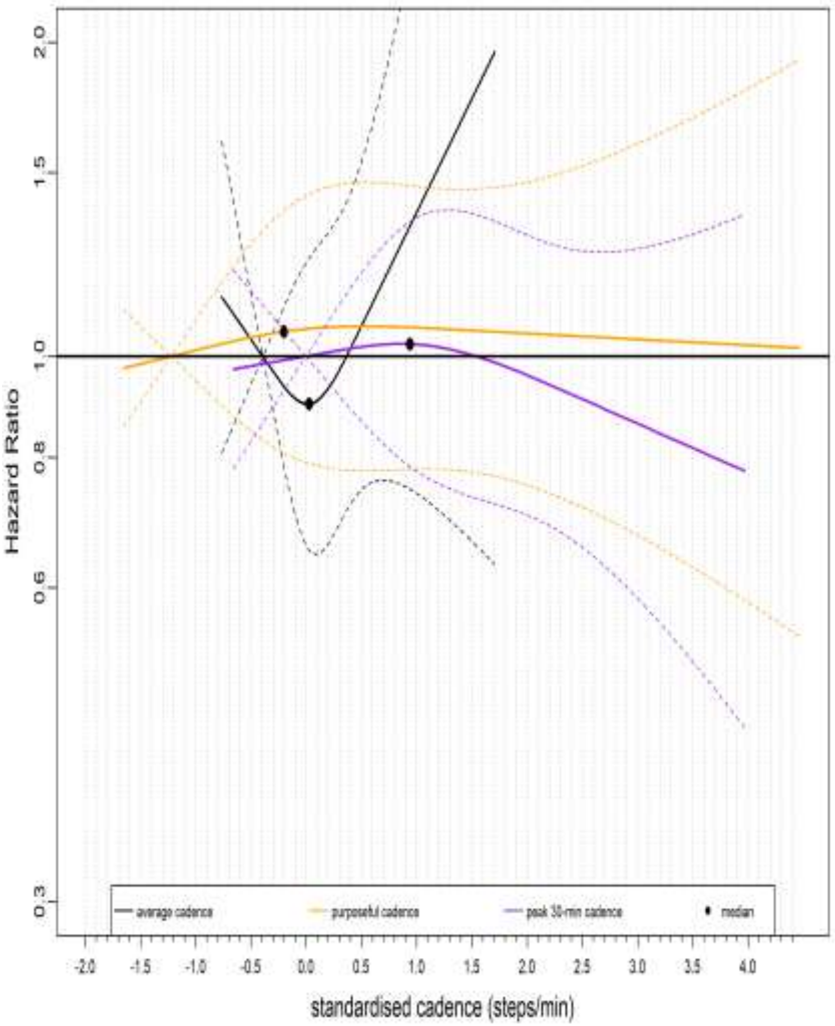

The circle indicates the median cadence. The dose-response association was analysed using cox-regression model and adjusted for age, sex, accelerometer wearing duration, average daily steps, smoking status, alcohol consumption, sleep duration, townsend deprivation score, sedentary time, education levels, self-reported parental history of CVD and cancer, and self-reported medication use (cholesterol, blood pressure, and diabetes). The reference level is 5$^{th}$ percentile of each peak cadence metric.

**Supplemental Figure 20** PA-level Specific Dose-response Association of Standardised Stepping Intensity Estimated by Peak and Non-peak Cadence Metrics with CVD Mortality

a) **0-5,000 steps/day**

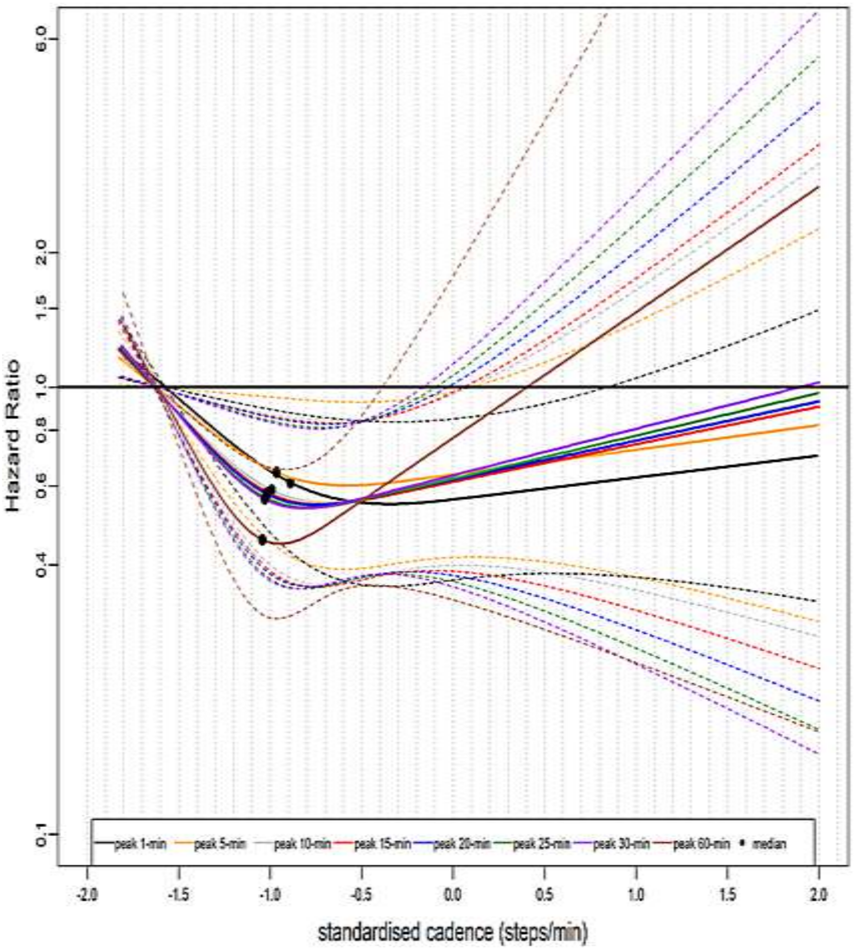


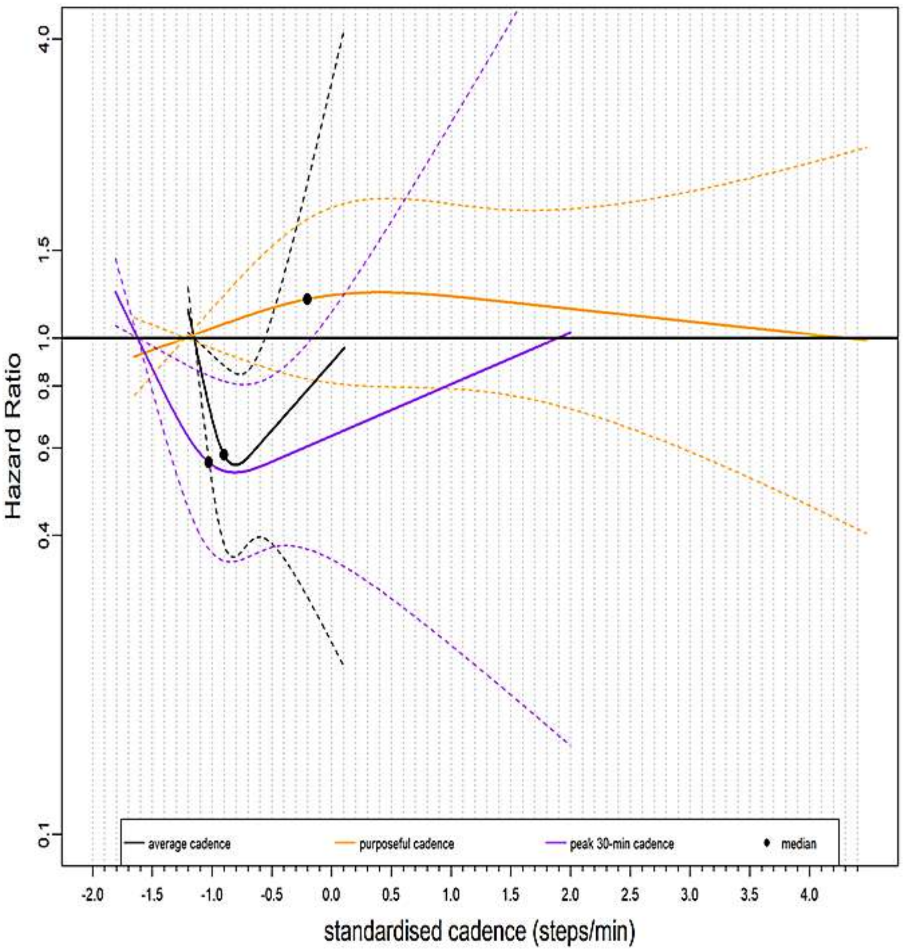

**Note:** the dose-response association of standardised cadence (steps/min) with CVD mortality across peak cadence metrics (left figure) was capped at 2.0 steps/min due to wide confidence interval

**b) 5,000 – 7,500 steps/day**

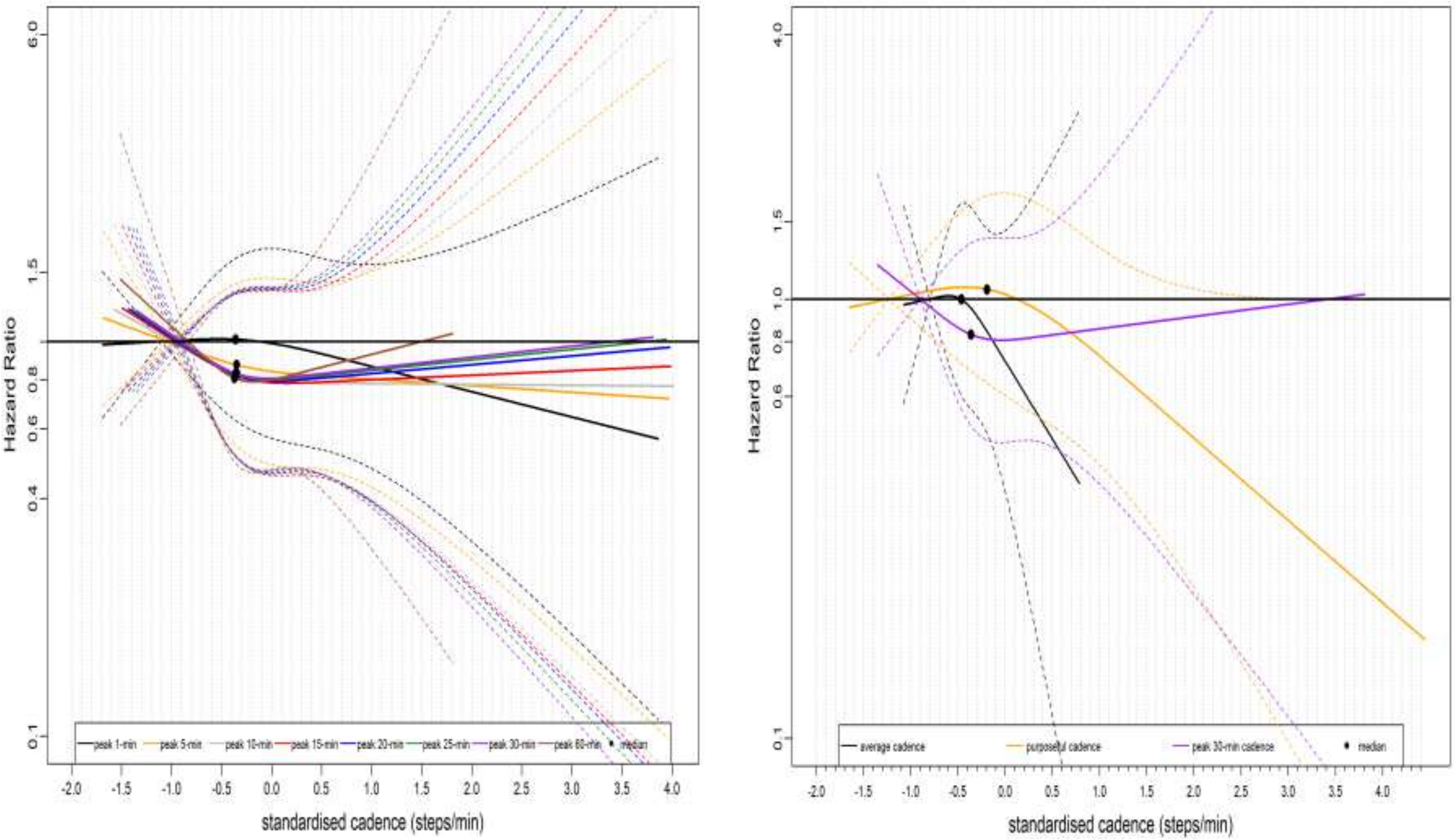

## c) 7,500 - 10,000 steps/day

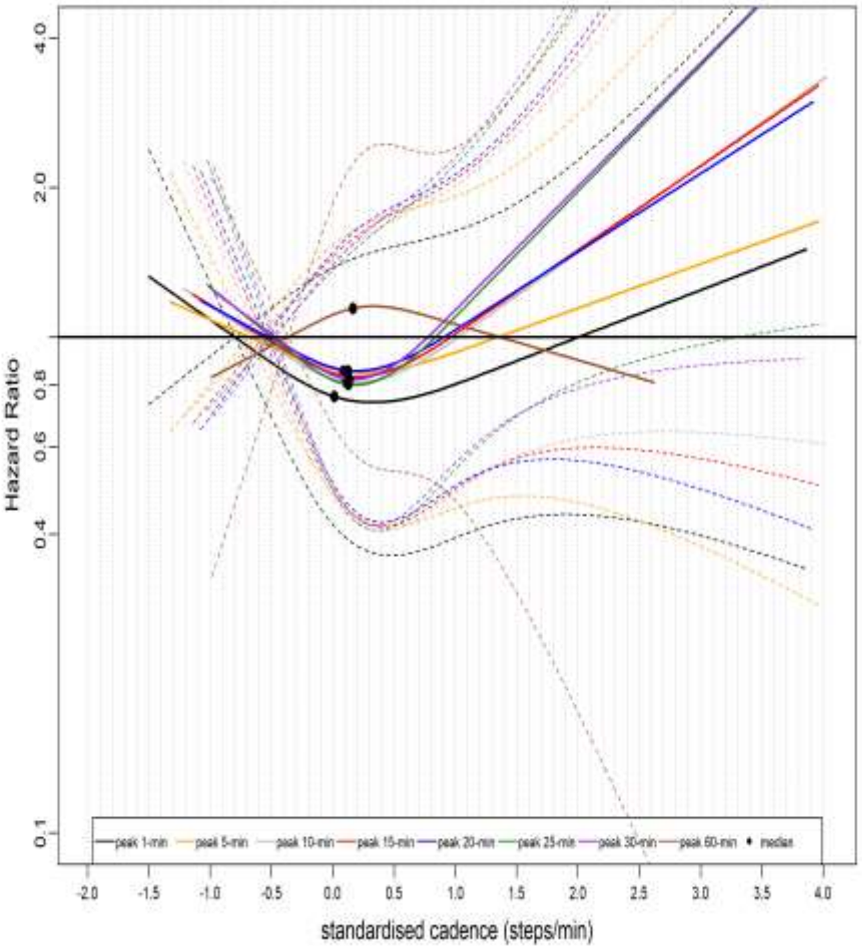


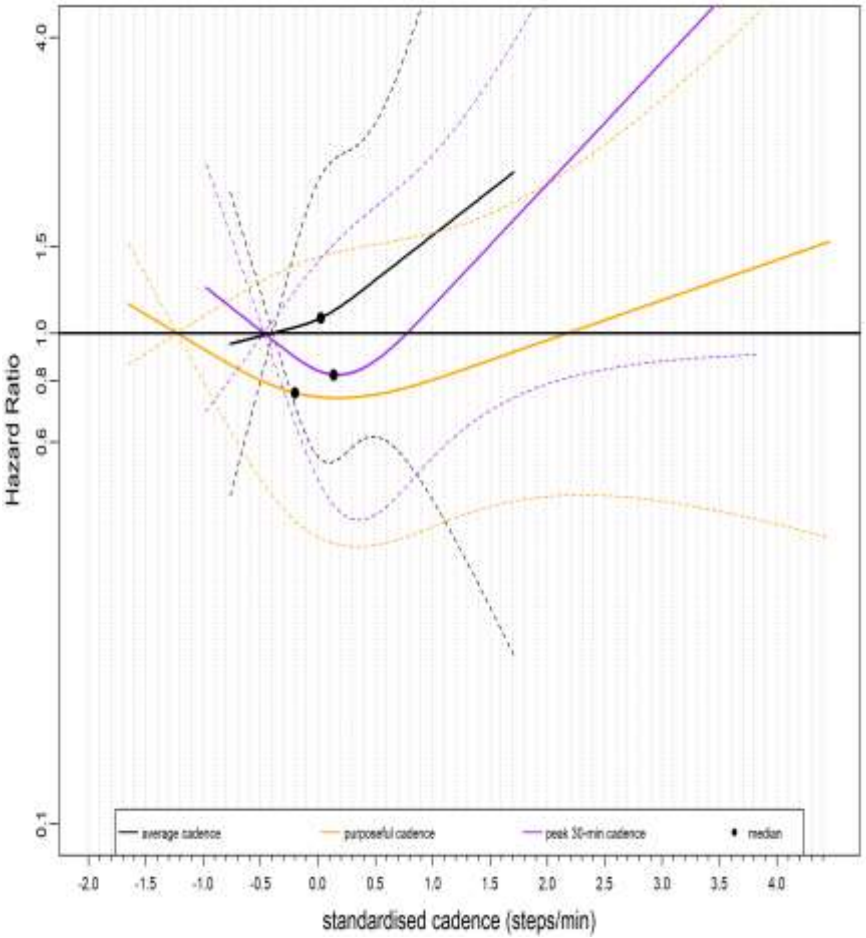

**d) Above 10,000 steps/day**

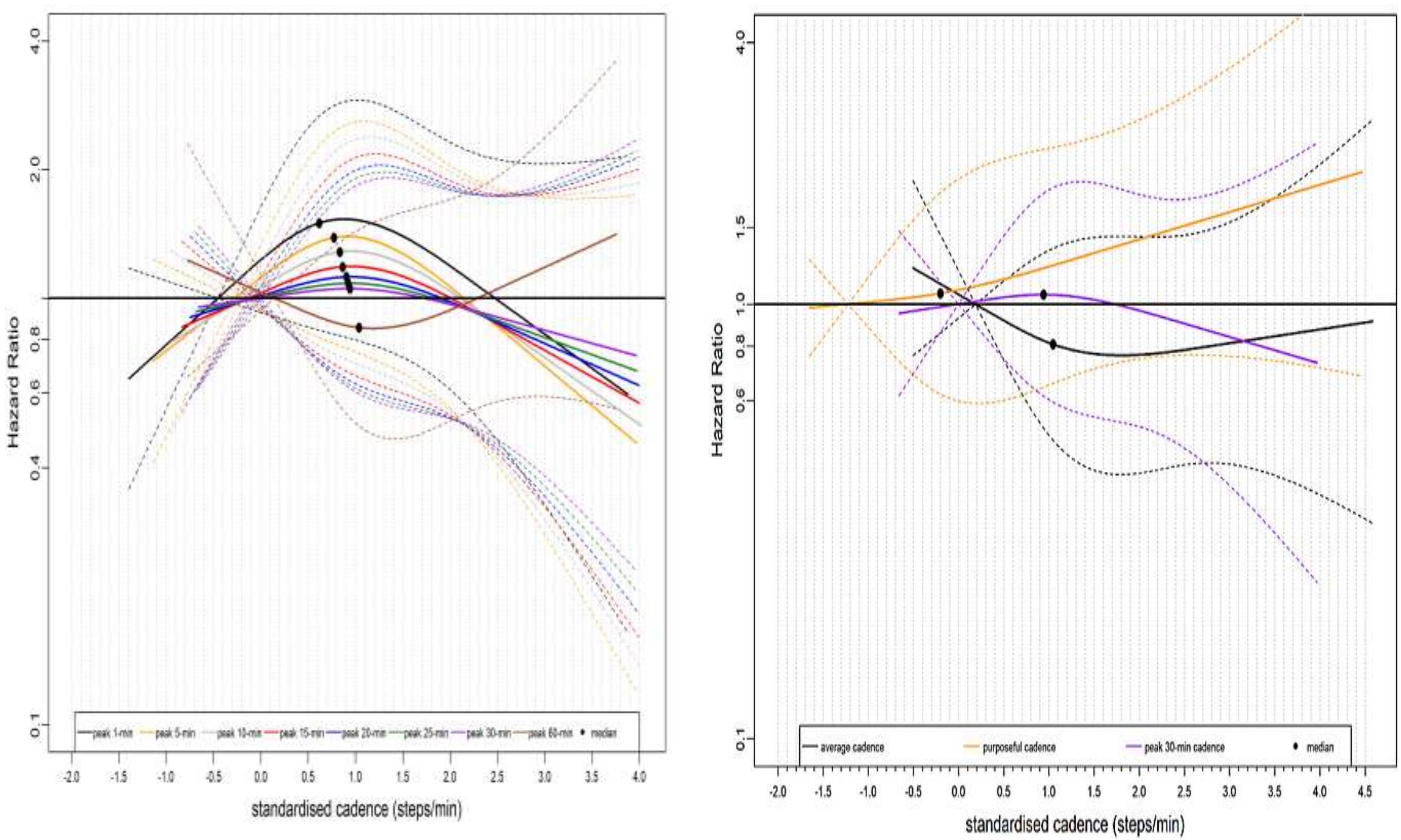

The circle indicates the median cadence. We analysed the dose-response association using Fine and Grey model and adjusted for age, sex, accelerometer wearing duration, average daily steps, smoking status, alcohol consumption, sleep duration, Townsend deprivation score, sedentary time, education levels, self-reported parental history of CVD and cancer, and self-reported medication use (cholesterol, blood pressure, and diabetes). The reference level is 5th percentile of the distribution of each exposure.

**Supplemental Figure 21** PA-level Specific Dose-response Association of Standardised Stepping Intensity Estimated by Peak and Non-peak Cadence Metrics with Cancer Mortality

**a) 0-5,000 steps/day**

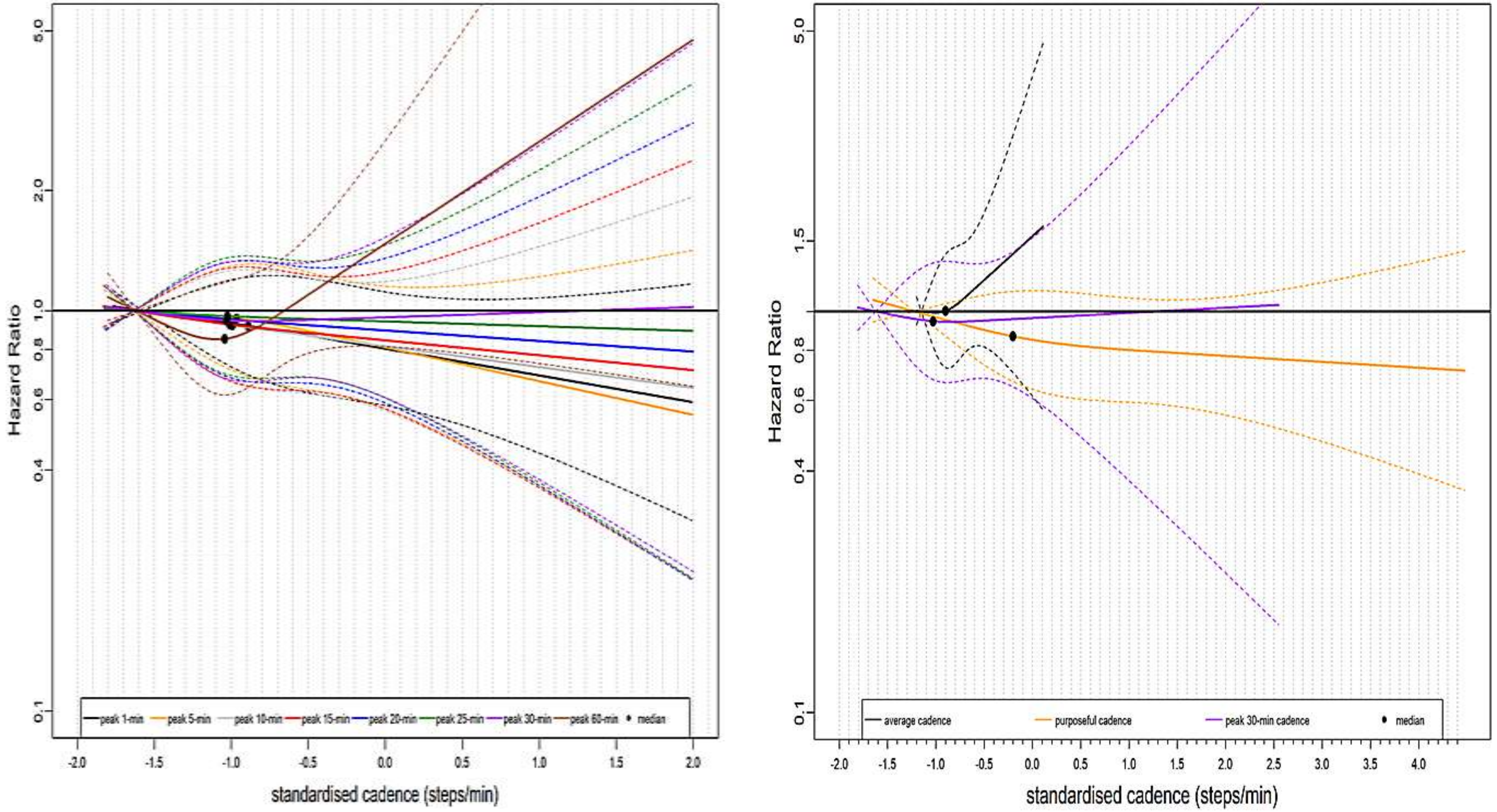

**Note:** the dose-response association of standardised cadence (steps/min) with cancer mortality across peak cadence metrics (left figure) was capped at 2.0 steps/min due to wide confidence interval

**b) 5,000-7,500 steps/day**

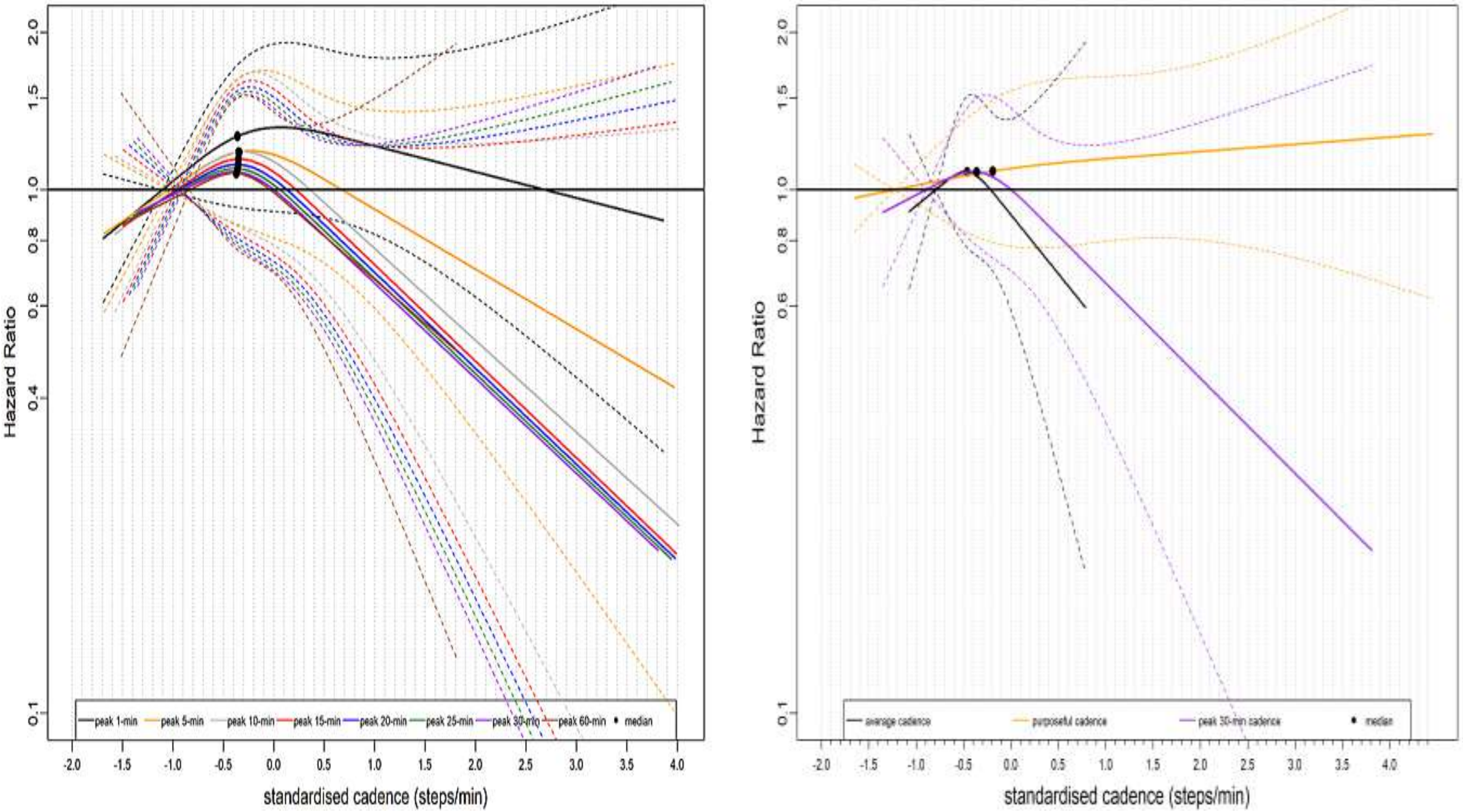

c) **7,500-10,000 steps/day**

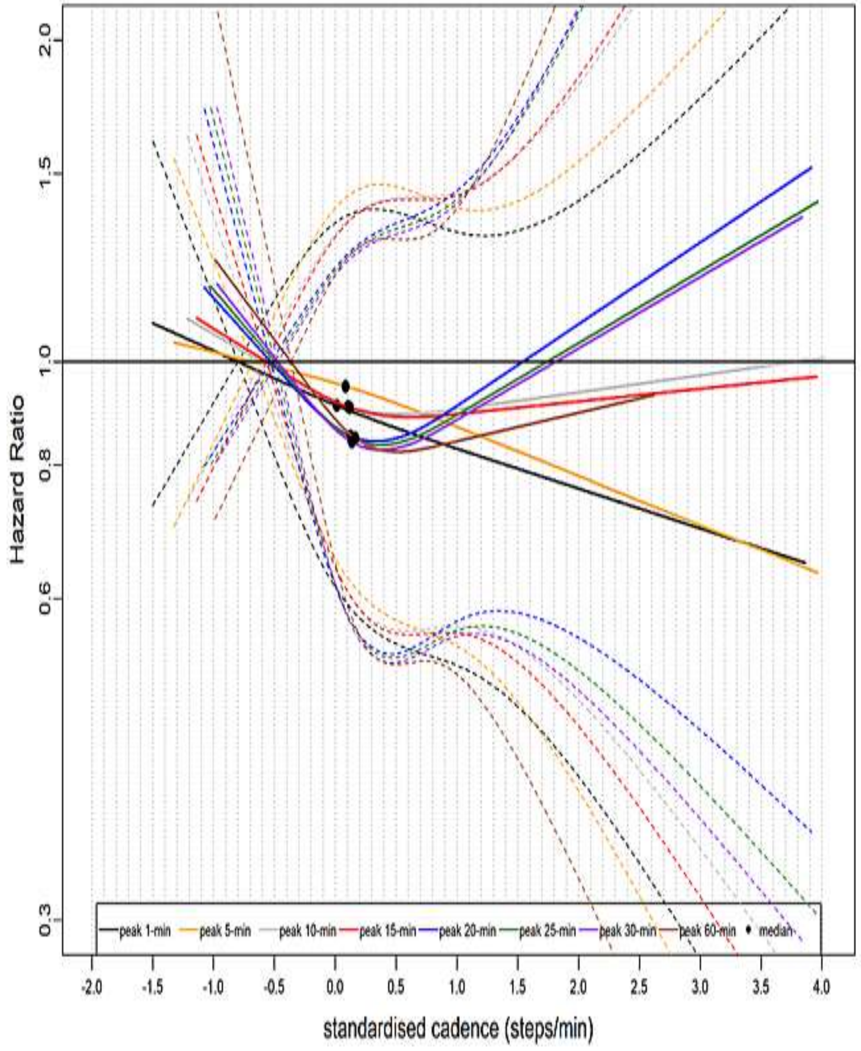


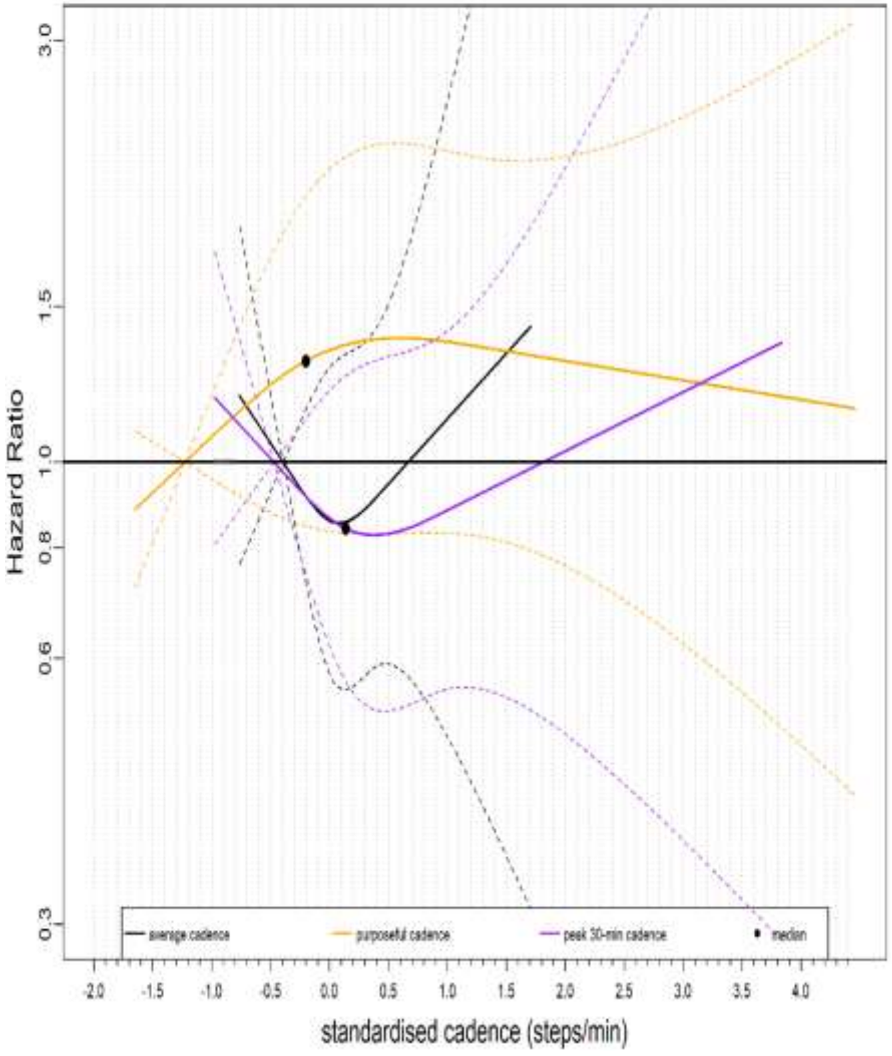

**d) Above 10,000 steps/day**

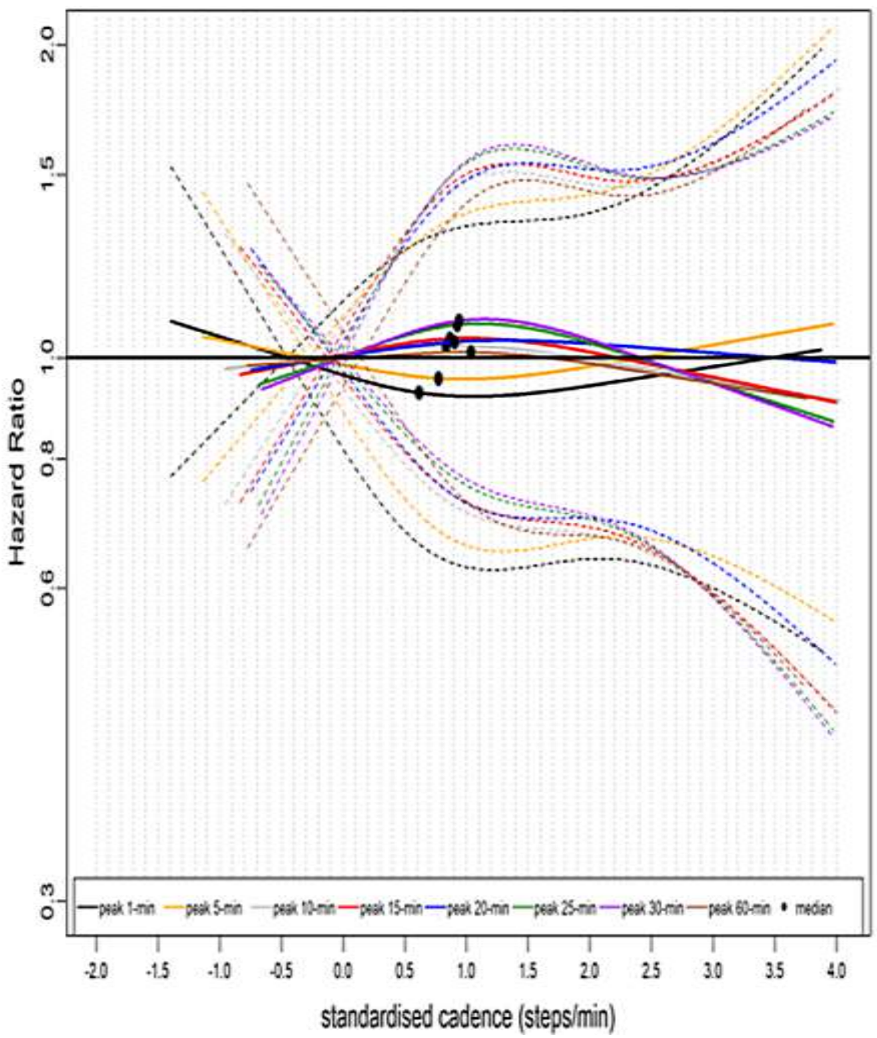


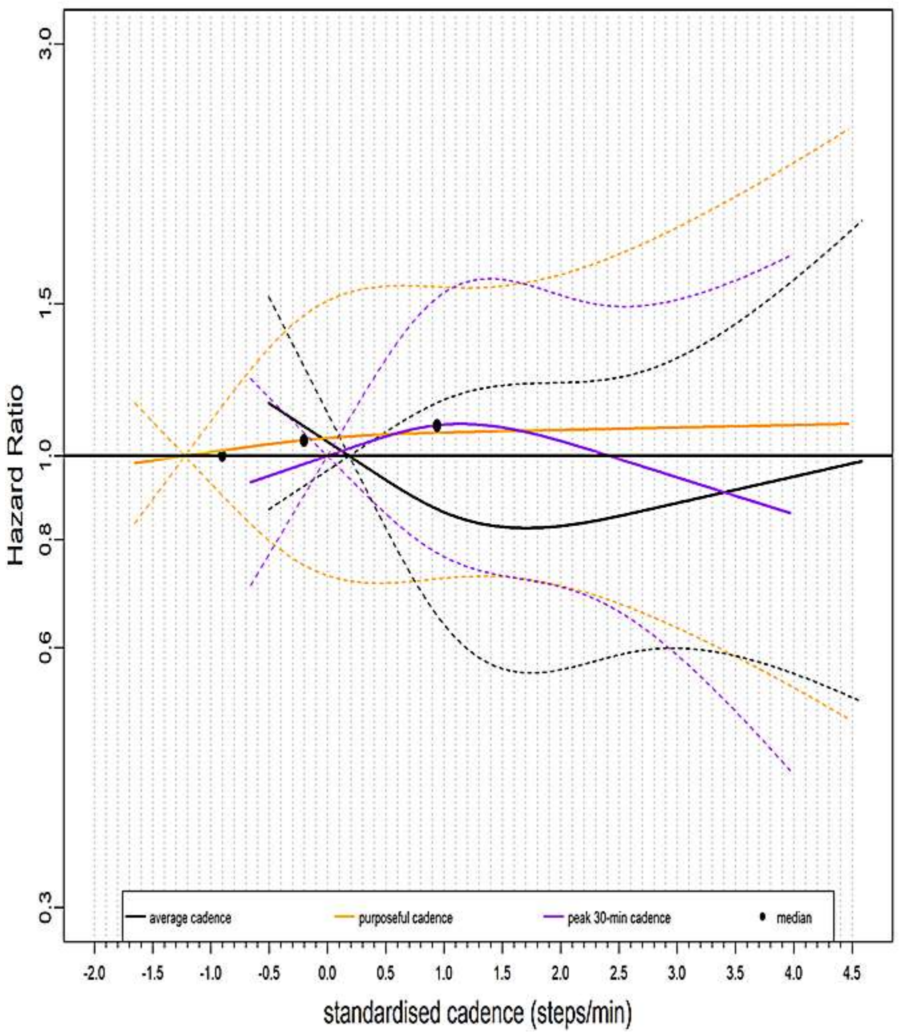

The circle indicates the median cadence. We analysed the dose-response association using Fine and Grey model and adjusted for age, sex, accelerometer wearing duration, average daily steps, smoking status, alcohol consumption, sleep duration, Townsend deprivation score, sedentary time, education levels, self-reported parental history of CVD and cancer, and self-reported medication use (cholesterol, blood pressure, and diabetes). The reference level is 5th percentile of the distribution of each exposure.

**Supplementary Table 1** Baseline Characteristics of Study Participants by Quartiles of Peak 30-min Cadence Per Day [a]

| Characteristics | Overall | Bottom Quartile | Q2 | Q3 | Top Quartile |
|---|---|---|---|---|---|
| Participants, No. | 65,253 | 16,314 | 16,314 | 16,313 | 16,312 |
| Sex | | | | | |
| Female | 37,316 (57.2) | 10,061 (61.7) | 9,577 (58.7) | 9,170 (56.2) | 8,508 (52.2) |
| Male | 27,937 (42.8) | 6,253 (38.3) | 6,737 (41.3) | 7,143 (43.8) | 7,804 (47.8) |
| Age | 61.5 (7.8) | 63.4 (7.6) | 61.9 (7.7) | 61.0 (7.7) | 59.9 (7.8) |
| Accelerometer wearing days | 6.7 (0.5) | 6.9 (0.40) | 6.9 (0.4) | 6.9 (0.4) | 6.9 (0.4) |
| Follow-up years | 8.0 (0.9) | 7.9 (0.9) | 8.0 (0.9) | 8.0 (0.8) | 8.0 (0.8) |
| Stepping intensity metrics [b] | | | | | |
| Peak 1-min cadence | 170.0 (55.6) | 151.8 (30.6) | 178.6 (36.1) | 229.5 (53.6) | 120.0 (28.8) |
| Peak 5-min cadence | 134.0 (43.0) | 117.1 (14.3) | 141.1 (17.7) | 188.4 (36.8) | 89.3 (15.6) |

| | | | | | |
|---|---|---|---|---|---|
| Peak 10-min cadence | 115.8 (38.2) | 100.1 (9.1) | 122.3 (11.4) | 166.4 (30.5) | 74.5 (11.9) |
| Peak 15-min cadence | 104.9 (35.5) | 89.9 (7.0) | 110.9 (8.6) | 152.7 (27.3) | 65.9 (10.2) |
| Peak 20-min cadence | 97.0 (33.5) | 82.7 (5.9) | 102.7 (7.2) | 142.6 (25.2) | 59.9 (9.4) |
| Peak 25-min cadence | 90.8 (32.0) | 77.1 (5.3) | 96.3 (6.4) | 134.5 (23.8) | 55.3 (8.8) |
| Peak 30-min cadence | 85.7 (30.7) | 72.5 (5.1) | 91.0 (6.1) | 127.7 (22.6) | 51.6 (8.4) |
| Peak 60-min cadence | 58.5 (22.7) | 48.9 (4.9) | 62.6 (6.4) | 88.8 (17.9) | 33.7 (6.4) |
| Average_daily cadence | 3.1 (2.6) | 2.0 (0.5) | 3.3 (1.0) | 6.3 (2.9) | 0.9 (0.4) |
| Purposeful cadence | 67.9 (11.8) | 67.7 (11.4) | 67.9 (11.8) | 67.9 (12.0) | 67.9 (12.0) |
| Steps/day | 8,166(4,614) | 3,879 (1,252) | 6,369 (1,589) | 8,862 (2,386) | 13,555 (4,937) |
| Townsend Deprivation Score [c] | -1.8 (2.8) | -1.7 (2.8) | -1.9 (2.8) | -1.8 (2.8) | -1.7 (2.8) |
| Alcohol_status, No. (%) [d] | | | | | |
| Above_guideline | 24,660 (57.2) | 5,738 (34.1) | 6,080 (37.3) | 6,342 (38.9) | 6,500 (39.8) |

| | | | | | |
|---|---|---|---|---|---|
| Never | 1,688 (2.6) | 497 (3.0) | 413 (2.5) | 394 (2.6) | 384 (2.4) |
| Previous | 1,591 (2.4) | 435 (2.7) | 394 (2.4) | 390 (2.4) | 372 (2.3) |
| Under_guideline | 37,314 (57.2) | 9,644 (59.1) | 9,427 (57.8) | 9,187 (56.3) | 9,056 (55.5) |
| Screen time | 3.9 (1.9) | 4.2 (2.0) | 3.9 (1.9) | 3.8 (1.9) | 3.6 (1.8) |
| Sleep hours/day | 7.5 (1.3) | 7.4 (1.5) | 7.5 (1.3) | 7.5 (1.2) | 7.5 (1.2) |
| College or University degree, No. (%) | 31,635 (45.0) | 7,425 (42.2) | 7,709 (43.8) | 7,979 (45.4) | 8,522 (48.5) |
| Cholesterol medication, No. (%) | 7148 (10.9) | 2,483 (15.2) | 1,892 (11.6) | 1,549 (9.5) | 1,224 (7.5) |
| Hypertension medication, No. (%) | 5,234 (8.0) | 1,665 (10.2) | 1,397 (8.6) | 1,188 (7.3) | 984 (6.0) |
| Insulin medication, No. (%) | 79 (0.1) | 19 (0.1) | 21 (0.1) | 20 (0.1) | 19 (0.1) |

| | | | | | |
|---|---|---|---|---|---|
| Smoking_status, No. (%) | | | | | |
| Never | 38,215 (58.6) | 9,168 (56.2) | 9,451 (57.9) | 9,697 (59.4) | 9,899 (60.7) |
| Previous | 22,806 (35.0) | 5,907 (36.2) | 5,769 (35.4) | 5,615 (34.4) | 5,515 (33.8) |
| Current | 4,232 (6.5) | 1,239 (7.6) | 1,094 (6.7) | 1,001 (6.1) | 898 (5.5) |
| Family history of CVD, No. (%) | 35,398 (54.2) | 9,223 (56.5) | 8,924 (54.7) | 8,755 (53.7) | 8,496 (52.1) |
| Family history of cancer, No. (%) | 20,161 (30.9) | 5,125 (31.4) | 5,167 (31.7) | 4,939 (30.3) | 4,930 (30.2) |
| All-cause mortality, No. (%) | 1,736 (2.9) | 630 (4.2) | 425 (2.9) | 353 (2.3) | 328 (2.2) |
| CVD mortality, No. (%) | 466 (0.8) | 181 (1.3) | 106 (0.7) | 91 (0.6) | 88 (0.6) |
| Cancer mortality, No. (%) | 1,038 (1.7) | 339 (2.2) | 280 (1.9) | 215 (1.4) | 204 (1.3) |

Abbreviations: CVD, cardiovascular disease; PA, physical activity.

[a.] cadence was defined as steps per min.

[b.] Values represent mean (SD), unless specified otherwise.

[c] Lower score indicates more affluence

[d] Guidelines for alcohol use in the UK recommend no more than 14 units (1 unit = 10mL of pure alcohol) per week for both men and women.

**Supplementary Table 2** Hazard Ratio of Cancer Mortality at -0.5, 3.0 and Median Standardised Steps per Min Estimated by Peak Cadence and Non-Peak Cadence Metrics

| | -0.5 standardised steps/min | 3.0 standardised steps/min | Standardised median cadence | |
|---|---|---|---|---|
| **Peak cadence metrics** | HR (95% CI) | HR (95% CI) | steps/ min | HR (95% CI) |
| Peak 1-min cadence | 0.91(0.80, 1.03) | 0.76 (0.57, 1.01) | -0.17 | 0.88 (0.75, 1.03) |
| Peak 5-min cadence | 0.89 (0.78, 1.01) | 0.73 (0.53, 1.00) | -0.16 | 0.85 (0.73, 1.00) |
| Peak 10-min cadence | 0.87 (0.77, 0.99) | 0.72 (0.53, 0.99) | -0.15 | 0.84 (0.71, 0.98) |
| Peak 15-min cadence | 0.87 (0.76, 0.98) | 0.73 (0.54, 1.00) | -0.15 | 0.83 (0.71, 0.97) |
| Peak 20-min cadence | 0.86 (0.76, 0.98) | 0.78 (0.58, 1.07) | -0.14 | 0.82 (0.70, 0.96) |
| Peak 25-min | 0.87 (0.76, 0.98) | 0.76 (0.56, 1.03) | -0.15 | 0.83 (0.71, 0.97) |

| | | | | |
|---|---|---|---|---|
| cadence | | | | |
| Peak 30-min cadence | 0.86 (0.76, 0.98) | 0.76 (0.56, 1.04) | -0.15 | 0.82 (0.70, 0.96) |
| Peak 60-min cadence | 0.86 (0.76, 0.97) | 0.79 (0.58, 1.07) | -0.14 | 0.82 (0.70, 0.96) |
| Purposeful cadence | 1.02 (0.90, 1.15) | 1.01 (0.78, 1.31) | 0.65 | 1.02 (0.85, 1.23) |
| Average daily cadence | 0.86 (0.77, 0.97) | 0.84 (0.64, 1.10) | 0.69 | 0.77 (0.64, 0.93) |

Note: For better comparison, we standardised the cadence across stepping intensity metrics to rescale them into comparable range. The standardised cadence was calculated as ([exposure - mean] / standard deviation). Each indicator was centered around mean of 0 with standard deviation of 1.

**Supplementary Table 3** Hazard Ratio of All-Cause Mortality Associated with the Minimum, Median and Maximum Stepping Intensities (in Absolute Cadence Value), Estimated by Peak Cadence and Non-Peak Cadence Metrics

| Stepping intensity | minimum cadence | | median cadence | | maximum cadence | |
|---|---|---|---|---|---|---|
| | steps/min | HR (95% CI) | steps/min | HR (95% CI) | steps/min | HR (95% CI) |
| **Peak cadence metrics** | | | | | | |
| Peak 1-min cadence | 124.8 | 0.83 (0.77, 0.89) | 146.9 | 0.72 (0.63, 0.81) | — | — |
| Peak 5-min cadence | 101.2 | 0.81 (0.76, 0.88) | 123.7 | 0.70(0.62, 0.79) | — | — |
| Peak 10-min cadence | 85.9 | 0.81 (0.76, 0.87) | 109.7 | 0.67(0.60,0.76) | 146.2 | 0.62 (0.54, 0.71) |
| Peak 15-min cadence | 77.1 | 0.81(0.76, 0.86) | 100.0 | 0.67 (0.59, 0.75) | 132.5 | 0.62 (0.54, 0.70) |
| Peak 20-min cadence | 70.3 | 0.81 (0.76, 0.86) | 92.1 | 0.66 (0.59, 0.75) | 120.0 | 0.62 (0.54, 0.70) |
| Peak 25-min cadence | 65.4 | 0.81 (0.76, 0.86) | 86.1 | 0.67 (0.59, 0.75) | 113.2 | 0.62 (0.54, 0.70) |
| Peak 30-min | 72.8 | 0.71 (0.64, | 81.2 | 0.66 (0.59, | 106.7 | 0.62 |

| | | | | | | |
|---|---|---|---|---|---|---|
| cadence | | 0.79) | | 0.75) | | (0.54, 0.70) |
| Peak 60-min cadence | 40.0 | 0.80 (0.76, 0.85) | 55.1 | 0.65 (0.58, 0.73) | 70.6 | 0.61 (0.53, 0.69) |
| **Non-Peak cadence metrics** | | | | | | |
| Purposeful cadence | 91.4 | 0.97 (0.82, 1.16) | 65.4 | 1.01 (0.89, 1.14) | 110.5 | 0.94 (0.70, 1.27) |
| Average daily cadence | 1.6 | 0.82 (0.77, 0.88) | 2.4 | 0.72 (0.64, 0.81) | 4.5 | 0.64 (0.56, 0.74) |

Note: '—' indicates that there is no nadir point

**Supplementary Table 4** Hazard Ratio of CVD Mortality Associated with the Minimum, Median and Maximum Stepping Intensities (in Absolute Cadence Value), Estimated by Peak Cadence and Non-Peak Cadence Metrics

| **Stepping intensity** | minimum cadence | | median cadence | | maximum cadence | |
|---|---|---|---|---|---|---|
| | steps/min | HR (95% CI) | steps/min | HR (95% CI) | steps/min | HR (95% CI) |
| **Peak cadence metrics** | | | | | | |
| Peak 1-min cadence | 112.9 | 0.90(0.85,0.95) | 146.9 | 0.69(0.57, 0.84) | — | — |
| Peak 5-min cadence | 96.8 | 0.79 (0.70, 0.89) | 122.5 | 0.62 (0.49, 0.78) | — | — |
| Peak 10-min cadence | 83.3 | 0.78 (0.70, 0.87) | 108.6 | 0.60 (0.48, 0.75) | 131.01 | 0.56 (0.44,0.71) |
| Peak 15-min cadence | 75.4 | 0.78 (0.70, 0.88) | 98.8 | 0.61 (0.49, 0.76) | 123.1 | 0.57 (0.44, 0.72) |
| Peak 20-min cadence | 69.4 | 0.78 (0.70, 0.88) | 91.6 | 0.62 (0.49, 0.77) | 116.6 | 0.57 (0.44, 0.72) |
| Peak 25-min cadence | 64.2 | 0.78 (0.70, 0.88) | 85.9 | 0.61 (0.49, 0.76) | 107.6 | 0.57 (0.44, 0.72) |
| Peak 30-min cadence | 60.2 | 0.78 (0.70, 0.88) | 72.6 | 0.66 (0.55, 0.79) | 101.8 | 0.57 (0.44, 0.72) |
| Peak 60-min cadence | 39.1 | 0.77 (0.69, 0.86) | 54.9 | 0.58 (0.46, 0.72) | 67.9 | 0.54 (0.42, 0.69) |
| **Non-Peak cadence** | | | | | | |

| | | | | | | |
|---|---|---|---|---|---|---|
| **metrics** | | | | | | |
| Purposeful cadence | 52.9 | 0.99 (0.92, 1.07) | 65.4 | 1.04(0.82, 1.32) | 110.5 | 0.98 (0.55, 0.74) |
| Average daily cadence | 1.6 | 0.81 (0.70, 0.93) | 2.4 | 0.70 (0.56, 0.88) | 4.6 | 0.62 (0.47, 0.81) |

Note: '—' indicates that there is no nadir point

**Supplementary Table 5** Hazard Ratio of Cancer Mortality Associated with the Minimum, Median and Maximum Stepping Intensities (in Absolute Cadence Value), Estimated by Peak Cadence and Non-Peak Cadence Metrics

| **Stepping intensity** | minimum cadence | | median cadence | | maximum cadence | |
|---|---|---|---|---|---|---|
| | steps/min | HR (95% CI) | steps/min | HR (95% CI) | steps/min | HR (95% CI) |
| **Peak cadence metrics** | | | | | | |
| Peak 1-min cadence | 137.8 | 0.90 (0.77,1.05) | 146.9 | 0.88 (0.74, 1.05) | — | — |
| Peak 5-min cadence | 122.9 | 0.88 (0.74,1.03) | 123.7 | 0.87 (0.74, 1.03) | — | — |
| Peak 10-min cadence | 98.0 | 0.87 (0.76,0.99) | 46.3 | 1.08 (1.00, 1.17) | — | — |
| Peak 15-min cadence | 85.5 | 0.87 (0.77, 0.98) | 91.5 | 0.85 (0.74, 0.98) | — | — |
| Peak 20-min cadence | 72.9 | 0.89 (0.81, 0.98) | 91.4 | 0.82 (0.70, 0.96) | 135.3 | 0.78 (0.66, 0.94) |
| Peak 25-min cadence | 71.6 | 0.88 (0.79, 0.99) | 85.9 | 0.83 (0.71, 0.97) | — | — |
| Peak 30-min cadence | 66.3 | 0.88 (0.79, 0.98) | 81.0 | 0.82 (0.70, 0.96) | — | — |
| Peak 60-min cadence | 41.9 | 0.89 (0.81, 0.98) | 55.1 | 0.82 (0.70, 0.96) | 79.1 | 0.78 (0.66, 0.93) |
| **Non-Peak cadence** | | | | | | |

| | | | | | | |
|---|---|---|---|---|---|---|
| **metrics** | | | | | | |
| Purposeful cadence | 99.2 | 1,00 (0.65, 1.52) | 65.4 | 1.02 (0.87, 1.20) | 51.5 | 0.99 (0.92, 1.07) |
| Average daily cadence | 13.5 | 0.89 (0.59, 1.33) | 2.4 | 0.82 (0.71, 0.96) | 4.4 | 0.77 (0.64, 0.93) |

Note: '—' indicates that there is no nadir point
